# Phenolic concentrations and carbon/nitrogen ratio in annual shoots of bilberry (*Vaccinium myrtillus*) after simulated herbivory

**DOI:** 10.1371/journal.pone.0298229

**Published:** 2024-03-04

**Authors:** Marcel Schrijvers-Gonlag, Christina Skarpe, Riitta Julkunen-Tiitto, Antonio B. S. Poléo

**Affiliations:** 1 Campus Evenstad, Faculty of Applied Ecology, Agricultural Sciences and Biotechnology, Inland Norway University of Applied Sciences, Koppang, Norway; 2 Department of Environmental and Biological Sciences, Faculty of Science, Forestry and Technology, University of Eastern Finland, Joensuu, Finland; Institute for Biological Research, University of Belgrade, SERBIA

## Abstract

Herbivory can be reduced by the production of defense compounds (secondary metabolites), but generally defenses are costly, and growth is prioritized over defense. While defense compounds may deter herbivory, nutrients may promote it. In a field study in boreal forest in Norway, we investigated how simulated herbivory affected concentrations of phenolics (generally a defense) and the carbon/nitrogen (C/N) ratio in annual shoots of bilberry (*Vaccinium myrtillus*), a deciduous clonal dwarf shrub whose vegetative and generative parts provide forage for many boreal forest animals. We measured concentrations of total tannins, individual phenolics, nitrogen and carbon following several types and intensities of herbivory. We identified 22 phenolics: 15 flavonoids, 1 hydroquinone and 6 phenolic acids. After high levels of herbivory, the total tannin concentration and the concentration of these 22 phenolics together (called total phenolic concentration) were significantly lower in bilberry annual shoots than in the control (natural herbivory at low to intermediate levels). Low-intensive herbivory, including severe defoliation, gave no significantly different total tannin or total phenolic concentration compared with the control. Many individual phenolics followed this pattern, while phenolic acids (deterring insect herbivory) showed little response to the treatments: their concentrations were maintained after both low-intensive and severe herbivory. Contrary to our predictions, we found no significant difference in C/N ratio between treatments. Neither the Carbon:Nutrient Balance hypothesis nor the Optimal Defense hypotheses, theories predicting plant resource allocation to secondary compounds, can be used to predict changes in phenolic concentrations (including total tannin concentration) in bilberry annual shoots after herbivory: in this situation, carbon is primarily used for other functions (e.g., maintenance, growth, reproduction) than defense.

## Introduction

Bilberry (*Vaccinium myrtillus* L.) is an abundant species in boreal forests in Fennoscandia [1–3], and a key understory component influencing soil properties and forest regeneration and succession [4]. This deciduous clonal dwarf shrub has evergreen shoots and grows on nutrient-poor soil by ramets, i.e., orthotropic shoots branching from buds on a rhizome [1, 2, 5–8]. In this study, we called the aboveground orthotropic shoot (the stem) including side shoots and leaves a ramet.

Vegetative and generative parts of bilberry are important forage for many mammal, bird, and insect species in all seasons [9–16]. One of many strategies to minimize herbivory on vegetative plant tissue is the production of defense compounds (defense metabolites, secondary compounds) by plants [17, 18]. Under resource limitation, trade-offs occur among growth, maintenance, storage, reproduction, and defense in plants [19, and references herein]. Some of the theories that predict plant resource allocation to secondary compounds are relevant for bilberry-herbivore interactions. In this study we focused on the Optimal Defense hypotheses (consisting of several (sub)hypotheses, see [20]) and the Carbon:Nutrient Balance hypothesis.

The Optimal Defense hypotheses predict that production of inducible defenses is low when herbivory is absent or nearly absent and increases when herbivory is present, as defenses are costly [20–28]. In general, however, growth is prioritized over defense [19, 29–32, but see 33, and references herein]. Therefore, a severe loss of photosynthetic tissue may not allow for production of defense compounds and may even lead to the breakdown of existing defenses, resulting in lower resistance to herbivory [34–37].

Like many deciduous woody species growing on nutrient-poor soils, bilberry stores carbon in roots and other woody tissue, like stems [19, 30, 38, 39]. The Carbon:Nutrient Balance (CNB) hypothesis predicts a mobilization of these carbon reserves and an increase in the level of carbon-based defense compounds in bilberry after herbivory on shoots and leaves [19, 20, 38, 40]. Phenolic compounds (phenolics) are primarily composed of carbon [41] and can act as such carbon-based defense compounds reducing herbivore performance and herbivory [42–47]. Phenolics include tannins (condensed tannins or proanthocyanidins and hydrolyzable tannins), flavonoids and other small molecular mass phenolics, including cinnamic acids [41, 42, 48]. Many different phenolics have been identified in bilberry stems, shoots, leaves, berries, and rhizomes [49–55]. We expected that the effects of tissue damage, resulting from herbivory or other causes, on phenolic concentration in bilberry vary depending on several factors: the damage type (whether leaves, shoots or the whole ramet is damaged), damage intensity, and the level the actual phenolic can function as a defense against herbivores, as different phenolics have multiple biological functions and efficacy [56–63].

Defense compounds may deter herbivory, while nutrients may promote it [18, 64–68]. Nitrogen concentration in bilberry, which is often used as a proxy for nutrient concentration, increases after browsing in several woody species, often regardless of soil productivity [69–73]. Pruning, the partial or complete removal of stem and/or shoots, reduces bud numbers and increases the root:shoot ratio. This results in decreased competition for nutrients among meristems, causing an increase in new plant tissue nutrient concentration [32, 74–78]. On the other hand, severe defoliation results in a loss of nitrogen [79], or at least in the loss of proportionally more nutrients than carbon, as most nutrients are found within the foliage of deciduous species in the growing season [19]. Furthermore, severe defoliation results in increased fine root mortality [80, 81]. This leads to reduced nutrient absorption which results in a decreased nutrient concentration, especially on nutrient-poor soils [82, 83]. Therefore, we expected that the effects of tissue damage, due to herbivory or other causes, on nitrogen concentration in bilberry vary, depending on type and intensity of damage.

Most research on phenolics in bilberry has focused on berries, although studies on leaves, shoots and stems have been conducted [49, 50, 54, 84–86]. Previous studies of herbivory, nutritional quality and defense responses of bilberry shoots and leaves did not involve controlled clipping experiments, nor measurements of phenolic, nitrogen and carbon concentrations in annual shoots [87–92]. After herbivory, we expected a measurable response in the young tissue of annual shoots [21, 39, 93]. For these reasons, we investigated how simulated herbivory affected phenolic concentrations and the carbon/nitrogen (C/N) ratio, often used as indicator of plant nutritional quality [94, 95], in bilberry annual shoots. We measured total tannin concentration and concentrations of individual phenolics, nitrogen and carbon in bilberry annual shoots after several types and intensities of simulated herbivory. Persson and colleagues [55] performed a simulated browsing study on bilberry investigating responses in phenolic and nitrogen concentrations and C/N ratio in leaves and leafless shoots. Different from Persson and colleagues, who performed different levels of simulated moose (*Alces alces* L.) browsing only, we used three types of simulated herbivory, representing herbivory by large ungulates (eating ramets), herbivory by smaller mammals, birds and insects (eating annual shoots) and herbivory by insects (eating leaves). Our study was performed under ambient herbivory conditions in boreal forest in southeastern Norway in 2014.

Our objective was to examine how different herbivory types (ramet herbivory, annual shoot herbivory, leaf herbivory) and intensities affect the concentration of phenolics (total tannins as well as several small molecular mass phenolics) and nitrogen (nutritional quality) in bilberry annual shoots. We compared our simulated herbivory (from here often just called herbivory) with ambient herbivory, which was at a low to intermediate level. We considered our results in the context of the plant defense theories described above. We predicted that in bilberry annual shoots, the concentration of:

I. phenolics is, at low to intermediate herbivory levels, positively correlated with intensity of herbivory;
II. phenolics is, at high herbivory levels, lower than without herbivory;
III. nitrogen is, at low to intermediate herbivory levels, positively correlated with intensity of herbivory, i.e., the C/N ratio is negatively correlated with intensity of herbivory;
IV. nitrogen is, at high herbivory levels, lower, i.e., the C/N ratio is higher, than without herbivory.

## Methods

### Study area

We conducted our study in coniferous boreal forest at six locations (400–670 m a.s.l.) in the Østerdalen valley close to Evenstad (latitude 61.43 °N, longitude 11.08 °E) in southeastern Norway in 2014. In this year, mean annual temperature was 4.8 °C and total precipitation was 896 mm [96]. The forest was owned by the Norwegian state-owned land and forest enterprise Statskog SF (www.statskog.no), who granted permission to do the experiment, including sampling bilberry plants.

### Study design

#### Field treatments

**Fig 1:**
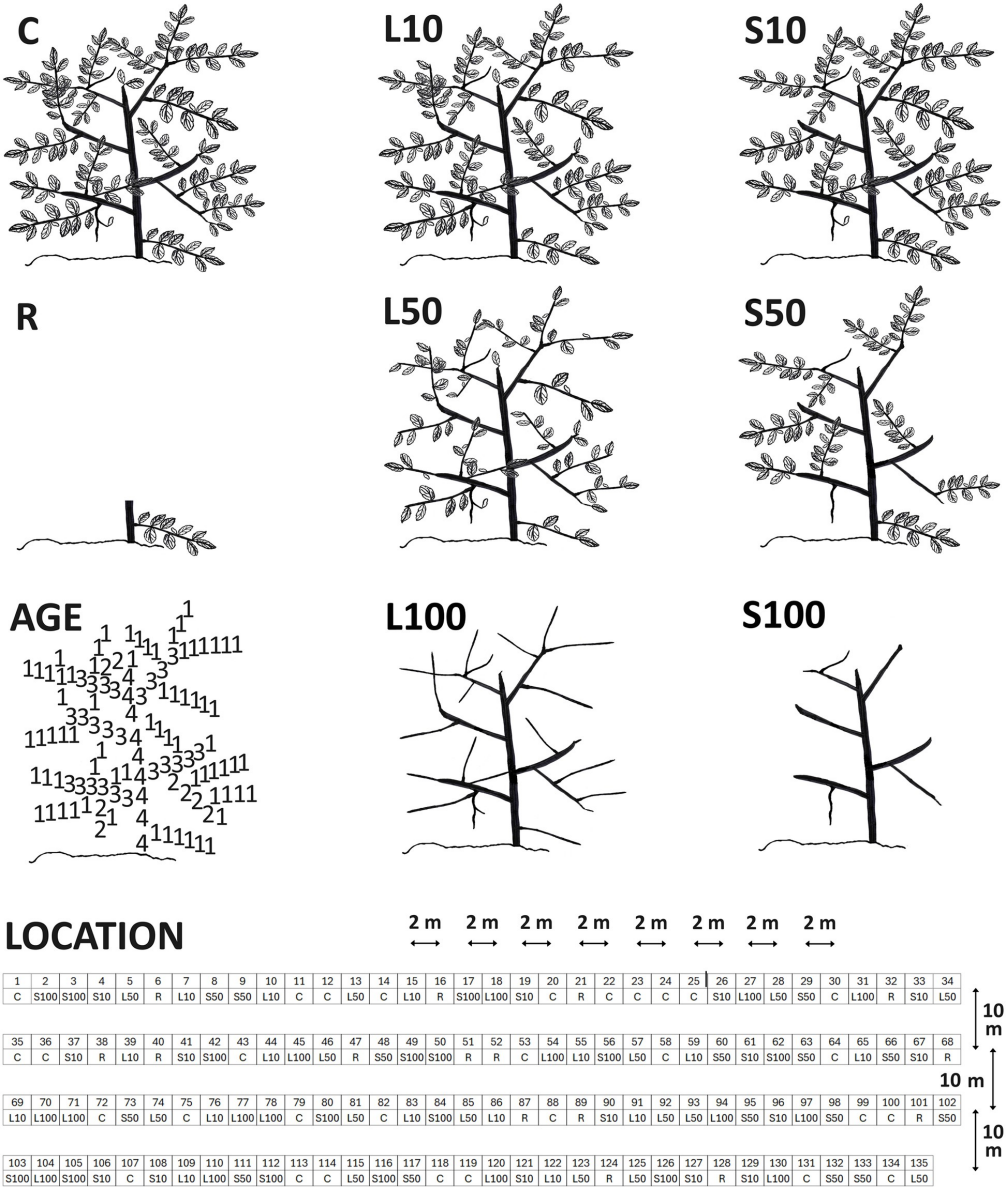
Study design. Schematic bilberry ramet, eight treatments (see text): C (control), three leaf treatments (L), three shoot treatments (S), one ramet treatment (R). 21 annual shoots are visible (1 at the top of the stem, 10 at each side of the stem). AGE: the stem and all shoots (same schematic bilberry as in the treatments) are depicted with numbers: the stem is at least four years old and indicated with 4, shoots at least three years old are indicated with 3, shoots at least two years old are indicated with 2, annual shoots (with leaves) are indicated with 1. NOTE: the upper three-year-old shoot at the right side of the stem and the middle three-year-old shoot at the left side of the stem may also be two years old. LOCATION: at four lines, 34 (upper three lines) and 33 (lower line) ramets are selected: 30 control ramets and 15 ramets for every one of the other 7 treatments, randomly appointed. Approximately 2 m between each ramet and 10 m between each line. This location is one of six locations (namely: “Imsdalen 1”). Drawing: Marcel Schrijvers-Gonlag.

At each location, we used four lines, more or less parallel and spaced by ten m, to select bilberry ramets with approximately two m distance between consecutive ramets (Fig 1). Along each line, we selected 33 or 34 ramets at the beginning of the growing season (May) and marked them with steel wire. Selected ramets had at least ten shoots longer than 1.0 cm from the previous growing season (annual shoots from 2013, S1 File), and no visual signs of extensive herbivory (most ramets had some past herbivory signs), so the initial herbivory level for all ramets was low. In total we selected 135 ramets at each location. We divided the ramets within each location randomly (S1 File) into four treatment groups: control (n = 30), abbreviated to C, representing ambient, initially low, herbivory only; ’leaves cut’ (n = 45), abbreviated to L, representing additional herbivory by insects; ’annual shoots cut’ (n = 45), abbreviated to S, representing additional herbivory by insects and small-sized vertebrates; and ’ramet cut’ (n = 15), abbreviated to R, representing additional herbivory by large ungulates. At all six locations, we removed leaves by hand (treatment L) at three different intensities: 10% from 15 ramets, 50% from another 15 ramets and 100% from the remaining 15 ramets (S1 File). At five locations, we removed annual shoots by hand (treatment S) at similar intensities (10%, 50% and 100%; n = 15 for each), and we cut the ramet in treatment R by removing 90% of the ramet with garden scissors (Fig 1). In total this resulted in eight treatments: ’control’ (C: ambient, initially low, herbivory), ’leaves cut’ (3 intensities: L10, L50, L100), ’annual shoots cut’ (3 intensities: S10, S50, S100), ’ramet cut’ (R). Ramets in C that experienced severe herbivory between selecting and harvesting, were excluded from our analyses: therefore, all control ramets experienced herbivory at low to intermediate levels (ambient herbivory). The shoots were removed and ramets cut on 24–27 May and leaves were removed in the period 21 June– 2 July. In our experiment, we considered C as herbivory at the lowest level. Within L, L10 represented leaf herbivory at a low level, L50 represented leaf herbivory at an intermediate level and L100 represented leaf herbivory at a high level. Similar with S: within S, S10 represented annual shoot herbivory at a low level, S50 represented annual shoot herbivory at an intermediate level and S100 represented annual shoot herbivory at a high level. We considered R as herbivory at the highest level and S100 as herbivory at the second highest level in our experiment: judging after proportion of biomass removed, these two treatments were the two most severe herbivory treatments in our study.

#### Bilberry sampling

All ramets (n = 750) were harvested towards the end of the growing season (19–28 August) by cutting the stem at ground level with garden scissors. The ramets were dried for minimum 48 hours in a drying oven (Binder FED 720 E2, Germany) at 30 °C, before the dried ramets were stored in a dark and dry place at room temperature. From each location, we randomly selected a minimum of five dried ramets from each herbivory treatment (including control), resulting in 232 ramets in total. From each of these ramets, we randomly selected five annual shoots (S1 File), continued drying these annual shoots for minimum 24 hours at 30 °C and stored them in a dark and dry place at room temperature, prior to preparation and analyses of tannin, phenolic, carbon and nitrogen concentrations.

### Chemical analyses

#### Bilberry shoots

Before analyses of acetone-soluble tannins, methanol-soluble phenolics, and total carbon and nitrogen, the shoots were cut in fragments of maximum 0.5 cm and for each ramet we transferred these subsamples to a 2 ml or 7 ml vial with three stainless steel beads (2.8 mm) to pulverize the tissue; with large subsamples (approximately 200, 300 and 400 mg; all weight measurements in this study: scale Sartorius SE2, d = 0.1 μg) we used four, five or six beads, respectively. The shoot fragments were pulverized by the beads using a Precellys 24 homogenizer (Bertin Technologies, France): 25 s at 5500 rpm, 15 times with two minutes in between. When handling the shoots, we used disposable latex gloves.

#### Shoot tannins

The shoots were analyzed for acetone (70%)-soluble tannins (e.g., hydrolyzable tannins and polymeric condensed tannins (proanthocyanidins)) [97, 98: S1, 99] with a spectrophotometer (Spectronic 20 Genesys; Spectronic Instruments, USA). We slightly adjusted the acid butanol assay for proanthocyanidins [100] to measure tannins in our subsamples (S2 File). To relate tannin concentration in our subsamples to measured absorbance (at 550 nm) we built a standard reference curve, using Sephadex LH-20 (GE Healthcare Bio-Sciences AB, Sweden) for tannin purification [98: S1, 101].

#### Shoot phenolics

Methanol-soluble phenolics (e.g., flavonoids and phenolic acids) were extracted from the shoots and quantified using high performance liquid chromatography (HPLC) with injection volume 10 μl (Agilent series 1100) and identified using a UHPLC quadrupole time-of-flight liquid chromatograph–mass spectrometer (Agilent Technologies, 6540 UHD Accurate-Mass Q-TOF LC/MS, 1290 Infinity) as described by Nissinen and colleagues [102] (S3 File). Compounds that could not be identified were not used in this study. We used D(-)-Salicin min. 99% CHR (Aldrich-Chemie, West-Germany) in methanol (100%) as an internal standard in two out of five subsamples to evaluate extraction efficiency (S3 File).

#### Shoot carbon and nitrogen

The shoots were analyzed for carbon and nitrogen (total concentration (mg/g, dry weight) after destruction; micro CN-analyzer (thermo), Chemical Biological Soil Laboratory (quality system based on the ISO-17025 standard), Wageningen University, July 2016).

### Statistical analyses

The total tannin absorbance measurements were averaged per subsample and with the standard reference curve and subsample weight these subsample means were converted to concentrations (mg tannins/g shoots, dry weight), which were used in further analyses. In our HPLC analyses we used the concentration (mg/g, dry weight) of every identified phenolic as the response variable in our modeling, calculated as: ((rf x area) / weight) / (inj / tot) where rf is the HPLC response factor for the actual phenolic at the used wavelength, area is the peak area in the HPLC result table (mAU*s) at the used wavelength, weight is the initial shoot subsample weight (mg), inj is the HPLC injection volume (10 or 15 μl) and tot is the total volume (600 μl) in which the subsample was dissolved (300 μl methanol + 300 μl purified water, S3 File). The HPLC response factor is the ratio between the concentration of a specific compound (mg/g) and the response of the detector (area: mAU*s) to this compound at a specific wavelength; we used response factors previously determined using standards with known concentrations (S1 Table). Before analyses, phenolic concentrations were converted to 100% to recover losses in the extraction procedure (S3 File). When no value in the HPLC result table was present for a phenolic, we used a concentration of 0 mg/g, although often a small peak on the HPLC chromatogram was visible.

**Fig 2:**
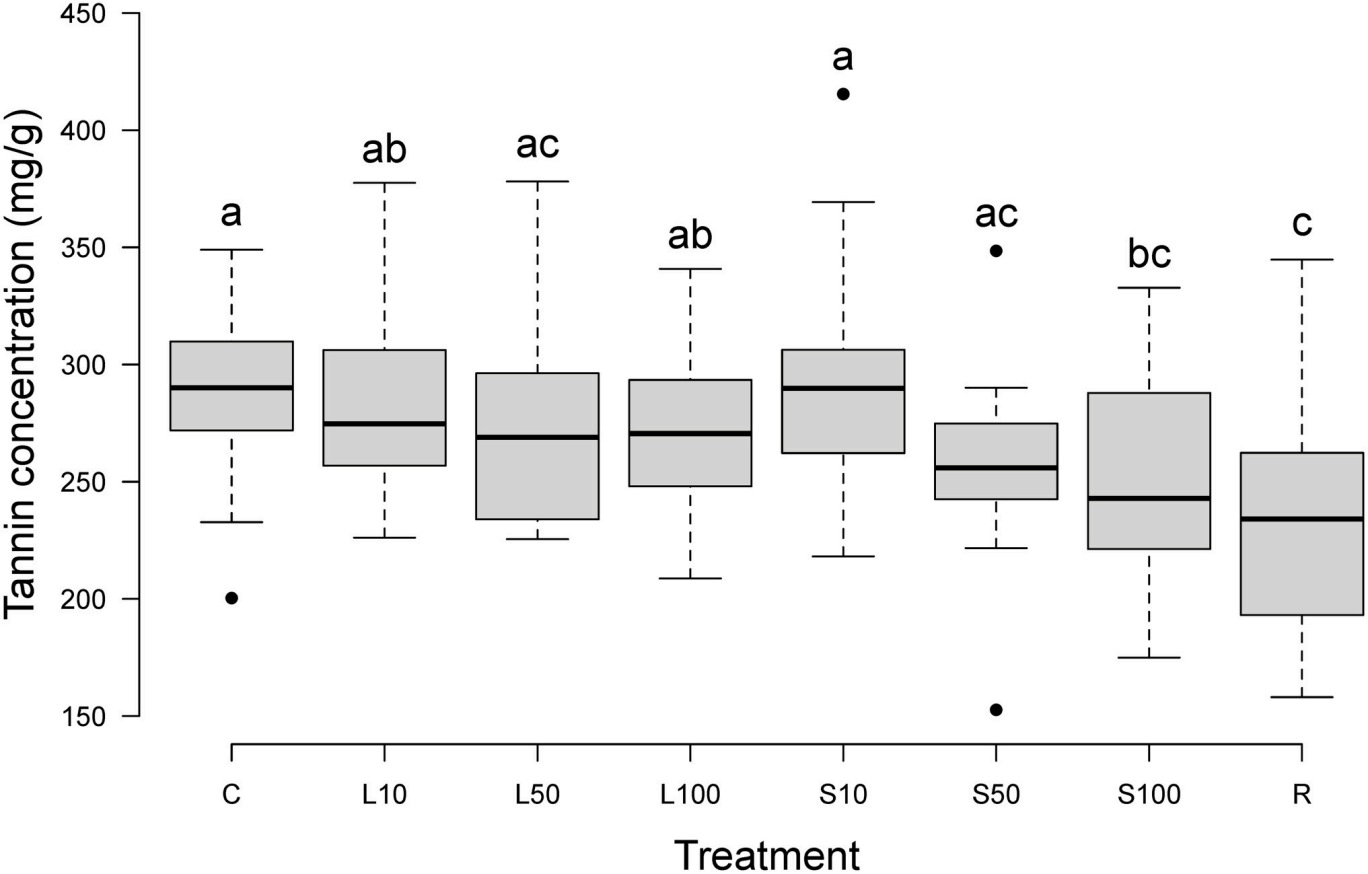
Tannin concentration in bilberry annual shoots after simulated herbivory. Boxplot with total concentration (mg/g, dry weight), n = 200, every treatment n = 25. Treatments: see text. The bottom and top of each box indicate the first and third quartiles. Bold horizontal lines within each box indicate median values. The plot whiskers extend to the most extreme data point which is no more than 1.5 times the interquartile range away from the box; extreme data points more than 1.5 times the interquartile range away from the box are indicated with black points. Treatments with the same letter above the box are not different from each other (*P* > 0.05).

**Fig 3:**
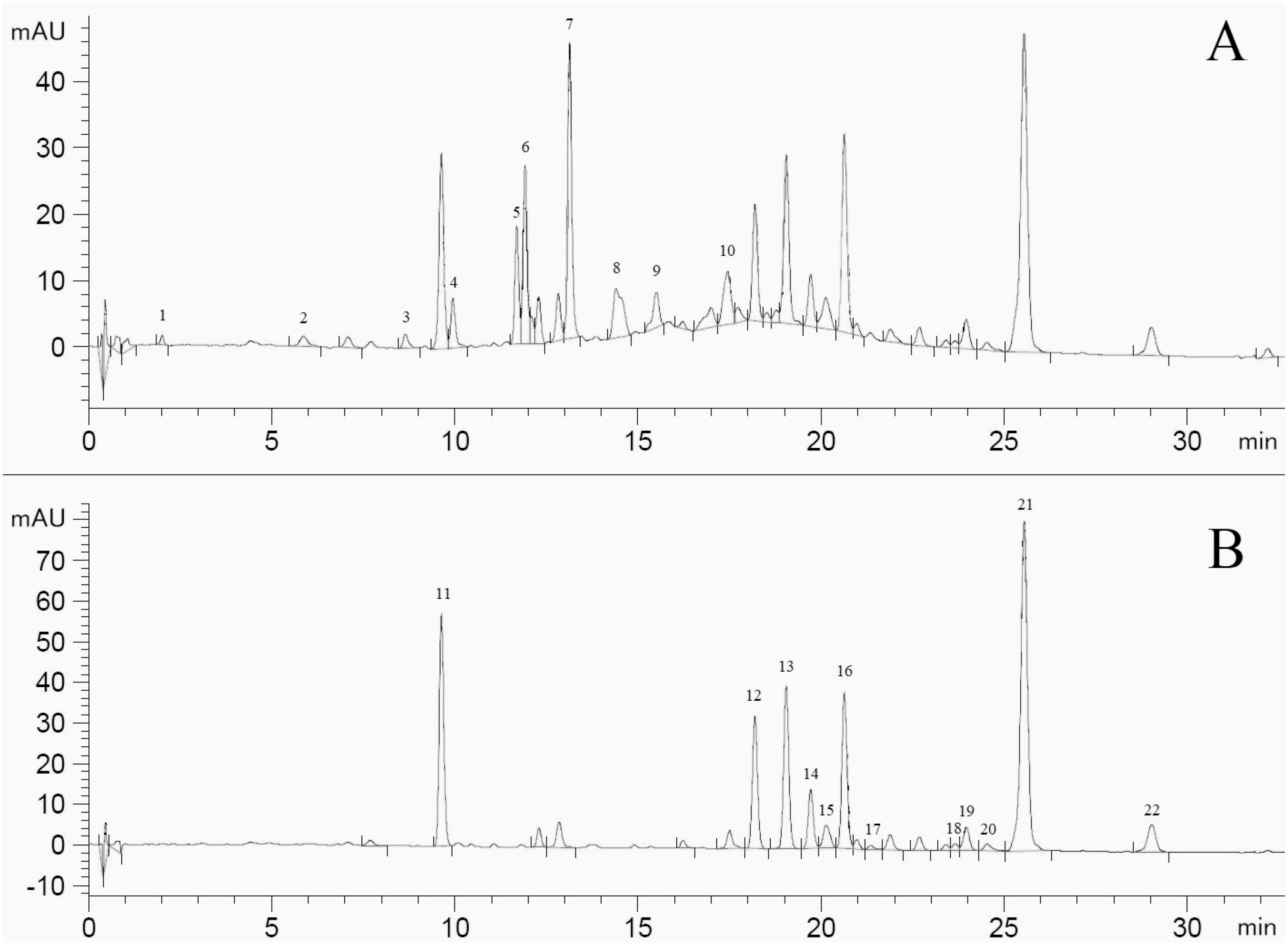
HPLC chromatogram of phenolics in bilberry annual shoots. The chromatograms shown here (**A** wavelength 280 nm, **B** wavelength 320 nm; retention time (x-axis) in minutes (min), response (y-axis) in mAU (AU = absorption units)) are from the subsample which was used to identify the peaks with mass spectrometry. Phenolics identified (for footnotes, see S1 Table): 1. protocatechuic acid derivative, 2. arbutin derivative^7^, 3. gallocatechin derivative, 4. procyanidin 1, 5. procyanidin 2, 6. epicatechin (formerly called: (-)-epicatechin), 7. procyanidin 3, 8. procyanidin 4, 9. procyanidin 5, 10. procyanidin 6, 11. chlorogenic acid, 12. para-hydroxycinnamic acid derivative 1, 13. cinnamic acid derivative, 14. para-hydroxycinnamic acid derivative 2, 15. hyperin^1^, 16. quercetin 3-glucuronide^5^, 17. quercetin 3-arabinoside^4^, 18. kaempferol 3-glucoside^2^, 19. quercitrin^6^, 20. isorhamnetin 3-glucoside, 21. para-hydroxycinnamic acid derivative 3, 22. monocoumaroyl-isoquercitrin^3,8^.

**Fig 4:**
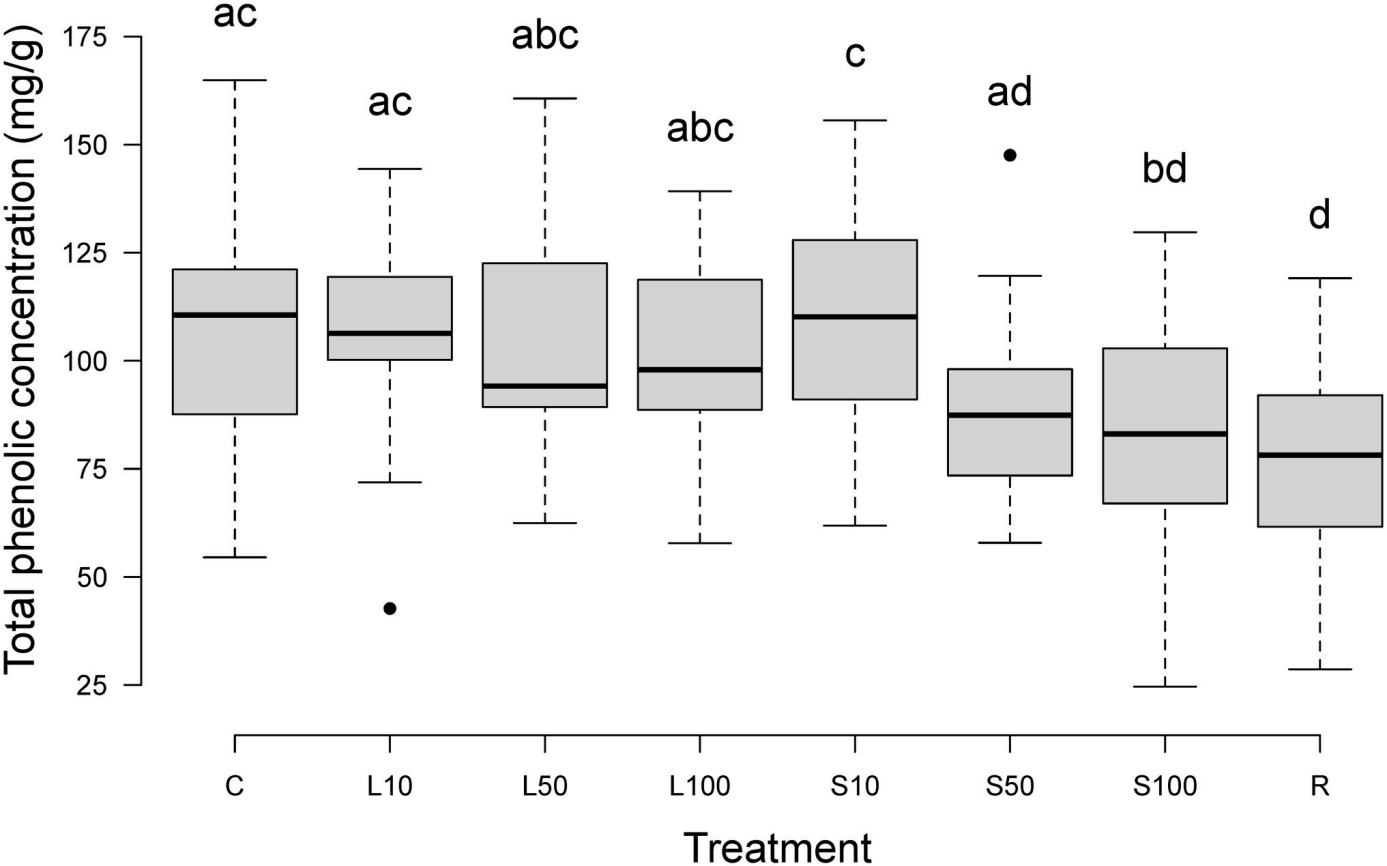
Total phenolic concentration in bilberry annual shoots after simulated herbivory. Boxplot with total phenolic concentration (mg/g, dry weight), n = 200, every treatment n = 25. Treatments: see text. The bottom and top of each box indicate the first and third quartiles. Bold horizontal lines within each box indicate median values. The plot whiskers extend to the most extreme data point which is no more than 1.5 times the interquartile range away from the box; extreme data points more than 1.5 times the interquartile range away from the box are indicated with black points. Treatments with the same letter above the box are not different from each other (*P* > 0.05).

**Fig 5:**
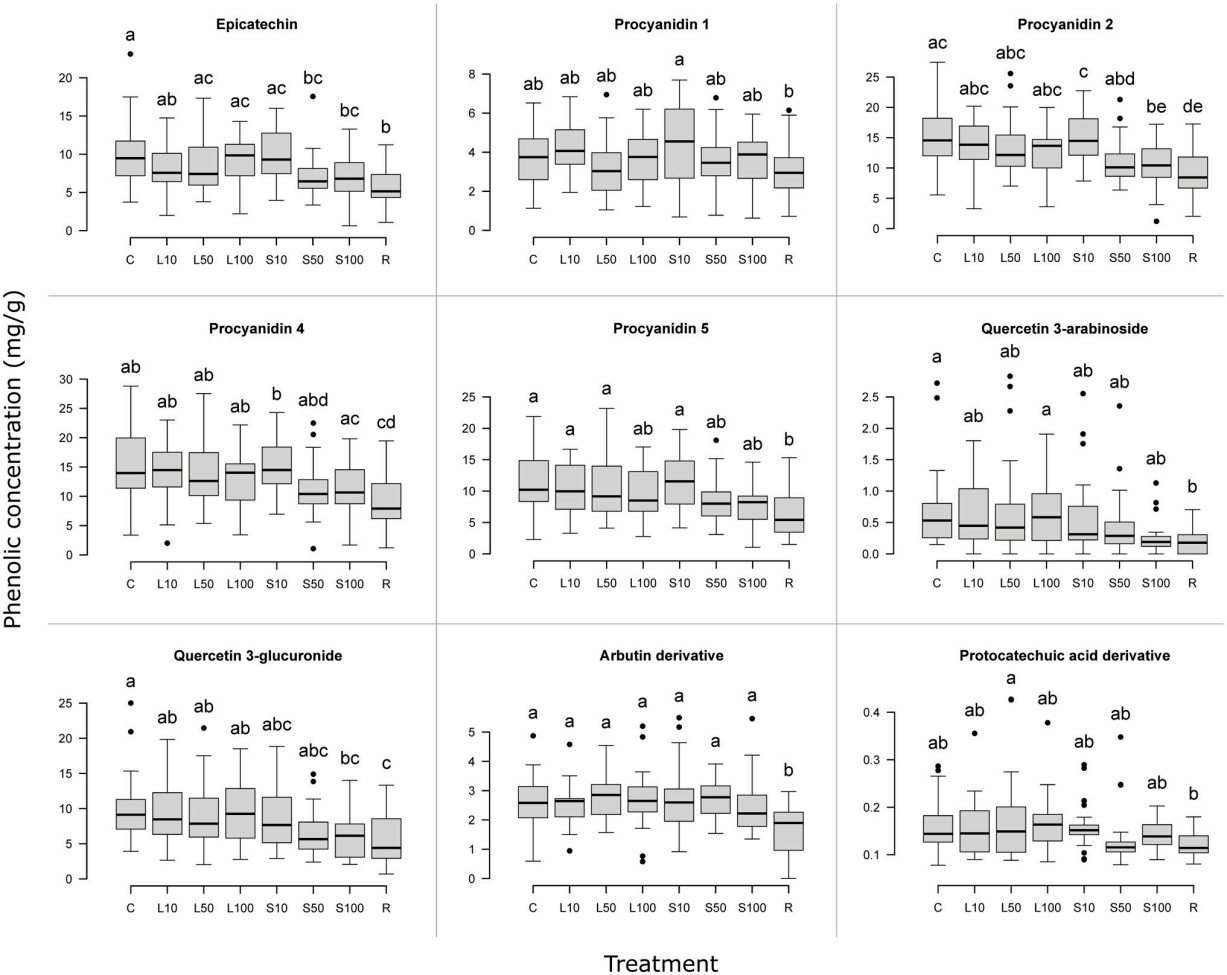
Individual phenolic concentration in bilberry annual shoots after simulated herbivory. Boxplots with individual phenolic concentration (mg/g, dry weight). Only phenolics with at least one treatment significantly different from another treatment are shown (n = 200, every treatment n = 25). Treatments: see text. The bottom and top of each box indicate the first and third quartiles. Bold horizontal lines within each box indicate median values. The plot whiskers extend to the most extreme data point which is no more than 1.5 times the interquartile range away from the box; extreme data points more than 1.5 times the interquartile range away from the box are indicated with black points. Treatments with the same letter above the box are not different from each other (*P* > 0.05).

Differences between treatments were investigated with a one-way ANOVA test. In all ANOVA analyses we used equal sample sizes across groups, to avoid inflation of error rates and to guarantee homogeneity of variance [103]. If necessary, samples were randomly removed to obtain balanced sample sizes. We used the total tannin concentration, the concentration of each identified phenolic and the concentration of all identified phenolics together as response variables in predictions I and II. We also used a one-way ANOVA test to investigate differences between treatments on the response variable C/N ratio (predictions III and IV). When the ANOVA test indicated a significant difference (we used a significance level of 5%), differences between groups were investigated with Tukey’s HSD post-hoc test. We used the package ‘emmeans’ in the software ‘R’ to calculate some general statistics and to further investigate the relationship between several response variables and treatments [104, 105]. Figs 2–5 were created with the R-package ‘svglite’ [106] and the software ‘Inkscape’ (version 1.2.1). All model analyses were performed in R (version 4.1.2, 4.2.2, 4.2.3 and 4.3.1) [105].

## Results

### Shoot tannins

On average, bilberry annual shoots consisted of 25–30% tannins (dry weight; Table 1). The intensity of herbivory affected tannin concentrations (ANOVA: F_7,192_ = 6.18, *P* < 0.001; Fig 2; Table 1). S100 and R resulted in significantly lower tannin concentrations than C. All other treatments did not differ significantly from each other nor from C (Fig 2).

**Table 1:** Tannins, phenolics, carbon, nitrogen, and C/N in bilberry annual shoots after simulated herbivory. Mean concentration (mg/g ± se (standard error of the sample mean), dry weight) of tannins, 22 phenolics (see text), carbon (C) and nitrogen (N) and mean C/N ratio (± standard error), per treatment (see text). For all treatments together (All) also the sd (standard deviation of the sample) is given. Number of observations between parentheses.

Treatment	Tannins	Phenolics	Carbon (C)	Nitrogen (N)	C/N
C	291.1 ± 7.1 (33)	106.4 ± 4.1 (33)	498 ± 2.0 (33)	12.27 ± 0.36 (33)	41.9 ± 1.2 (33)
L10	278.2 ± 7.1 (33)	106.1 ± 4.2 (32)	497 ± 2.0 (33)	12.40 ± 0.36 (33)	40.9 ± 1.2 (33)
L50	275.7 ± 7.2 (32)	100 9 ± 4.2 (32)	498 ± 2.1 (32)	12.64 ± 0.37 (32)	40.1 ± 1.2 (32)
L100	274.4 ± 7.2 (32)	101.4 ± 4.2 (32)	498 ± 2.0 (32)	12.26 ± 0.37 (32)	41.7 ± 1.2 (32)
S10	292.8 ± 8.2 (25)	110.2 ± 4.7 (25)	500 ± 2.4 (25)	12.17 ± 0.42 (25)	42.1 ± 1.4 (25)
S50	257.1 ± 8.2 (25)	88.6 ± 4.7 (25)	498 ± 2.4 (25)	12.94 ± 0.42 (25)	39.6 ± 1.4 (25)
S100	249.3 ± 8.0 (26)	84.9 ± 4.6 (26)	504 ± 2.3 (26)	12.76 ± 0.41 (26)	40.7 ± 1.4 (26)
R	235.7 ± 8.0 (26)	75.1 ± 4.6 (26)	500 ± 2.3 (26)	12.62 ± 0.41 (26)	41.3 ± 1.4 (26)
All	270.5 ± 2.9, sd = 44.1 (232)	97.5 ± 1.7, sd = 25.9 (231)	499 ± 0.8, sd = 11.8 (232)	12.50 ± 0.14, sd = 2.08 (232)	41.0 ± 0.5, sd = 6.9 (232)

### Shoot phenolics

We identified 22 phenolics: 15 flavonoids, 1 hydroquinone and 6 phenolic acids (Fig 3). The recovery of the internal standard was around 95%: min = 58.9%, mean = 94.5%, max = 107.5%, sd = 6.1% (n = 90). In one subsample the recovery of the internal standard was 58.9%, quite different from all others. Therefore, we excluded this subsample from the phenolic analyses. Without this subsample, the recovery of the internal standard changed to: min = 83.1%, mean = 94.9%, max = 107.5%, sd = 4.7% (n = 89).

The phenolic concentration of all these 22 identified phenolics together (analyzed together) is from here called total phenolic concentration. On average, almost 10% of bilberry annual shoots consisted of these 22 phenolics (dry weight; Table 1). Compared to C and L10, which had very similar total phenolic concentrations, all other treatments except S10 resulted in lower mean total phenolic concentrations (Table 1; S2 Table). The differences between C, L10 and S10 were not significant (Fig 4), but a significant difference in total phenolic concentration between one or more other treatments was present (ANOVA: F_7,192_ = 6.64, *P* < 0.001). Within L, the total phenolic concentration did not differ significantly, but it did within S (Fig 4). S100 and R resulted in significantly lower total phenolic concentrations than C. R resulted in the lowest mean (S2 Table) and median (Fig 4) total phenolic concentration.

As the total phenolic concentration is the sum of all identified phenolic concentrations, many of these individual phenolics showed a similar pattern: R resulted in the lowest mean phenolic concentration in 15 phenolics (68%). Considering R and S100 together, this number increased to 19 phenolics (86%) (S2 Table). Investigating significant differences between treatments, one or more herbivory treatments resulted in significantly different phenolic concentrations in nine phenolics; phenolic acids showed little response to the treatments (Table 2). Considering the leaf (L) removal treatments only, there were no significant differences in individual phenolic concentration between treatments, nor were any L treatments significantly different from C (Fig 5). Considering the annual shoot (S) removal treatments, in two phenolics (procyanidin 2 and 4) the phenolic concentration was significantly lower in S100 than in S10. Compared to the other treatments, S10 resulted in the highest mean phenolic concentration in eight phenolics (36%) and in the total phenolic concentration (S2 Table), although no significant difference between S10 and C was present in any of the phenolics. In three phenolics (epicatechin, procyanidin 2 and quercetin 3-glucuronide) S100 resulted in a significantly lower concentration than C (Fig 5). In seven phenolics, R resulted in a significantly lower phenolic concentration than C (Fig 5).

**Table 2:** Differences in individual phenolic concentration in bilberry annual shoots between simulated herbivory treatments. The number of differences (#) between treatments (*P* < 0.05) is given for 22 phenolics separately, and for all 22 phenolics analyzed together (n = 200, every treatment n = 25).

Flavonoids	#
Epicatechin	6
Gallocatechin derivative	ns
Hyperin	0
Isorhamnetin 3-glucoside	ns
Kaempferol 3-glucoside	ns
Monocoumaroyl-isoquercitrin^1^	ns
Procyanidin 1	1
Procyanidin 2	8
Procyanidin 3	ns
Procyanidin 4	6
Procyanidin 5	4
Procyanidin 6	ns
Quercetin 3-arabinoside	2
Quercetin 3-glucuronide	5
Quercitrin	ns
***Sum flavonoids***	*32*
**Hydroquinones**	
Arbutin derivative	7
***Sum hydroquinones***	*7*
**Phenolic acids**	
Chlorogenic acid	ns
Cinnamic acid derivative	ns
Para-hydroxycinnamic acid derivative 1	ns
Para-hydroxycinnamic acid derivative 2	ns
Para-hydroxycinnamic acid derivative 3	ns
Protocatechuic acid derivative	1
***Sum phenolic acids***	*1*
***Sum all individual phenolics***	*40*
**All 22 phenolics together**	9

^1^Monocoumaroyl-isoquercitrin: identification uncertain.

If the ANOVA test result *P* < 0.05 but the Tukey’s HSD test gave only *P*_adj_ values > 0.05 a ‘0’ is shown in the table. ANOVA test results *P* > 0.05 show ‘ns’ in the table (irrespective of the Tukey’s HSD test result).

### Shoot carbon and nitrogen

On average, half of the bilberry annual shoots (dry weight) consisted of carbon and slightly over 1% consisted of nitrogen (Table 1). There was little variation in carbon and nitrogen concentration and in C/N ratio between treatments (Table 1). We found no significant difference in C/N ratio between treatments (ANOVA: F_7,192_ = 0.40, *P* = 0.90). Analyses of carbon concentration and nitrogen concentration yielded similar results: no significant difference between treatments (carbon: ANOVA: F_7,192_ = 1.04, *P* = 0.40; nitrogen: ANOVA: F_7,192_ = 0.37, *P* = 0.92).

## Discussion

### Shoot tannins and total phenolics

The two most severe herbivory treatments, concerning removed biomass, resulted in significantly lower tannin and total phenolic concentrations than the control, while less severe herbivory treatments did not differ significantly from the control. These results support our second prediction but suggest that little to intermediate loss of photosynthetic tissue does not increase carbon-based defense compound concentrations in bilberry, contrary to our first prediction. Several factors may contribute to these results.

First, not all phenolics respond to herbivory as predicted by the CNB hypothesis, as many phenolics compete with proteins for the nitrogen containing precursor phenylalanine as described by Jones & Hartley [107] in their Protein Competition Model (PCM) [42, 108, 109]. As the boreal forest is a nitrogen-limited ecosystem [110–113], competition for nitrogen between biosynthesis of proteins and of many phenolics is expected in boreal forest ecosystems. Therefore, the nutrient-poor soil may not provide sufficient nitrogen for bilberry to increase these secondary compound concentrations while continuing protein demanding primary processes as growth and reproduction.

As bilberry is a clonal plant, connected ramets may translocate compounds from nondefoliated to defoliated ramets, as has been documented in perennial graminoid species and herbs [114–116, and references herein]. Translocation of carbohydrates or even phenolics between connected ramets may be another reason for the absence of a correlation between leaf herbivory and subsequent phenolic concentrations in bilberry annual shoots. Also, high fine root mortality may not, or to a lesser extent, occur in clonal bilberry, which possibly translocates carbohydrates between connected ramets to maintain its root activity.

Furthermore, our simulated leaf herbivory (mechanical wounding by hand) is not equivalent to leaf herbivory by insects, birds, or small mammals [27, 117–125, reviewed by 126]. Although most of these studies indicate that simulated leaf and shoot herbivory performed by mechanical wounding induces a less pronounced plant response, such simulated herbivory generally does induce a plant response [see the aformentioned references and 87, 127, 128], as the general response of plants to wounding and herbivore damage is essentially the same [129]. This is particularly true in nutrient-poor sites [130], like our study system. Nevertheless, this indicates that bilberry responses to simulated leaf herbivory may differ from responses to natural herbivory, which can further contribute to our observed absence of a correlation between simulated leaf herbivory and subsequent phenolic concentrations in bilberry annual shoots.

Additionally, this observed absence can be due to other reasons. A response can have been counteracted by transport of existing phenolics from shoots to leaves, as some plant species store phenolics in shoots which are transported to leaves following herbivory [127]–although such reallocation of phenolics may not be very important [107]. Furthermore, the time between our leaf herbivory treatments and bilberry ramet harvesting was 48–68 days. Possibly, bilberry only responds with a short-term response that was no longer detectable after 48 days. For instance, in another woody species, the condensed tannin concentration returned to pre-herbivory values less than 66 hours after herbivory [131, see also 132]. An alternative possible reason is a very delayed response: responses remain undetectable until at least 68 days after the treatment. This last option seems very unlikely in terms of plant fitness, but cannot be ruled out with the data available. Experiments measuring how long induction lasts in different bilberry tissues are needed to support or reject these speculations.

Another possible reason for our observed results is that an herbivory-induced change in phenolics occurs in other plant parts, e.g., leaves, and is not detectable in annual shoots. This seems unlikely, as Persson and colleagues found that bilberry leaves and bilberry leafless shoots were comparable in their response to simulated moose herbivory, at least for flavonoids and condensed tannins [55]. In contrast with our results, Persson and colleagues found an increase in flavonoid and condensed tannin concentration in bilberry shoots with increasing simulated moose herbivory. Possibly their results were influenced by a side-effect of the treatment: a more open canopy resulted in more solar radiation which could have induced production of secondary compounds, as has been found and discussed in other studies [50, 55, 133–139, and references herein].

### Shoot individual phenolics

In seven phenolics, R resulted in a significantly lower phenolic concentration than C, while in fifteen phenolics no significant difference between the control and other treatments was present. The concentrations of all phenolic acids found in our study were unaffected by severe herbivory. Interestingly, these phenolics are known to deter herbivory by insects: all reduce larval growth rate, some also promote larval mortality and chlorogenic acid even shows strong anti-nutritive properties against various invertebrate herbivores, including adult beetles and grasshopper nymphs [45, 140–147]. This indicates that bilberry responds to severe herbivory by maintaining concentrations of phenolics which deter herbivory on a certain level. As we could not find information about biological functions related to herbivory for other specific phenolics identified in our study, we don’t know how bilberry responds to severe herbivory in the case of phenolics which promote herbivory (possibly by decreasing their concentrations?). Herbivory experiments with specific phenolics are necessary to support or reject these speculations.

We did not find (+)-catechin in our bilberry annual shoots, as has been found in other bilberry studies [49, 50]. In the HPLC chromatogram (Fig 3), (+)-catechin, if present, comes shortly after chlorogenic acid. This means that when a large quantity of chlorogenic acid is present, as with our subsamples (Fig 3, S2 Table), the chlorogenic acid peak overlaps with the peak of (+)-catechin and it is not possible to separate the latter from the former, especially when only little (+)-catechin is present. Therefore, unidentified amounts of (+)-catechin may have been present in our subsamples, but if so, (+)-catechin was present in much lower amounts than epicatechin (S2 Table).

### Shoot carbon and nitrogen

The carbon and nitrogen concentrations and C/N ratio in our study are comparable with results from other studies [6, 50, 136, 148, 149] but differ from bilberry nitrogen concentrations found by Selås and colleagues [150]. Our results show that both the carbon and nitrogen concentration, as well as the C/N ratio, in bilberry annual shoots are not affected by herbivory. These findings do not support our predictions III and IV. Apparently, mechanisms that either increase or decrease nutrient concentration after herbivory (see Introduction), cause this overall result. Additionally, in clonal bilberry carbohydrates may be translocated from source ramets to connecting ramets under herbivory pressure, and to their root system, to compensate for a lack of carbon (see before). This may prevent an increase in fine root mortality and, consequently, a decrease in nutrient concentration. Thus, clonality can further explain the lack of support for our predictions III and IV.

Another possible reason is, as with phenolics (see before), that a change in C/N ratio does not occur in bilberry annual shoots but in other plant parts, e.g., leaves, as shown in other woody species [70, 72, 75, 95, 151] (although Laine and Henttonen [148] did not find a correlation between microtine density and nitrogen concentration in bilberry leaves). As we do not have data about carbon and nitrogen concentrations in plant parts other than annual shoots, we cannot rule out this possibility.

Finally, Flower-Ellis [6] reported much variation in nitrogen concentration between long, vegetative shoots and short, predominantly flowering shoots, as well as in ramets from different ages and positions in the stand (causing variation in light and water conditions). Such variation may obscure effects from herbivory.

### Study design

In this study, we removed annual shoots in four treatments, at different intensities: S10, S50, S100 and R. Only with the last two treatments (S100 and R), all (or almost all in some R treatments) annual shoots were removed. Approximately three months after removal, we harvested annual shoots from the ramets: therefore, only with S100 and R the harvested annual shoots were all (or almost all in some R treatments) new shoots, grown after the clipping event had occurred. In all other treatments, most likely the analyzed annual shoots had all (L treatments) or partly (S10: around 90 percent, S50: around 50 percent) been present at the ramet before the clipping event took place. Interestingly, only at high herbivory levels (S100 and R), we found a significant difference in tannin concentration and total phenolic concentration compared to the control. This means that all treatments from which we analyzed many older annual shoots (from before the clipping event) did not yield a significant difference in phenolic concentration in the annual shoots compared to the control. Although many of these annual shoots probably were not fully grown at the time of clipping and therefore also their tissue had (partly) developed after the clipping event took place, this means that we analyzed annual shoots in S10, S50 and all L treatments, that were present before the clipping event occurred–at least an important part of them. If a chemical response to the treatment does not occur in older shoots but only, or mainly, occurs in new tissue (this we don’t know) this shortcoming in our study design has serious consequences for our results regarding to the S10, S50 and all L treatments.

### Defense and other metabolic processes

As our results do not support our first prediction (I: phenolic concentration is, at low to intermediate herbivory levels, positively correlated with intensity of herbivory) but do support our second prediction (II: phenolic concentration is, at high herbivory levels, lower than without herbivory), we conclude that after herbivory, bilberry uses carbon primarily for functions other that defense. This is no more than a speculation, as we have no metric of growth (as total biomass or compensatory growth), or metabolic processes other than phenolic concentrations. Possibly, little herbivory may be almost inconsequential for plant fitness and responses may be absent, or non-detectable, or only morphological, not chemical. Severe herbivory may force bilberry to divert resources from other pools, as existing defense chemical compounds, to compensate for biomass losses. Experiments which specifically focus on morphological responses (as compensatory growth) and reproduction, preferably also chemical responses, after herbivory, are needed to support or reject our speculation.

## Conclusions

We conclude that neither the Carbon:Nutrient Balance hypothesis nor the Optimal Defense hypotheses can be used to predict changes in phenolic concentrations (including total tannin concentration) after herbivory in bilberry annual shoots. After herbivory, bilberry uses carbon primarily for functions other than defense (e.g., maintenance, growth, reproduction). Herbivory experiments focusing on morphological responses and reproduction are necessary to further investigate this response. Furthermore, we conclude that bilberry responds to severe herbivory by maintaining concentrations of specific phenolics, which deter herbivory, on a certain level, while decreasing concentrations of other phenolics. Herbivory experiments with specific phenolics, to clearify their function as anti-herbivore compound (i.e., do they affect bilberry’s palatability to herbivores), are necessary to further investigate this response.

## Supporting information

S1 File Annual shoots and random selection. (PDF)

S2 File Standard reference curve and tannin color test. (PDF)

S3 File Quantifying phenolics using HPLC. (PDF)

S1 Table Response factors. (PDF)

S2 Table Phenolic concentrations after simulated herbivory. (PDF)

## References

[1] Fremstad E. Vegetasjonstyper i Norge. 2 ed. Trondheim: Norsk institutt for naturforskning; 1997. 279 p.

[2] Mossberg B, Stenberg L. Nordens flora. Stockholm: Bonnier Fakta; 2018. 976 p.

[3] Boonstra R, Andreassen HP, Boutin S, Hušek J, Ims RA, Krebs CJ, et al. Why do the boreal forest ecosystems of northwestern Europe differ from those of western North America? Bioscience. 2016;66(9):722–34. doi: 10.1093/biosci/biw080 28533563 PMC5421309

[4] Nilsson MC, Wardle DA. Understory vegetation as a forest ecosystem driver: evidence from the northern Swedish boreal forest. Front Ecol Environ. 2005;3(8):421–8. doi: 10.2307/3868658

[5] Ritchie JC. *Vaccinium myrtillus* L. J Ecol. 1956;44(1):291–9. doi: 10.2307/2257181

[6] Flower-Ellis JGK. Age structure and dynamics in stands of bilberry (*Vaccinium myrtillus* L.) [PhD dissertation]. Stockholm: Royal college of forestry; 1971.

[7] Nestby R, Percival D, Martinussen I, Opstad N, Rohloff J. The European blueberry (*Vaccinium myrtillus* L.) and the potential for cultivation. A review. The European Journal of Plant Science and Biotechnology. 2011;5(1):5–16.

[8] Tolvanen A, Laine K. Effects of reproduction and artificial herbivory on vegetative growth and resource levels in deciduous and evergreen dwarf shrubs. Can J Bot. 1997;75(4):656–66. doi: 10.1139/b97-073

[9] Cederlund G, Ljunqvist H, Markgren G, Stålfelt F. Foods of moose and roe-deer at Grimsö in central Sweden. Results of rumen content analyses. Swedish Wildlife Research—Viltrevy. 1980;11(4):169–247.

[10] Spidsø TK. Food selection by Willow Grouse *Lagopus lagopus* chicks in northern Norway. Ornis Scand. 1980;11:99–105.

[11] Viro P, Sulkava S. Food of the bank vole in northern Finnish spruce forests. Acta Theriol (Warsz). 1985;30(9–20):259–66.

[12] Atlegrim O. Exclusion of birds from bilberry stands—impact on insect larval density and damage to the bilberry. Oecologia. 1989;79(1):136–9. doi: 10.1007/BF00378251 28312824

[13] Hjältén J, Danell K, Ericson L. Hare and vole browsing preferences during winter. Acta Theriol (Warsz). 2004;49(1):53–62. doi: 10.1007/Bf03192508

[14] Wegge P, Kastdalen L. Habitat and diet of young grouse broods: resource partitioning between Capercaillie (*Tetrao urogallus*) and Black Grouse (*Tetrao tetrix*) in boreal forests. J Ornithol. 2008;149(2):237–44. doi: 10.1007/s10336-007-0265-7

[15] Soininen EM, Ravolainen VT, Brathen KA, Yoccoz NG, Gielly L, Ims RA. Arctic small rodents have diverse diets and flexible food selection. PLoS ONE. 2013;8(6). doi: 10.1371/journal.pone.0068128 23826371 PMC3694920

[16] Frøstrup JC. Hare og harejakt. Oslo: Teknologisk forl.; 1996. 123 p.

[17] Karban R, Baldwin IT. Induced responses to herbivory. Chicago, IL, USA: University of Chicago Press; 1997. X+320 p.

[18] Skarpe C, Hester AJ. Plant traits, browsing and grazing herbivores, and vegetation dynamics. In: Gordon IJ, Prins HHT, editors. The ecology of browsing and grazing. Berlin: Springer; 2008. p. 217–47.

[19] Herms DA, Mattson WJ. The dilemma of plants—to grow or defend. Q Rev Biol. 1992;67(3):283–335.

[20] Stamp N. Out of the quagmire of plant defense hypotheses. Q Rev Biol. 2003;78(1):23–55. doi: 10.1086/367580 12661508

[21] McKey D. Adaptive patterns in alkaloid physiology. Am Nat. 1974;108(961):305–20.

[22] Rhoades DF. Evolution of plant chemical defense against herbivores. Herbivores: their interaction with secondary plant metabolites. New York: Academic Press; 1979. p. 3–54.

[23] Fagerström T, Larsson S, Tenow O. On optimal defence in plants. Funct Ecol. 1987;1(2):73–81.

[24] Karban R, Agrawal AA, Thaler JS, Adler LS. Induced plant responses and information content about risk of herbivory. Trends Ecol Evol. 1999;14(11):443–7. doi: 10.1016/s0169-5347(99)01678-x 10511721

[25] Kessler A. The information landscape of plant constitutive and induced secondary metabolite production. Curr Opin Insect Sci. 2015;8:47–53. doi: 10.1016/j.cois.2015.02.002 32846677

[26] Bryant JP, Julkunen-Tiitto R. Ontogenic development of chemical defense by seedling resin birch: Energy cost of defense production. J Chem Ecol. 1995;21(7):883–96. doi: 10.1007/BF02033796 24234407

[27] Mithöfer A, Boland W, Maffei ME. Chemical ecology of plant–insect interactions. In: Parker J, editor. Annual Plant Reviews volume 34: Molecular aspects of plant disease resistance 2009. p. 261–91.

[28] Massad TJ, Trumbore SE, Ganbat G, Reichelt M, Unsicker S, Boeckler A, et al. An optimal defense strategy for phenolic glycoside production in *Populus trichocarpa*-isotope labeling demonstrates secondary metabolite production in growing leaves. New Phytol. 2014;203(2):607–19. doi: 10.1111/nph.12811 . 24739022

[29] Waring RH, Pitman GB. Modifying lodgepole pine stands to change susceptibility to mountain pine beetle attack. Ecology. 1985;66(3):889–97. doi: 10.2307/1940551

[30] Tuomi J, Niemelä P, Stuart Chapin F, Bryant JP, Sirén S. Defensive responses of trees in relation to their carbon/nutrient balance. Mechanisms of woody plant defenses against insects. New York, NY: Springer-Verlag; 1988. p. 57–72.

[31] Gayler S, Grams TEE, Heller W, Treutter D, Priesack E. A dynamical model of environmental effects on allocation to carbon-based secondary compounds in juvenile trees. Ann Bot. 2008;101(8):1089–98. doi: 10.1093/aob/mcm169 17693454 PMC2710266

[32] Skarpe C, Van der Wal R. Effects of simulated browsing and length of growing season on leaf characteristics and flowering in a deciduous Arctic shrub, *Salix polaris*. Arct Antarct Alp Res. 2002;34(3):282–6. doi: 10.1080/15230430.2002.12003495

[33] Li R, Zhang J, Li J, Zhou G, Wang Q, Bian W, et al. Prioritizing plant defence over growth through WRKY regulation facilitates infestation by non-target herbivores. eLife. 2015;4:e04805. doi: 10.7554/eLife.04805 26083713 PMC4491539

[34] Coley PD, Bryant JP, Chapin FS. Resource availability and plant antiherbivore defense. Science. 1985;230(4728):895–9. doi: 10.1126/science.230.4728.895 17739203

[35] Barz W, Hoesel W. Metabolism and degradation of phenolic compounds in plants. In: Swain T, Harborne JB, Van Sumere CF, editors. Biochemistry of plant phenolics. New York: Plenum Press; 1979. p. 339–69.

[36] Gershenzon J. Changes in the levels of plant secondary metabolites under water and nutrient stress. In: Timmermann BN, Steelink C, Loewus FA, editors. Phytochemical Adaptations to Stress. Boston, MA: Springer US; 1984. p. 273–320.

[37] Kohi EM, De Boer WF, Slot M, Van Wieren SE, Ferwerda JG, Grant RC, et al. Effects of simulated browsing on growth and leaf chemical properties in *Colophospermum mopane* saplings. African Journal of Ecology. 2010;48(1):190–6. doi: 10.1111/j.1365-2028.2009.01099.x

[38] Tuomi J, Fagerström T, Niemelä P. Carbon allocation, phenotypic plasticity, and induced defenses. In: Tallamy DW, Raupp MJ, editors. Phytochemical induction by herbivores. New York/ Chichester/ Brisbane/ Totonto/ Singapore: Wiley; 1991. p. 85–104.

[39] Lähdesmäki P, Pakonen T, Saari E, Laine K, Havas P. Environmental factors affecting basic nitrogen metabolism and seasonal levels of various nitrogen fractions in tissues of bilberry, *Vaccinium myrtillus*. Holarctic Ecology. 1990;13(1):19–30. doi: 10.1111/j.1600-0587.1990.tb00585.x

[40] Bryant JP, Chapin FS, Klein DR. Carbon nutrient balance of boreal plants in relation to vertebrate herbivory. Oikos. 1983;40(3):357–68. doi: 10.2307/3544308

[41] Engbersen JFJ, De Groot Æ. Bio-organische chemie. Fifth ed. Wageningen: Pudoc; 1992. 596 p.

[42] Vermerris W, Nicholson RL. Phenolic compound biochemistry: Springer; 2008. XII+276 p.

[43] Bennett RN, Wallsgrove RM. Secondary metabolites in plant defence mechanisms. New Phytol. 1994;127(4):617–33. doi: 10.1111/j.1469-8137.1994.tb02968.x 33874382

[44] Dearing MD. Effects of *Acomastylis rossii* tannins on a mammalian herbivore, the North American pika, *Ochotona princeps*. Oecologia. 1996;109(1):122–31. doi: 10.1007/s004420050066 28307602

[45] Felton GW, Donato KK, Broadway RM, Duffey SS. Impact of oxidized plant phenolics on the nutritional quality of dietar protein to a noctuid herbivore, *Spodoptera exigua*. J Insect Physiol. 1992;38(4):277–85. doi: 10.1016/0022-1910(92)90128-Z

[46] Tahvanainen J, Niemelä P, Henttonen H. Chemical aspects of herbivory in boreal forest—feeding by small rodents, hares, and cervids. In: Palo RT, Robbins CT, editors. Plant defenses against mammalian herbivory. Boca Raton: CRC Press; 1991. p. 115–31.

[47] Tahvanainen J, Julkunen-Tiitto R, Kettunen J. Phenolic glycosides govern the food selection pattern of willow feeding leaf beetles. Oecologia. 1985;67(1):52–6. doi: 10.1007/BF00378451 28309845

[48] Seigler DS. Plant secondary metabolism. Boston: Kluwer; 1998. IX+759 p.

[49] Bujor OC, Le Bourvellec C, Volf I, Popa VI, Dufour C. Seasonal variations of the phenolic constituents in bilberry (*Vaccinium myrtillus* L.) leaves, stems and fruits, and their antioxidant activity. Food Chem. 2016;213:58–68. doi: 10.1016/j.foodchem.2016.06.042 27451155

[50] Nybakken L, Selås V, Ohlson M. Increased growth and phenolic compounds in bilberry (*Vaccinium myrtillus* L.) following forest clear-cutting. Scand J For Res. 2013;28(4):319–30. doi: 10.1080/02827581.2012.749941

[51] Latti AK, Riihinen KR, Jaakola L. Phenolic compounds in berries and flowers of a natural hybrid between bilberry and lingonberry (*Vaccinium* x *intermedium* Ruthe). Phytochemistry. 2011;72(8):810–5. doi: 10.1016/j.phytochem.2011.02.015 21382629

[52] Riihinen K, Jaakola L, Karenlampi S, Hohtola A. Organ-specific distribution of phenolic compounds in bilberry (*Vaccinium myrtillus*) and ’northblue’ blueberry (*Vaccinium corymbosum* x *V*. *angustifolium*). Food Chem. 2008;110(1):156–60. doi: 10.1016/j.foodchem.2008.01.057 26050178

[53] Martz F, Jaakola L, Julkunen-Tiitto R, Stark S. Phenolic composition and antioxidant capacity of bilberry (*Vaccinium myrtillus*) leaves in northern Europe following foliar development and along environmental gradients. J Chem Ecol. 2010;36(9):1017–28. doi: 10.1007/s10886-010-9836-9 20721607

[54] Witzell J, Gref R, Näsholm T. Plant-part specific and temporal variation in phenolic compounds of boreal bilberry (*Vaccinium myrtillus*) plants. Biochem Syst Ecol. 2003;31(2):115–27. doi: 10.1016/S0305-1978(02)00141-2

[55] Persson IL, Julkunen-Tiitto R, Bergström R, Wallgren M, Suominen O, Danell K. Simulated moose (*Alces alces* L.) browsing increases accumulation of secondary metabolites in bilberry (*Vaccinium myrtillus* L.) along gradients of habitat productivity and solar radiation. J Chem Ecol. 2012;38(10):1225–34. doi: 10.1007/s10886-012-0209-4 23143636

[56] Agati G, Azzarello E, Pollastri S, Tattini M. Flavonoids as antioxidants in plants: Location and functional significance. Plant Sci. 2012;196:67–76. doi: 10.1016/j.plantsci.2012.07.014 23017900

[57] Asplund J, van Zuijlen K, Roos RE, Birkemoe T, Klanderud K, Lang SI, et al. Contrasting responses of plant and lichen carbon-based secondary compounds across an elevational gradient. Funct Ecol. 2021;35(2):330–41. doi: 10.1111/1365-2435.13712

[58] Gauslaa Y. Lichen palatability depends on investments in herbivore defence. Oecologia. 2005;143(1):94–105. doi: 10.1007/s00442-004-1768-z 15619096

[59] Lokvam J, Kursar TA. Divergence in structure and activity of phenolic defenses in young leaves of two co-occurring *Inga* species. J Chem Ecol. 2005;31(11):2563–80. doi: 10.1007/s10886-005-7614-x 16273429

[60] Bernays EA. Plant tannins and insect herbivores: an appraisal. Ecol Entomol. 1981;6(4):353–60. doi: 10.1111/j.1365-2311.1981.tb00625.x

[61] Solhaug KA, Gauslaa Y. Secondary lichen compounds as protection against excess solar radiation and herbivores. In: Lüttge U, Beyschlag W, Büdel B, Francis D, editors. Progress in Botany 73. Berlin, Heidelberg: Springer; 2012. p. 283–304.

[62] Molnár K, Farkas E. Current results on biological activities of lichen secondary metabolites: a review. Zeitschrift für Naturforschung C. 2010;65(3–4):157–73. doi: 10.1515/znc-2010-3-401 20469633

[63] Horie Y. Effects of various fractions of mulberry leaves on feeding of the silkworm, *Bombyx mori* L. J Sericult Sc Jpn. 1962;31(4):258–64. doi: 10.11416/kontyushigen1930.31.258

[64] Virjamo V, Julkunen-Tiitto R, Henttonen H, Hiltunen E, Karjalainen R, Korhonen J, et al. Differences in vole preference, secondary chemistry and nutrient levels between naturally regenerated and planted Norway spruce seedlings. J Chem Ecol. 2013;39(10):1322–34. doi: 10.1007/s10886-013-0352-6 24105602

[65] Mattson WJ. Herbivory in relation to plant nitrogen content. Annu Rev Ecol Syst. 1980;11(1):119–61. doi: 10.1146/annurev.es.11.110180.001003

[66] Lundberg P, Åström M. Low nutritive quality as a defense against optimally foraging herbivores. Am Nat. 1990;135(4):547–62. doi: 10.1086/285061

[67] Schädler M, Jung G, Auge H, Brandl R. Palatability, decomposition and insect herbivory: patterns in a successional old-field plant community. Oikos. 2003;103(1):121–32. doi: 10.1034/j.1600-0706.2003.12659.x

[68] Baraza E, Villalba JJ, Provenza FD. Nutritional context influences preferences of lambs for foods with plant secondary metabolites. Appl Anim Behav Sci. 2005;92(4):293–305. doi: 10.1016/j.applanim.2004.11.010

[69] Oksanen L, Oksanen T, Lukkari A, Sirén S. The role of phenol-based inducible defense in the interaction between tundra populations of the vole *Clethrionomys rufocanus* and the dwarf shrub *Vaccinium myrtillus*. Oikos. 1987;50(3):371–80. doi: 10.2307/3565498

[70] Du Toit JT, Bryant JP, Frisby K. Regrowth and palatability of *Acacia* shoots following pruning by African savanna browsers. Ecology. 1990;71(1):149–54. doi: 10.2307/1940255

[71] Peinetti HR, Menezes RSC, Coughenour MB. Changes induced by elk browsing in the aboveground biomass production and distribution of willow (*Salix monticola* Bebb): their relationships with plant water, carbon, and nitrogen dynamics. Oecologia. 2001;127(3):334–42. doi: 10.1007/s004420000593 28547104

[72] Olofsson J, Dahlgren J, Witzell J. Grey-sided voles increase the susceptibility of Northern willow, *Salix glauca*, to invertebrate herbivory. Ecoscience. 2007;14(1):48–54. doi: 10.2980/1195-6860(2007)14[48:Gvitso]2.0.Co;2

[73] Scogings PF, Hjältén J, Skarpe C, Hattas D, Zobolo A, Dziba L, et al. Nutrient and secondary metabolite concentrations in a savanna are independently affected by large herbivores and shoot growth rate. Plant Ecol. 2014;215(1):73–82. doi: 10.1007/s11258-013-0279-6

[74] Moorby J, Wareing P. Ageing in woody plants. Ann Bot. 1963;27(2):291–308. doi: 10.1093/oxfordjournals.aob.a083846

[75] Danell K, Huss-Danell K. Feeding by insects and hares on birches earlier affected by moose browsing. Oikos. 1985;44(1):75–81. doi: 10.2307/3544046

[76] Danell K, Haukioja E, Huss-Danell K. Morphological and chemical responses of mountain birch leaves and shoots to winter browsing along a gradient of plant productivity. Ecoscience. 1997;4(3):296–303. doi: 10.1080/11956860.1997.11682408

[77] Scogings P, Macanda M. *Acacia karroo* responses to early dormant season defoliation and debarking by goats in a semi-arid subtropical savanna. Plant Ecol. 2005;179(2):193–206. doi: 10.1007/s11258-004-7809-1

[78] Löyttyniemi K. On repeated browsing of Scots pine saplings by moose (*Alces alces*). Silva Fenn. 1985;19(4):387–91. doi: 10.14214/sf.a15431

[79] Roitto M, Markkola A, Julkunen-Tiitto R, Sarjala T, Rautio P, Kuikka K, et al. Defoliation-induced responses in peroxidases, phenolics, and polyamines in Scots pine (*Pinus sylvestris* L.) needles. J Chem Ecol. 2003;29(8):1905–18. doi: 10.1023/A:1024858413437 12956514

[80] Kozlowski TT. Growth and development of trees. Volume II: Cambial growth, root growth, and reproductive growth. Ney York & London: Academic Press; 1971. XIV+514 p.

[81] Tuomi J, Niemelä P, Siren S. The Panglossian paradigm and delayed inducible accumulation of foliar phenolics in mountain birch. Oikos. 1990;59(3):399–410. doi: 10.2307/3545152

[82] Tuomi J, Niemelä P, Haukioja E, Sirén S, Neuvonen SJO. Nutrient stress: an explanation for plant anti-herbivore responses to defoliation. Oecologia. 1984;61(2):208–10. doi: 10.1007/BF00396762 28309413

[83] Myers JH, Williams KS. Lack of short or long term inducible defenses in the red alder: western tent caterpillar system. Oikos. 1987;48(1):73–8. doi: 10.2307/3565690

[84] Hokkanen J, Mattila S, Jaakola L, Pirttila AM, Tolonen A. Identification of phenolic compounds from lingonberry (*Vaccinium vitis-idaea* L.), bilberry (*Vaccinium myrtillus* L.) and hybrid bilberry (*Vaccinium* x *intermedium* Ruthe L.) leaves. J Agric Food Chem. 2009;57(20):9437–47. doi: 10.1021/jf9022542 19788243

[85] Liu P, Lindstedt A, Markkinen N, Sinkkonen J, Suomela J-P, Yang B. Characterization of metabolite profiles of leaves of bilberry (*Vaccinium myrtillus* L.) and lingonberry (*Vaccinium vitis-idaea* L.). J Agric Food Chem. 2014;62(49):12015–26. doi: 10.1021/jf503521m 25408277

[86] Ștefănescu BE, Szabo K, Mocan A, Crişan G. Phenolic compounds from five Ericaceae species leaves and their related bioavailability and health benefits. Molecules. 2019;24(11):2046. doi: 10.3390/molecules24112046 . 31146359 PMC6600139

[87] Seldal T, Hegland SJ, Rydgren K, Rodriguez-Saona C, Töpper JP. How to induce defense responses in wild plant populations? Using bilberry (*Vaccinium myrtillus*) as example. Ecol Evol. 2017:1–8. doi: 10.1002/ece3.2687 28331586 PMC5355179

[88] Moe SR, Gjørvad IR, Eldegard K, Hegland SJ. Ungulate browsing affects subsequent insect feeding on a shared food plant, bilberry (*Vaccinium myrtillus*). Basic Appl Ecol. 2018;31:44–51. doi: 10.1016/j.baae.2018.05.015

[89] Hegland SJ, Seldal T, Lilleeng MS, Rydgren K. Can browsing by deer in winter induce defence responses in bilberry (*Vaccinium myrtillus*)? Ecol Res. 2016;31(3):441–8. doi: 10.1007/s11284-016-1351-1

[90] Hegland SJ, Rydgren K, Seldal T. The response of *Vaccinium myrtillus* to variations in grazing intensity in a Scandinavian pine forest on the island of Svanøy. Canadian Journal of Botany. 2005;83(12):1638–44. doi: 10.1139/b05-132

[91] Lilleeng MS, Hegland SJ, Rydgren K, Moe SR. Ungulate herbivory reduces abundance and fluctuations of herbivorous insects in a boreal old-growth forest. Basic Appl Ecol. 2021;56:11–21. doi: 10.1016/j.baae.2021.06.006

[92] Benevenuto RF, Seldal T, Polashock J, Moe SR, Rodriguez-Saona C, Gillespie MAK, et al. Molecular and ecological plant defense responses along an elevational gradient in a boreal ecosystem. Ecol Evol. 2020;10(5):2478–91. doi: 10.1002/ece3.6074 32184995 PMC7069305

[93] McKey D. The distribution of secondary compounds within plants. In: Rosenthal GA, Janzen DH, editors. Herbivores: their interaction with secondary plant metabolites. New York: Academic Press; 1979. p. 55–133.

[94] Agrawal AA, Fishbein M. Plant defense syndromes. Ecology. 2006;87(sp7):S132–S49. doi: 10.1890/0012-9658(2006)87[132:pds]2.0.co;2 16922309

[95] Nordkvist M, Klapwijk MJ, Edenius L, Gershenzon J, Schmidt A, Bjorkman C. Trait-mediated indirect interactions: Moose browsing increases sawfly fecundity through plant-induced responses. Ecol Evol. 2019;9(18):10615–29. doi: 10.1002/ece3.5581 31624570 PMC6787786

[96] Wheather and climate data Norway [Internet]. Norwegian Meteorological Institute. 2021 [cited 11 January 2021]. https://seklima.met.no/observations.

[97] Julkunen-Tiitto R, Sorsa S. Testing the effects of drying methods on willow flavonoids, tannins, ans salicylates. J Chem Ecol. 2001;27(4):779–89. doi: 10.1023/A:1010358120482 11446300

[98] Salminen JP, Karonen M. Chemical ecology of tannins and other phenolics: we need a change in approach. Funct Ecol. 2011;25(2):325–38. doi: 10.1111/j.1365-2435.2010.01826.x

[99] Salih EYA, Julkunen-Tiitto R, Lampi A-M, Kanninen M, Luukkanen O, Sipi M, et al. *Terminalia laxiflora* and *Terminalia brownii* contain a broad spectrum of antimycobacterial compounds including ellagitannins, ellagic acid derivatives, triterpenes, fatty acids and fatty alcohols. J Ethnopharmacol. 2018;227:82–96. doi: 10.1016/j.jep.2018.04.030 29733942

[100] Hagerman AE. Acid butanol assay for proanthocyanidins. In: Hagerman AE, editor. The tannin handbook. Miami University, Oxford. 2002. Accessed in 2021, URL: http://www.users.miamioh.edu/hagermae/2002.

[101] Hagerman AE. Sephadex LH 20. In: Hagerman AE, editor. The tannin handbook. Miami University, Oxford. 2002. Accessed in 2021, URL: http://www.users.miamioh.edu/hagermae/2002.

[102] Nissinen K, Virjamo V, Randriamanana T, Sobuj N, Sivadasan U, Mehtätalo L, et al. Responses of growth and leaf phenolics in European aspen (*Populus tremula*) to climate change during juvenile phase change. Can J For Res. 2017;47(10):1350–63. doi: 10.1139/cjfr-2017-0188

[103] Kao LS, Green CE. Analysis of variance: is there a difference in means and what does it mean? J Surg Res. 2008;144(1):158–70. doi: 10.1016/j.jss.2007.02.053 17936790 PMC2405942

[104] Lenth R. Package ’emmeans’: Estimated Marginal Means, aka Least-Squares Means. R package version 1.3.2. 2019. https://cran.r-project.org/package=emmeans.

[105] R Core Team. R: A language and environment for statistical computing. R Foundation for Statistical Computing Vienna, Austria. 2021. https://www.R-project.org/.

[106] Wickham H, Henry L, Pedersen TL, Luciani TJ, Decorde M, Lise V. Package ’svglite’: An ’SVG’ graphics device. R package version 2.1.0. 2022. https://CRAN.R-project.org/package=svglite.

[107] Jones CG, Hartley SE. A protein competition model of phenolic allocation. Oikos. 1999;86(1):27–44. doi: 10.2307/3546567

[108] Haukioja E, Ossipov V, Koricheva J, Honkanen T, Larsson S, Lempa K. Biosynthetic origin of carbon-based secondary compounds: cause of variable responses of woody plants to fertilization? Chemoecology. 1998;8(3):133–9. doi: 10.1007/s000490050018

[109] Koricheva J, Barton K. Temporal changes in plant secondary metabolite production: patterns, causes and consequences. In: Iason GR, Dicke M, Hartley SE, editors. The ecology of plant secondary metabolites. From genes to global processes. Ecological Reviews. Cambridge: Cambridge University Press; 2012. p. 34–55.

[110] Elser JJ, Bracken MES, Cleland EE, Gruner DS, Harpole WS, Hillebrand H, et al. Global analysis of nitrogen and phosphorus limitation of primary producers in freshwater, marine and terrestrial ecosystems. Ecol Lett. 2007;10(12):1135–42. doi: 10.1111/j.1461-0248.2007.01113.x 17922835

[111] Menge DNL, Hedin LO, Pacala SW. Nitrogen and phosphorus limitation over long-term ecosystem development in terrestrial ecosystems. PLoS ONE. 2012;7(8):e42045–e. doi: 10.1371/journal.pone.0042045 . 22870281 PMC3411694

[112] Vitousek PM, Howarth RW. Nitrogen limitation on land and in the sea—how can it occur. Biogeochemistry. 1991;13(2):87–115. doi: 10.1007/BF00002772

[113] Tamm CO. Nitrogen in terrestrial ecosystems: questions of productivity, vegetational changes, and ecosystem stability. Billings WD, Golley F, Lange OL, Olson JS, Remmert H, editors. Berlin: Springer-Verlag; 1991. XII+116 p.

[114] Marshall C. Source-sink relations of interconnected ramets. In: Van Groenendaal J, De Kroon H, editors. Clonal growth in plants: regulation and function. The Hague: Academic Publishing; 1990. p. 23–41.

[115] Jonsdottir IS, Callaghan TV. Intraclonal translocation of ammonium and nitrate nitrogen in *Carex bigelowii* Torr. ex Schwein. using 15 N and nitrate reductase assays. New Phytol. 1990;114(3):419–28. doi: 10.1111/j.1469-8137.1990.tb00409.x 33873967

[116] Welker JM, Briske DD, Weaver RW. Nitrogen-15 partitioning within a three generation tiller sequence of the bunchgrass *Schizachyrium scoparium*: response to selective defoliation. Oecologia. 1987;74(3):330–4. doi: 10.1007/BF00378925 28312468

[117] Walling LL. The myriad plant responses to herbivores. Journal of Plant Growth Regulation. 2000;19(2):195–216. doi: 10.1007/s003440000026 11038228

[118] Mithöfer A, Wanner G, Boland W. Effects of feeding *Spodoptera littoralis* on lima bean leaves. II. Continuous mechanical wounding resembling insect feeding is sufficient to elicit herbivory-related volatile emission. Plant Physiol. 2005;137(3):1160–8. doi: 10.1104/pp.104.054460 15728342 PMC1065415

[119] Hartley SE, Firn RD. Phenolic biosynthesis, leaf damage, and insect herbivory in birch (*Betula pendula*). J Chem Ecol. 1989;15(1):275–83. doi: 10.1007/BF02027789 24271442

[120] Miles PW. Aphid saliva. Biol Rev. 1999;74(1):41–85. Epub 1999/02/01. doi: 10.1017/S0006323198005271

[121] Will T, Van Bel AJE. Induction as well as suppression: How aphid saliva may exert opposite effects on plant defense. Plant Signaling & Behavior. 2008;3(6):427–30. doi: 10.4161/psb.3.6.5473 19704587 PMC2634323

[122] Rooke T. Growth responses of a woody species to clipping and goat saliva. African Journal of Ecology. 2003;41(4):324–8. doi: 10.1111/j.1365-2028.2003.00478.x

[123] Ohse B, Hammerbacher A, Seele C, Meldau S, Reichelt M, Ortmann S, et al. Salivary cues: simulated roe deer browsing induces systemic changes in phytohormones and defence chemistry in wild-grown maple and beech saplings. Funct Ecol. 2017;31:340–9. doi: 10.1111/1365-2435.12717

[124] Liu JS, Wang L, Wang DL, Bonser SP, Sun F, Zhou YF, et al. Plants can benefit from herbivory: stimulatory effects of sheep saliva on growth of *Leymus chinensis*. PLoS ONE. 2012;7(1). doi: 10.1371/journal.pone.0029259 22235277 PMC3251555

[125] Howe GA, Jander G. Plant immunity to insect herbivores. Annu Rev Plant Biol. 2008;59(1):41–66. doi: 10.1146/annurev.arplant.59.032607.092825 . 18031220

[126] Waterman JM, Cazzonelli CI, Hartley SE, Johnson SN. Simulated herbivory: the key to disentangling plant defence responses. Trends Ecol Evol. 2019;34(5):447–58. doi: 10.1016/j.tree.2019.01.008 30824196

[127] Clausen TP, Reichardt PB, Bryant JP, Werner RA, Post K, Frisby K. Chemical model for short-term induction in quaking aspen (*Populus tremuloides*) foliage against herbivores. J Chem Ecol. 1989;15(9):2335–46. doi: 10.1007/BF01012085 24272421

[128] Strauss SY, Agrawal AA. The ecology and evolution of plant tolerance to herbivory. Trends Ecol Evol. 1999;14(5):179–85. doi: 10.1016/s0169-5347(98)01576-6 10322530

[129] Bernards MA, Båstrup-Spohr L. Phenylpropanoid metabolism induced by wounding and insect herbivory. In: Schaller A, editor. Induced plant resistance to herbivory. Dordrecht: Springer Netherlands; 2008. p. 189–211.

[130] Haukioja E, Neuvonen S. Induced long-term resistance of birch foliage against defoliators: defensive or incidental? Ecology. 1985;66(4):1303–8. doi: 10.2307/1939183

[131] Furstenburg D, van Hoven W. Condensed tannin as anti-defoliate agent against browsing by giraffe (*Giraffa camelopardalis*) in the Kruger National Park. Comparative Biochemistry and Physiology Part A: Physiology. 1994;107(2):425–31. doi: 10.1016/0300-9629(94)90402-2

[132] Metlen KL, Aschehoug ET, Callaway RM. Plant behavioural ecology: dynamic plasticity in secondary metabolites. Plant Cell Environ. 2009;32(6):641–53. doi: 10.1111/j.1365-3040.2008.01910.x 19021888

[133] Gutzeit HO, Ludwig-Müller J. Plant natural products: synthesis, biological functions and practical applications. Weinheim: John Wiley & Sons; 2014. 422 p.

[134] Schrijvers-Gonlag M, Skarpe C, Andreassen HP. Influence of light availability and soil productivity on insect herbivory on bilberry (*Vaccinium myrtillus* L.) leaves following mammalian herbivory. PLoS ONE. 2020;15(3):e0230509. doi: 10.1371/journal.pone.0230509 32218604 PMC7100976

[135] Jaakola L, Määttä-Riihinen K, Kärenlampi S, Hohtola A. Activation of flavonoid biosynthesis by solar radiation in bilberry (*Vaccinium myrtillus* L.) leaves. Planta. 2004;218(5):721–8. doi: 10.1007/s00425-003-1161-x 14666422

[136] Atlegrim O, Sjöberg K. Response of bilberry (*Vaccinium myrtillus*) to clear-cutting and single-tree selection harvests in uneven-aged boreal *Picea abies* forests. For Ecol Manage. 1996;86(1–3):39–50. doi: 10.1016/S0378-1127(96)03794-2

[137] Close DC, McArthur C. Rethinking the role of many plant phenolics—protection from photodamage not herbivores? Oikos. 2002;99(1):166–72. doi: 10.1034/j.1600-0706.2002.990117.x

[138] Mithöfer A, Riemann M, Faehn CA, Mrazova A, Jaakola L. Plant defense under Arctic light conditions: can plants withstand invading pests? Front Plant Sci. 2022;13. doi: 10.3389/fpls.2022.1051107 36507393 PMC9729949

[139] Emus-Medina A, Contreras-Angulo LA, Ambriz-Perez DL, Vazquez-Olivo G, Heredia JB. UV light stress induces phenolic compounds in plants. In: Lone R, Khan S, Al-Sadi AM, editors. Plant phenolics in abiotic stress management. Springer Nature Singapore; 2023. p. 415–440.

[140] Thitz P, Mehtätalo L, Välimäki P, Randriamanana T, Lännenpää M, Hagerman AE, et al. Phytochemical shift from condensed tannins to flavonoids in transgenic *Betula pendula* decreases consumption and growth but improves growth efficiency of *Epirrita autumnata* larvae. J Chem Ecol. 2019. doi: 10.1007/s10886-019-01134-9 31879865 PMC7056695

[141] War AR, Paulraj MG, Hussain B, Buhroo AA, Ignacimuthu S, Sharma HC. Effect of plant secondary metabolites on legume pod borer, *Helicoverpa armigera*. J Pest Sc. 2013;86(3):399–408. doi: 10.1007/s10340-013-0485-y

[142] Bernays EA, Chapman RF. Deterrent chemicals as a basis of oligophagy in *Locusta migratoria* (L.). Ecol Entomol. 1977;2(1):1–18. doi: 10.1111/j.1365-2311.1977.tb00861.x

[143] Leiss KA, Maltese F, Choi YH, Verpoorte R, Klinkhamer PGL. Identification of chlorogenic acid as a resistance factor for thrips in chrysanthemum. Plant Physiol. 2009;150(3):1567–75. doi: 10.1104/pp.109.138131 19448039 PMC2705022

[144] Mallikarjuna N, Kranthi KR, Jadhav DR, Kranthi S, Chandra S. Influence of foliar chemical compounds on the development of *Spodoptera litura* (Fab.) in interspecific derivatives of groundnut. J Appl Entomol. 2004;128(5):321–8. doi: 10.1111/j.1439-0418.2004.00834.x

[145] Ikonen A, Tahvanainen J, Roininen H. Phenolic secondary compounds as determinants of the host plant preferences of the leaf beetle, *Agelastica alni*. Chemoecol. 2002;12(3):125–31. doi: 10.1007/s00012-002-8337-2

[146] Matsuda K, Senbo S. Chlorogenic acid as a feeding deterrent for the Salicaceae-feeding leaf beetle, *Lochmaeae capreae cribrata* (Coleoptera: Chrysomelidae) and other species of leaf beetles. Appl Ent Zool. 1986;21(3):411–6. doi: 10.1303/aez.21.411

[147] Kundu A, Vadassery J. Chlorogenic acid-mediated chemical defence of plants against insect herbivores. Plant Biol. 2019;21(2):185–9. doi: 10.1111/plb.12947 30521134

[148] Laine KM, Henttonen H. Phenolics/nitrogen ratios in the blueberry *Vaccinium myrtillus* in relation to temperature and microtine density in Finnish Lapland. Oikos. 1987;50(3):389–95. doi: 10.2307/3565500

[149] Parzych A, Sobisz Z, Trojanowski J. Variability of nitrogen and phosphorus concentration and the net primary production of *Vaccinium vitis-idaea* L. and *Vaccinium myrtillus* L. in chosen woodland ecosystems of the Słowiński National Park. Archives of Environmental Protection. 2010;36(2):91–104.150.

[150] Selås V, Holand O, Ohlson M. Digestibility and N-concentration of bilberry shoots in relation to berry production and N-fertilization. Basic Appl Ecol. 2011;12(3):227–34. doi: 10.1016/j.baae.2011.01.004

[151] Ferwerda JG. Charting the quality of forage. Mapping and measuring the variation of chemical components in foliage with hyperspectral remote sensing. PhD-thesis: Wageningen Universiteit; 2005.

