# Supplementary material for: Phenolic concentrations and carbon/nitrogen ratio in annual shoots of bilberry (Vaccinium myrtillus) after simulated herbivory
Source: PLoS One. 2024 Mar 4;19(3):e0298229. doi: 10.1371/journal.pone.0298229 (PMC10911626; doi:10.1371/journal.pone.0298229)
Supplement: S1 File — (PDF) [file pone.0298229.s001.pdf]

Phenolic concentrations and carbon/nitrogen ratio in annual shoots of bilberry (*Vaccinium myrtillus*) after simulated herbivory.

Marcel Schrijvers-Gonlag, Christina Skarpe, Riitta Julkunen-Tiitto, Antonio B. S. Poléo

S1 Annual shoots and Random selection.

I. Annual shoots

Shoots are axes of the ramet which can carry leaves, buds and reproductive organs. Annual shoots are shoots grown in one growing season. Unless stated differently, we mean with 'annual shoots' the annual shoots from the current growing season, so in this study this is the growing season in the year 2014. Therefore, 'annual shoots from the previous year' are annual shoots grown in the growing season in 2013. We defined 'annual shoots' (from the current growing season) as end shoots (which do not yet contain fully grown shoots, so they are 'un-branched'), having either an intact end bud or other bud or one or more sprouting buds; clearly non-annual shoots that fulfill this working definition (for example brown, clearly old shoots with only one intact non-end bud present) were excluded.

When removing annual shoots by hand (treatment S, 'annual shoots cut') we removed the whole annual shoot from the ramet, including all tissue (as leaves and buds). From all randomly selected annual shoots used for analyses of total tannin concentration, identified phenolics and carbon and nitrogen, any remaining leaves attached to the shoot (often all leaves had fallen off already) were removed and excluded from the analyses.

II. Random selection

For random allocation of treatments, random selection of 10 % of annual shoots to be cut (treatment S10, 'annual shoots cut 10 %') and random selection of five annual shoots per ramet (if only five or less annual shoots were present at a ramet we took them all) we used a table with random numbers, made in Excel with the function 'RANDBETWEEN(1;x)' (where x is the maximum number), before we started field work. Leaves that were randomly chosen to be removed (treatment L10, 'leaves cut 10 %') were selected 'haphazardly', which means that we just picked the appropriate number of leaves from the ramet selecting quickly by eye (in a non-defined, chaotic manner), and every next leaf at least a bit away from the preceding one. Only when a plant was growing in a very dense way (high chance of damaging annual shoots when picking leaves haphazardly in the whole canopy) we chose as follows: we selected one leaf in the middle of one haphazardly selected annual shoot and thereafter haphazardly selected another annual shoot to remove one leaf from the middle section, and continued this procedure until the desired number of leaves was removed - all without, as far as possible, damaging the ramet (leaves and/or annual shoots). With treatment S10, 'annual shoots cut 10 %', we selected (using the mentioned table with random numbers) one annual shoot when 5-14 annual shoots were present, two annual shoots when 15-24 annual shoots were present, etcetera (similar with treatment L10, 'leaves cut 10 %': one leaf when 5-14 leaves were present, etcetera). With treatment S50 ('annual shoots cut 50 %') we selected every other annual shoot (similar with treatment L50, 'leaves cut 50 %': every other leaf).

Random selection of five dried ramets from each simulated herbivory treatment (including control) per location was more pragmatic: we took randomly stored dried ramets until five ramets per category were obtained.

To obtain balanced sample sizes in the analyses, samples remaining for analyses were selected in the R script with the command 'sample(), replace = FALSE'.
