# Supplementary material for: Phenolic concentrations and carbon/nitrogen ratio in annual shoots of bilberry (Vaccinium myrtillus) after simulated herbivory
Source: PLoS One. 2024 Mar 4;19(3):e0298229. doi: 10.1371/journal.pone.0298229 (PMC10911626; doi:10.1371/journal.pone.0298229)
Supplement: S2 File — (PDF) [file pone.0298229.s002.pdf]

Phenolic concentrations and carbon/nitrogen ratio in annual shoots of bilberry (*Vaccinium myrtillus*) after simulated herbivory.

Marcel Schrijvers-Gonlag, Christina Skarpe, Riitta Julkunen-Tiitto, Antonio B. S. Poléo

S2 Standard reference curve and Tannin color test.

I. Standard reference curve (tannin concentration ~ absorbance)

To relate tannin concentration in our subsamples to observed absorbance in a tannin color test (see below under II) we built a standard reference curve. First, we extracted tannins by adding approximately 50 ml acetone (70 %) (35.0 ml acetone (100 %) and 15.0 ml purified water (Milli-Q)) to approximately 75 mg pulverized shoots (from one ramet). This subsample was homogenized for 2 min at 24000 rpm with an Ultra-Turrax T25 homogenizer (Janke & Kunkel, IKA-Labortechnik, Germany), incubated in an ice bath (4 °C) for 30 min (dissolving time), homogenized again (2 min, 24000 rpm) and incubated on ice for 5 min (residue sedimentation). We filtered the subsample (using ashless filter paper and a water jet pump: Heto Lab Equipment, SUE30 Q, Denmark) and repeated the extraction procedure. To evaporate the acetone from the combined filtrates, we used a vacuum evaporator (40 °C; Laborota 4002 and ROTAVAC senso, Heidolph, Germany). The resulting tannin subsample was stored at 4-6 °C. Second, we used Sephadex LH-20 (GE Healthcare Bio-Sciences AB, Sweden) for tannin purification [1: S1, 2]. Approximately 2 g Sephadex was stabilized by adding 25.0 ml ethanol (80 %) for 30 min twice. After ethanol removal the Sephadex was allowed to set down overnight at 4-6 °C. Next, the tannin subsample was dissolved in 3.0 ml ethanol (80 %) and centrifuged (3 min, 3000 rpm, 20 °C; Eppendorf Centrifuge 5810 R; Eppendorf AG, Germany). The Sephadex was mixed gently (turned upside down) with 1.5 ml

of the dissolved tannin subsample (fixing tannins onto Sephadex) and then centrifuged again (3 min, 3000 rpm, 20 °C). After discarding the supernatant, 10.0 ml ethanol (80 %) was added to the Sephadex to dissolve non-tannin compounds (although this treatment also removed the most pro-oxidant ellagitannins [1]) and the subsample was mixed gently (turned upside down) and centrifuged again (3 min, 3000 rpm, 20 °C). The absorbance of the supernatant (after using filter paper to remove Sephadex particles) was measured with a spectrophotometer at wavelength 280 nm, using ethanol (80 %) as a blank (NanoDrop 2000; Thermo Fisher Scientific, USA). The last step (adding ethanol to the Sephadex) was repeated until measured absorbance of the supernatant was less than 0.060 absorbance units (AU). The Sephadex-bound tannins were released by adding 15.0 ml acetone (70 %), gently mixing (turning upside down) and centrifuging (3 min, 3000 rpm). This was done twice; the supernatants with tannins were combined and filtered (using double filter paper and the water jet pump to remove even the smallest Sephadex particles) into a 100 ml boiling bottle. After removal of the acetone with the vacuum evacuator we added 5.0 ml purified water (Milli-Q), froze the subsample using liquid nitrogen and stored it overnight at -23 °C. The subsample was dried using a freeze-dryer (Alpha 1-4 LDplus; Christ, Germany). Tthe dried tannins were weighted (result: 1.1748 mg tannins) and dissolved in 5.0 ml methanol (100 %). This solution was used to make different tannin concentrations by diluting with methanol (100 %). These were used to build a standard reference curve (tannin amount (µg tannins) ~ absorbance (AU) at wavelength 550 nm): Figure below; n = 13) following the procedure for a tannin color test for dissolved tannins as described below. The standard reference curve formula we obtained was:

tannin amount in µg = (absorbance in AU at wavelength 550 nm - 0.0300616) / 0.0034691

The adjusted R-squared value was 0.9807.

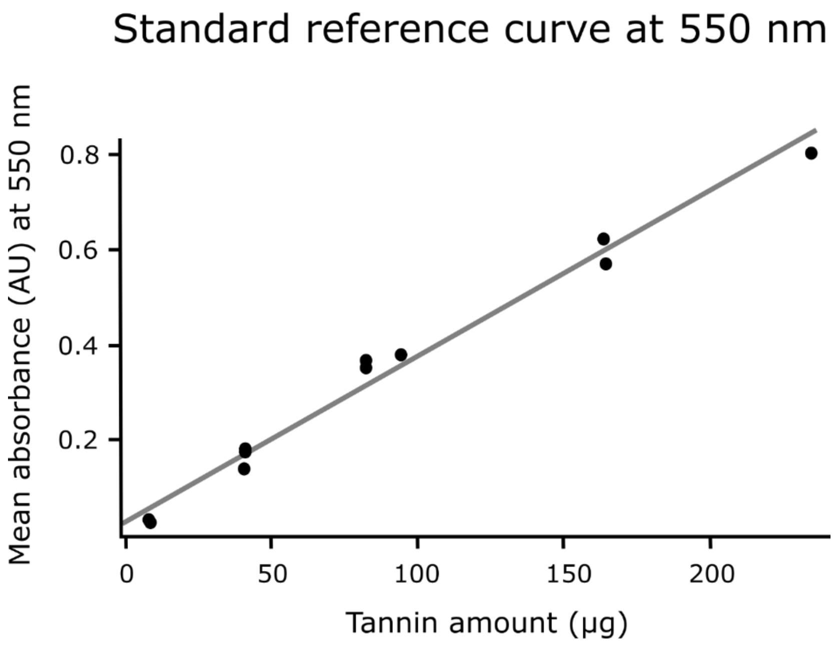

II. Tannin color test

We slightly adjusted the acid butanol assay for proanthocyanidins [3] to measure tannins in our subsamples. We balanced 0.5 – 0.8 mg pulverized shoots and added 6.0 ml acid butanol-reagent (1-butanol (100 %) - hydrochloric acid (37 %) 95/5), 0.25 ml iron reagent (2% Ferric ammonium sulfate in 2 N hydrochloric acid) and 1.00 ml methanol (100 %). After vortexing for 10 s on highest speed (Heidolph REAX 2000, West-Germany), the subsample was hydrolysed in a boiling water bath for 50 min, cooled down in ice water and centrifuged (3 min, 3000 rpm, 20 °C; Eppendorf Centrifuge 5810 R). Supernatant absorbance was measured three times for each subsample (we filled the vial/cube three times with a fresh part of the subsample and measured absorbance each time, or two times when these gave similar results) at wavelength 550 nm (Spectronic 20 Genesys; Spectronic Instruments, USA).

Note that more than 10 % (depending on tissue) of all condensed tannins can be present in an unextractable form (not extracted by usual solvents) [4-6]. Part of this fraction can be released by applying the acid butanol assay to the solid residue [7] (we have not done this in our study).

References

1. Salminen JP, Karonen M. Chemical ecology of tannins and other phenolics: we need a change in approach. Funct Ecol. 2011;25(2):325-38. doi: 10.1111/j.1365-2435.2010.01826.x.

2. Hagerman AE. Sephadex LH 20. In: Hagerman AE, editor. The tannin handbook. Miami University, Oxford. 2002. Accessed in 2021, URL: http://www.users.miamioh.edu/hagermae/2002.

3. Hagerman AE. Acid butanol assay for proanthocyanidins. In: Hagerman AE, editor. The tannin handbook. Miami University, Oxford. 2002. Accessed in 2021, URL: http://www.users.miamioh.edu/hagermae/2002.

4. Tarascou I, Souquet JM, Mazauric JP, Carrillo S, Coq S, Canon F, et al. The hidden face of food phenolic composition. Arch Biochem Biophys. 2010;501(1):16-22. doi: 10.1016/j.abb.2010.03.018.

5. Arranz S, Saura-Calixto F, Shaha S, Kroon PA. High contents of nonextractable polyphenols in fruits suggest that polyphenol contents of plant foods have been underestimated. J Agric Food Chem. 2009;57(16):7298-303. doi: 10.1021/jf9016652.

6. Hellström JK, Törrönen AR, Mattila PH. Proanthocyanidins in common food products of plant origin. J Agric Food Chem. 2009;57(17):7899-906. doi: 10.1021/jf901434d.

7. Barbehenn RV, Peter Constabel C. Tannins in plant–herbivore interactions. Phytochemistry. 2011;72(13):1551-65. doi: 10.1016/j.phytochem.2011.01.040.
