# Supplementary material for: Phenolic concentrations and carbon/nitrogen ratio in annual shoots of bilberry (Vaccinium myrtillus) after simulated herbivory
Source: PLoS One. 2024 Mar 4;19(3):e0298229. doi: 10.1371/journal.pone.0298229 (PMC10911626; doi:10.1371/journal.pone.0298229)
Supplement: S3 File — (PDF) [file pone.0298229.s003.pdf]

Phenolic concentrations and carbon/nitrogen ratio in annual shoots of bilberry (*Vaccinium myrtillus*) after simulated herbivory.

Marcel Schrijvers-Gonlag, Christina Skarpe, Riitta Julkunen-Tiitto, Antonio B. S. Poléo

S3 Quantifying phenolics using HPLC.

Quantification of methanol-soluble phenolics using high performance liquid chromatography (HPLC).

To extract phenolics, we added 600 µl methanol (100 %, 4 °C) to approximately 5 mg pulverized shoots and added three steal beads (2.8 mm). This subsample was homogenized for 25 s at 5500 rpm with a Precellys 24 homogenizer, incubated in an ice bath (4 °C) for 15 min and homogenized again (25 s, 5500 rpm). Then the mixture was centrifuged 3.0 min at 13.0 thousand rpm at 4 °C (Eppendorf Centrifuge 5418 R; Eppendorf AG, Germany). We separated the supernatant and repeated phenolic extraction from the subsample residue three times as described above, using 5 min incubation time in an ice bath instead of 15 min. We combined the four supernatants, removed methanol with an evaporator (vacuum centrifuge) 7-10 min at 45 °C (Eppendorf Concentrator plus) and stored the dry extract at -18 °C until further analysis. We used D(-)-Salicin min. 99 % CHR (Aldrich-Chemie, West-Germany) in methanol (100 %) as an internal standard in two out of five subsamples to evaluate extraction efficiency. To calculate the mean internal standard HPLC peak area (measured at 220 nm, retention time 2.2 min) we used eight HPLC runs on different days with the internal standard without bilberry tissue. Prior to HPLC analyses, we dissolved the dry extract in 300 µl methanol (100 %) and 300 µl purified water (Milli-Q, Millipak 0.22 µm, Merck Millipore, Germany). Phenolics were quantified using HPLC with injection volume 10 µl or 15 µl (only

two subsamples) (Agilent series 1100). We quantified compounds at wavelength 280 or 320 nm or, if necessary, at 220, 270 or 360 nm. We corrected the obtained values based on the Salicin-recovery percentage (internal standard), as follows. First, compound concentrations of subsamples run with an internal standard were converted to 100 % using the mean value of the internal standard HPLC peak area without bilberry tissue. Second, all subsample internal standard HPLC peak areas were averaged and with the mean value of the internal standard HPLC peak area without bilberry tissue we obtained the mean internal standard recovery rate (%), which was used to convert all compound concentrations of subsamples run without an internal standard to 100 %.

Phenolics were identified using a UHPLC quadrupole time-of-flight liquid chromatograph – mass spectrometer (Agilent Technologies, 6540 UHD Accurate-Mass Q-TOF LC/MS, 1290 Infinity) as described by Nissinen and colleagues [1].

Reference

1. Nissinen K, Virjamo V, Randriamanana T, Sobuj N, Sivadasan U, Mehtätalo L, et al. Responses of growth and leaf phenolics in European aspen (*Populus tremula*) to climate change during juvenile phase change. Can J For Res. 2017;47(10):1350-63. doi: 10.1139/cjfr-2017-0188.
