# Supplementary material for: Phenolic concentrations and carbon/nitrogen ratio in annual shoots of bilberry (Vaccinium myrtillus) after simulated herbivory
Source: PLoS One. 2024 Mar 4;19(3):e0298229. doi: 10.1371/journal.pone.0298229 (PMC10911626; doi:10.1371/journal.pone.0298229)
Supplement: S1 Table — (PDF) [file pone.0298229.s004.pdf]

Phenolic concentrations and carbon/nitrogen ratio in annual shoots of bilberry (*Vaccinium myrtillus*) after simulated herbivory.

Marcel Schrijvers-Gonlag, Christina Skarpe, Riitta Julkunen-Tiitto, Antonio B. S. Poléo

S4 Table: Response factors.

Response factors for phenolics at different wavelengths. Not for all phenolics are response factors available for all wavelengths. Response factors for arbutin derivatives are based on the response factor values for salidroside. Preferred wavelengths (280 nm or 320 nm) in **bold**.

	220 nm	270 nm	280 nm	320 nm	360 nm
**Flavonoids**					
Epicatechin	0.000486	0.00376	**0.0028**		
Gallocatechin derivative	0.000486	0.00376	**0.0028**		
Hyperin[1]	0.00087	0.00107	0.002194	**0.001598**	0.001029
Isorhamnetin 3-glucoside	0.00087	0.00107	0.002194	**0.001598**	0.001029
Kaempferol 3-glucoside[2]	0.00825	0.000898	0.001617	**0.001123**	0.001071
Monocoumaroyl-isoquercitrin[3,8]	0.00087	0.00107	0.002194	**0.001598**	0.001029
Procyanidin	0.000973	0.011493	**0.007865**		
Quercetin 3-arabinoside[4]	0.00087	0.00107	0.002194	**0.001598**	0.001029
Quercetin 3-glucuronide[5]	0.00087	0.00107	0.002194	**0.001598**	0.001029
Quercitrin[6]	0.00087	0.00107	0.002194	**0.001598**	0.001029
**Hydroquinones**					
Arbutin derivative[7]	0.0015166	0.0082526	**0.00802697**		
**Phenolic acids**					
Chlorogenic acid	0.001004	0.002552	0.001238	**0.000748**	0.003636
Cinnamic acid derivative	0.000578	0.000722	0.000425	**0.000294**	0.012101
Para-hydroxycinnamic acid derivative	0.000578	0.000722	0.000425	**0.000294**	0.012101
Protocatechuic acid derivative	0.0003314	0.0007722	**0.0013289**	0.0180992	

Some synonyms (PubChem, https://pubchem.ncbi.nlm.nih.gov/): [1]hyperoside, quercetin 3-galactoside; [2]astragalin(e); [3]isoquercitrin: quercetin 3-glucoside, isoquercetin, 3-glucosylquercetin, hirsutrin; [4]avicularine; [5]miquelianin, quercituron; [6]quercetrin, quercetin 3-rhamnoside; [7]aglucon of arbutin (arbutin: arbutine, arbutyne, arbutoside, ursin, uvasol, hydroquinone β-D-glucopyranoside, 4-hydroxyphenyl β-D-glucopyranoside). [8]Monocoumaroyl-isoquercitrin: identification uncertain.
