# Supplementary material for: Phenolic concentrations and carbon/nitrogen ratio in annual shoots of bilberry (Vaccinium myrtillus) after simulated herbivory
Source: PLoS One. 2024 Mar 4;19(3):e0298229. doi: 10.1371/journal.pone.0298229 (PMC10911626; doi:10.1371/journal.pone.0298229)
Supplement: S2 Table — (PDF) [file pone.0298229.s005.pdf]

Phenolic concentrations and carbon/nitrogen ratio in annual shoots of bilberry (*Vaccinium myrtillus*) after simulated herbivory.

Marcel Schrijvers-Gonlag, Christina Skarpe, Riitta Julkunen-Tiitto, Antonio B. S. Poléo

S5 Table: Phenolic concentrations after simulated herbivory.

Mean concentration (mg/g ± standard error; standard deviation given after a semicolon; dry weight) of 22 phenolics separately, and for all 22 phenolics together, in bilberry annual shoots. Number of observations given per treatment.

Minimum and maximum values per compound are indicated with a shaded background: minimum values lighter shaded than maximum values.

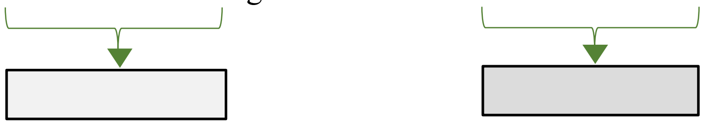

Footnotes in the table:

Some synonyms (PubChem, https://pubchem.ncbi.nlm.nih.gov/): [1]hyperoside, quercetin 3-galactoside; [2]astragalin(e); [3]isoquercitrin: quercetin 3-glucoside, isoquercetin, 3-glucosylquercetin, hirsutrin; [4]avicularine; [5]miquelianin, quercituron; [6]quercetrin, quercetin 3-rhamnoside; [7]aglucon of arbutin (arbutin: arbutine, arbutyne, arbutoside, ursin, uvasol, hydroquinone β-D-glucopyranoside, 4-hydroxyphenyl β-D-glucopyranoside).

[8]Monocoumaroyl-isoquercitrin: identification uncertain.

	Treatment								
	C (n = 33) (Control)	L10 (n = 32) (Leaves cut 10 %)	L50 (n = 32) (Leaves cut 50 %)	L100 (n = 32) (Leaves cut 100 %)	S10 (n = 25) (Annual shoots cut 10 %)	S50 (n = 25) (Annual shoots cut 50 %)	S100 (n = 26) (Annual shoots cut 100)%	R (n = 26) (Ramet cut)	All treatments (n = 231)
**Flavonoids**									
Epicatechin	9.81±0.55; 3.16	8.16±0.56; 3.16	8.75±0.56; 3.16	8.69±0.56; 3.16	9.65±0.63; 3.16	7.22±0.63; 3.16	6.80±0.62; 3.16	5.62±0.62; 3.16	8.17±0.22; 3.38
Gallocatechin derivative	0.90±0.08; 0.43	0.80±0.08; 0.43	0.78±0.08; 0.43	0.88±0.08; 0.43	0.99±0.09; 0.43	0.88±0.09; 0.43	0.80±0.08; 0.43	0.65±0.08; 0.43	0.83±0.03; 0.43
Hyperin[1]	3.38±0.40; 2.31	3.22±0.41; 2.31	3.75±0.41; 2.31	3.96±0.41; 2.31	2.96±0.46; 2.31	2.72±0.46; 2.31	1.99±0.45; 2.31	2.06±0.45; 2.31	3.07±0.16; 2.38
Isorhamnetin 3-glucoside	0.54±0.04; 0.23	0.48±0.04; 0.23	0.49±0.04; 0.23	0.54±0.04; 0.23	0.47±0.05; 0.23	0.49±0.05; 0.23	0.49±0.05; 0.23	0.41±0.05; 0.23	0.49±0.02; 0.23
Kaempferol 3-glucoside[2]	0.22±0.04; 0.25	0.25±0.04; 0.25	0.29±0.04; 0.25	0.20±0.04; 0.25	0.17±0.05; 0.25	0.13±0.05; 0.25	0.11±0.05; 0.25	0.17±0.05; 0.25	0.20±0.02; 0.25
Monocoumaroyl-isoquercitrin[3,8]	1.25±0.15; 0.84	1.24±0.15; 0.84	1.22±0.15; 0.84	0.85±0.15; 0.84	1.14±0.17; 0.84	1.53±0.17; 0.84	1.20±0.17; 0.84	1.43±0.17; 0.84	1.22±0.06; 0.85
Procyanidin 1	3.92±0.27; 1.53	4.36±0.27; 1.53	3.26±0.27; 1.53	3.46±0.27; 1.53	4.42±0.31; 1.53	3.49±0.31; 1.53	3.56±0.30; 1.53	2.99±0.30; 1.53	3.69±0.10; 1.58
Procyanidin 2	14.15±0.74;4.24	13.60±0.75;4.24	13.20±0.75;4.24	12.76±0.75;4.24	14.86±0.85;4.24	11.01±0.85;4.24	10.60±0.83;4.24	8.95±0.83; 4.24	12.50±0.30;4.55
Procyanidin 3	17.67±1.33;7.67	20.40±1.36;7.67	15.65±1.36;7.67	16.28±1.36;7.67	19.03±1.53;7.67	15.57±1.53;7.67	15.66±1.50;7.67	15.37±1.50;7.67	17.01±0.51;7.76
Procyanidin 4	14.03±0.89;5.13	13.81±0.91;5.13	13.70±0.91;5.13	13.20±0.91;5.13	15.36±1.03;5.13	10.97±1.03;5.13	10.94±1.01;5.13	8.45±1.01; 5.13	12.68±0.36;5.44
Procyanidin 5	10.44±0.72;4.13	10.32±0.73;4.13	10.59±0.73;4.13	9.39±0.73; 4.13	11.45±0.83;4.13	8.39±0.83; 4.13	7.90±0.81; 4.13	6.21±0.81; 4.13	9.42±0.29; 4.36
Procyanidin 6	3.95±0.46; 2.62	4.42±0.46; 2.62	4.03±0.46; 2.62	4.69±0.46; 2.62	4.23±0.52; 2.62	4.46±0.52; 2.62	3.34±0.51; 2.62	3.45±0.51; 2.62	4.09±0.17; 2.62
Quercetin 3-arabinoside[4]	0.63±0.10; 0.56	0.54±0.10; 0.56	0.71±0.10; 0.56	0.76±0.10; 0.56	0.60±0.11; 0.56	0.46±0.11; 0.56	0.27±0.11; 0.56	0.22±0.11; 0.56	0.54±0.04; 0.58
Quercetin 3-glucuronide[5]	9.80±0.72; 4.13	9.03±0.73; 4.13	9.10±0.73; 4.13	9.85±0.73; 4.13	8.35±0.83; 4.13	6.64±0.83; 4.13	6.34±0.81; 4.13	5.32±0.81; 4.13	8.21±0.29; 4.37
Quercitrin[6]	0.91±0.11; 0.64	0.68±0.11; 0.64	0.89±0.11; 0.64	0.86±0.11; 0.64	0.82±0.13; 0.64	0.62±0.13; 0.64	0.59±0.13; 0.64	0.44±0.13; 0.64	0.74±0.04; 0.65
**Hydroquinones**									
Arbutin derivative[7]	2.67±0.15; 0.87	2.56±0.15; 0.87	2.80±0.15; 0.87	2.79±0.15; 0.87	2.75±0.17; 0.87	2.72±0.17; 0.87	2.48±0.17; 0.87	1.71±0.17; 0.87	2.57±0.06; 0.91
**Phenolic acids**									
Chlorogenic acid	5.24±0.36; 2.04	5.37±0.36; 2.04	5.47±0.36; 2.04	5.70±0.36; 2.04	6.07±0.41; 2.04	5.16±0.41; 2.04	5.56±0.40; 2.04	5.90±0.40; 2.04	5.54±0.13; 2.03
Cinnamic acid derivative	1.16±0.12; 0.70	1.15±0.12; 0.70	1.20±0.12; 0.70	1.15±0.12; 0.70	1.10±0.14; 0.70	1.25±0.14; 0.70	0.88±0.14; 0.70	1.14±0.14; 0.70	1.13±0.05; 0.70
Para-hydroxycinnamic acid der. 1	1.24±0.10; 0.04	1.31±0.10; 0.04	1.03±0.10; 0.04	1.00±0.10; 0.04	1.26±0.11; 0.04	1.08±0.11; 0.04	1.28±0.11; 0.04	1.15±0.11; 0.04	1.17±0.04; 0.06
Para-hydroxycinnamic acid der. 2	0.54±0.03; 0.17	0.53±0.03; 0.17	0.48±0.03; 0.17	0.52±0.03; 0.17	0.52±0.03; 0.17	0.46±0.03; 0.17	0.47±0.03; 0.17	0.41±0.03; 0.17	0.49±0.01; 0.18
Para-hydroxycinnamic acid der. 3	3.75±0.21; 1.20	3.74±0.21; 1.20	3.30±0.21; 1.20	3.71±0.21; 1.20	3.89±0.24; 1.20	3.22±0.24; 1.20	3.52±0.23; 1.20	2.94±0.23; 1.20	3.52±0.08; 1.22
Protocatechuic acid derivative	0.16±0.01; 0.06	0.16±0.01; 0.06	0.17±0.01; 0.06	0.17±0.01; 0.06	0.16±0.01; 0.06	0.13±0.01; 0.06	0.14±0.01; 0.06	0.12±0.01; 0.06	0.15±0.00; 0.06
**All phenolics**									
All 22 phenolics together	106.36±4.1;23.7	106.12±4.2;23.7	100.87±4.2;23.7	101.43±4.2;23.7	110.25±4.7;23.7	88.62±4.7; 23.7	84.92±4.6; 23.7	75.11±4.6;23.7	97.45±1.7; 25.9
