# Supplementary material for: Phenolic concentrations and carbon/nitrogen ratio in annual shoots of bilberry (Vaccinium myrtillus) after simulated herbivory
Source: PLoS One. 2024 Mar 4;19(3):e0298229. doi: 10.1371/journal.pone.0298229 (PMC10911626; doi:10.1371/journal.pone.0298229)
Supplement: Attachment — Submitted filename: Manuscript - MSG20230306 TRACK CHANGES VERSION.docx [file pone.0298229.s007.docx]

**Phenolic concentrations and carbon/nitrogen ratio in annual shoots of bilberry (*Vaccinium myrtillus*) after simulated herbivory.**

Marcel Schrijvers-Gonlag^1*^, Christina Skarpe^1^, Riitta Julkunen-Tiitto^2^, Antonio B. S. Poléo^1^

^1^ Campus Evenstad, Faculty of Applied Ecology, Agricultural Sciences and Biotechnology, Inland Norway University of Applied Sciences, Koppang, Norway

^2^ Department of Environmental and Biological Sciences, Faculty of Science, Forestry and Technology, University of Eastern Finland, Joensuu, Finland

* Corresponding author

Email: marcel.schrijversgonlag@inn.no (MSG)

**Abstract**

Herbivory can be reduced by the production of defense compounds (secondary metabolites) but generally defenses are costly and growth is prioritized over defense. While defense compounds may deter herbivory, elevated nutrients may promote it. In a field study in the boreal forest of Norway, we investigated how simulated herbivory affected phenolic concentrations and the carbon/nitrogen (C/N) ratio in the annual shoots of bilberry (*Vaccinium myrtillus*), a deciduous clonal dwarf shrub whose vegetative parts and fruitprovide forage for many boreal forest animals. We measured concentrations of total tannins, individual phenolics, nitrogen and carbon following different types and intensities of simulated herbivory. Our results are presented in the context of several theories predicting plant resource allocation to secondary compounds: the Optimal Defense hypothesis, the Carbon:Nutrient Balance hypothesis and the Protein Competition Model. We identified 22 phenolics in bilberry annual shoots: 15 flavonoids, 1 hydroquinone and 6 phenolic acids. After high levels of simulated herbivory, the concentrations of total tannins total phenolics and phenolics synthesized in direct competition with protein, were significantly lower in bilberry annual shoots than under natural herbivory at low to intermediate levels (ambient herbivory). Less intensive simulated herbivory, including severe defoliation, gave no significantly different total tannin or total phenolic concentration compared with natural herbivory at low to intermediate levels (ambient herbivory). Many individual phenolics followed this pattern, while phenolic acids showed little response to the treatments. Contrary to our predictions, we found no significant difference in C/N ratio among treatments. We argue that this arises due to clonality: in clonal bilberry, compounds may be translocated from source ramets to ramets under simulated herbivory pressure to compensate for a lack of carbohydrates, nutrients or even phenolics.

**Introduction**

Bilberry (*Vaccinium myrtillus* L.) is an abundant plant species in boreal forests of Fennoscandia [1-3], andis a key understory component influencing the soils, succession and regeneration of forests in this region [4]. This deciduous clonal dwarf shrub has evergreen shoots and grows on nutrient-poor soil largely through the formation of ramets [1, 2, 5-8].

Vegetative and generative parts of bilberry are important forage for many mammal, bird and insect species in all seasons [9-16]. One of many strategiesto minimize herbivory on vegetative plant tissue, is the production of defense compounds ( secondary metabolites)[17, 18]. Under resourcelimitation, trade-offs occur among growth, maintenance, storage, reproduction and defense in plants [19, and references herein]. Of the theories that predict plant resource allocation to secondary compounds Ssome are especially relevant for bilberry-herbivore interactions.

The Optimal Defense hypothesis predicts that because antiherbivore chemicals are costly, production of inducible defenses should be low when herbivory is absent or nearly absent and should increase when such disturbances are great[20-28]. In general, however, growth is prioritized over defense [19, 29-32, but see 33, and references herein]. Therefore, a severe loss of photosynthetic tissue may not allow for production of defense compounds and may even lead to the breakdown of existing defenses, resulting in lower resistance to herbivory [34-37].

As many deciduous woody species growing on nutrient-poor soils, bilberry stores carbon in roots and other woody tissue, like stems [19, 30, 38, 39]. In the growing season, most nutrients are found within the foliage of deciduous species [19]. The Carbon:Nutrient Balance (CNB) hypothesis predicts a mobilization of these carbon reserves and an increase in the level of carbon-based defense compounds in bilberry after herbivory on shoots and leaves [19, 24, 38, 40]. Phenolic compounds (phenolics) are primarily composed of carbon [41] and can act as such carbon-based defense compounds reducing herbivore performance and herbivory [42-47]. Phenolics include tannins (condensed tannins or proanthocyanidins and hydrolyzable tannins), flavonoids and other small molecular mass phenolics, including cinnamic acids [41, 42, 48]. Many different phenolics have been identified in bilberry stems, shoots, leaves, berries and rhizomes [49-55].

Not all phenolics respond to herbivory as predicted by the CNB hypothesis, as many phenolics compete with proteins for the nitrogen containing precursor phenylalanine as described by Jones & Hartley [56] in their Protein Competition Model (PCM) [42, 57, 58]. As the boreal forest is a nitrogen-limited ecosystem [59-62], competition for nitrogen between biosynthesis of proteins and of many phenolics is expected in boreal forest ecosystems. However, hydrolyzable tannins are phenolics that have gallic acid as a precursor, which is partly synthesized via an intermediate of the shikimate pathway which does not involve nitrogen [42, 57, 63]. Consequently, hydrolyzable tannins are synthesized partly without direct competition with protein synthesis and thus likely, at least in part, to follow the CNB hypothesis after herbivory, while phenolics that are synthesized in direct competition with protein synthesis, i.e., phenylpropanoids and derived compounds such as (hydroxy)cinnamic acids, condensed tannins and other flavonoids, are not [42, 48, 55, 57, 64]. Therefore, we expect that the effects of tissue damage, resulting from herbivory or other causes, on phenolic concentration in bilberry vary depending on several factors. The damage type, whether leaves, shoots or the whole ramet is damaged, is one factor. Other factors are damage intensity, nitrogen availability, whether phenylalanine is a precursor in the synthesis of the particular phenolic, and the level the actual phenolic can function as an antiherbivore compound, as different phenolics have different biological functions and efficacy [65-71].

Defense compounds may deter herbivory, while elevated nutrients often associated with compensatory growth, may promote it [18, 72-76]. Nutrient concentration in bilberry is affected by habitat productivity and plant losses, among them losses by herbivory. Nitrogen concentration in bilberry, which is often used as a proxy for nutrient concentration, is positively related to habitat productivity [55, 77, 78]. Nitrogen concentration increases after browsing in several woody species, often regardless of soil productivity [79-83]. Pruning, the partial or complete removal of stem and/or shoots, reduces bud numbers and increases the root:shoot ratio. This results in decreased competition for nutrients among meristems, causing an increase in new plant tissue nutrient concentration [32, 84-88]. On the other hand, severe defoliation results in a loss of nitrogen [89], or at least in the loss of proportionally more nutrients than carbon [19], and in increased fine root mortality [90, 91]. This leads to reduced nutrient absorption which results in a decreased nutrient concentration, especially on nutrient-poor soils [92, 93]. Therefore, we expect that the effects of tissue damage, from herbivory or other causes, on nitrogen concentration in bilberry vary, depending on type and intensity of damage, and nitrogen availability in the soil.

Most research on phenolics in bilberry has focused on berries, although studies on leaves, shoots and stems have been conducted [49, 50, 54, 94-96]. Previous studies of herbivory, nutritional quality and defense responses of bilberry shoots and leaves did not involve controlled clipping experiments, nor measurements of phenolic, nitrogen and carbon concentrations [97-102]. For these reasons, and because we expected a measurable response in the young tissues of annual shoots after herbivory [20, 39, 103], we investigated how simulated herbivory affects phenolic concentrations and the carbon/nitrogen (C/N) ratio, often used as indicator of plant nutritional quality [104, 105] in bilberry annual shoots. We measured total tannin, individual phenolic, nitrogen and carbon concentrations in bilberry annual shoots after different types and intensities of simulated herbivory. Persson and colleagues [55] performed a simulated browsing study on bilberry investigating responses in phenolic and nitrogen concentrations and C/N ratio in leaves and leafless shoots. Different from Persson and colleagues, who performed different levels of simulated moose (*Alces alces* L.) browsing only, we used three types of simulated herbivory, representing herbivory by large ungulates (eating ramets), herbivory by smaller mammals, birds and insects (eating annual shoots) and herbivory by insects (eating leaves). Our study was performed under ambient herbivory conditions at three boreal forest locations in southeastern Norway in 2014.

Our objective was to examine how different herbivory types (ramet herbivory, annual shoot herbivory, leaf herbivory) and intensities affect the concentration of phenolic compounds and nitrogen (nutritional quality) in bilberry annual shoots and to consider our results in the context of the plant defense theories described above. Consequently, we predict that in in bilberry annual shoots: I. at low to intermediate (simulated) herbivory levels, phenolics synthesized without direct competition with protein synthesis are, , positively correlated with simulated herbivory;

II. phenolics synthesized in direct competition with protein synthesis is, at low to intermediate (simulated) herbivory levels, not correlated with simulated herbivory;

III. phenolics is, at high simulated herbivory levels, lower than without simulated herbivory;

IV. nitrogen is, at low to intermediate (simulated) herbivory levels, positively correlated with simulated herbivory, i.e., the C/N ratio is negatively correlated with simulated herbivory;

V., at high simulated herbivory levels, nitrogen is lower, i.e., the C/N ratio is higher, than without simulated herbivory, especially in bilberry growing on relatively nutrient-poor soil.

**Methods**

**Study area**

We conducted the study at three locations on mountain sides in the Østerdalen valley in southeastern Norway (Fig 1), in 2014. All locations were within 400-670 m.a.s.l and consisted of coniferous boreal forest. In 2014, mean annual temperature was 4.8 °C and total precipitation was 896 mm (Fig 1) [106]. At each location we selected bilberry plants at two sites: one with relatively low and one with relatively high soil productivity (value 8 and 14, respectively, in the H40 system for estimating forest productivity [107]). No permits for field site access were necessary, and permission for the experiment was given by the land owner.

**Fig 1. Map of the study area in southeastern Norway. A** Map of northern Europe. Inset: the study area. **B** Study area. Black thick line: main road (rv3); grey dashed line: the Glomma River; dashed ellipses: locations; open squares: low productivity sites; filled squares: high productivity sites. **C** Mean monthly temperature (°C) and total monthly precipitation (mm) in 2014 was measured at Evenstad weather station (latitude 61.4255, longitude 11.0803, 257 m.a.s.l) and Rena Airport weather station (latitude 61.1847, longitude 11.3747, 255 m.a.s.l), respectively.

**Study design**

Field treatments

At each site, we used four parallel transects spaced ten m apart, to select bilberry ramets with approximately two m distance between consecutive ramets. Along each line, we selected 33 or 34 ramets at the beginning of the growing season (May) and marked them with steel wire. Selected ramets had at least ten shoots longer than 1.0 cm from the previous growing season (annual shoots from 2013, S1 Supporting information), and no visual signs of extensive herbivory (most ramets had some past herbivory signs), so the initial herbivory level for all ramets was low. In total we selected 135 ramets at each site. We divided the ramets within each site randomly (S1 Supporting information) into four treatment groups: control (n = 30), 'leaves cut' (n = 45), 'annual shoots cut' (n = 45) and 'ramet cut' (n = 15). These treatments represented one group with ambient, initially low, herbivory only (the control group) and three groups with additional simulated herbivory: by insects (group 'leaves cut'), by insects and small-sized vertebrates (group 'annual shoots cut'), and by large ungulates (group 'ramet cut'). At all locations and sites, we removed leaves by hand ('leaves cut') at three different intensities: 10 % from 15 ramets, 50 % from another 15 ramets and finally 100 % from the remaining 15 ramets (S1 Supporting information). At all locations and sites, except location 'Evenstadlia', site 'low productivity', we removed annual shoots by hand ('annual shoots cut') at the same three intensities (10 %, 50 % and 100 %; n = 15 for each), and we cut the ramet in the 'ramet cut' treatment group by removing 90 % of the ramet with garden scissors. In total this resulted in eight treatments: 'control' (ambient, initially low, herbivory), 'leaves cut' (3 intensities), 'annual shoots cut' (3 intensities), 'ramet cut'. Ramets in the control treatment that experienced severe herbivory between selecting and harvesting, were excluded from our analyses: therefore, all control ramets experienced herbivory at low to intermediate levels (ambient herbivory). The shoots were removed and ramets cut 24 – 27 May and leaves were removed in the period 21 June to 2 July. In our experiment, we considered the control treatment as herbivory at the lowest level. Within the leaf removal treatments, 10 % leaf removal represented leaf herbivory at a low level, 50 % leaf removal represented leaf herbivory at an intermediate level and 100 % leaf removal represented leaf herbivory at a high level. Within the annual shoot removal treatments, 10 % annual shoot removal represented annual shoot herbivory at a low level, 50 % annual shoot removal represented annual shoot herbivory at an intermediate level and 100 % annual shoot removal represented annual shoot herbivory at a high level. We considered the treatment 'ramet cut' as herbivory at the highest level and the 100 % annual shoot removal treatment as herbivory at the second highest level in our experiment: these two treatments were the two most severe simulated herbivory treatments in our study.

Soil sampling

At each site we collected four soil samples from the upper organic layer down to maximum 10 cm depth, with a metal bulb planter, on 12-13 June. These four soil samples were merged to obtain one sample per site. We stored the soil samples in a freezer at -18 °C prior to analyses of nitrogen, as a proxy for soil productivity.

Bilberry sampling

All ramets (n = 750) were harvested towards the end of the growing season (19 – 28 August) by cutting the stem at ground level with garden scissors. The ramets were dried for minimum 48 hours in a drying oven (Binder FED 720 E2, Germany) at 30 °C, before the dried ramets were stored in a dark and dry place at room temperature. From each site, we randomly selected five dried ramets from each simulated herbivory treatment (including control), resulting in 40 ramets per site (20 for location 'Evenstadlia', site 'low productivity') and 220 ramets in total. From each of these ramets, we randomly selected five annual shoots (S1 Supporting information), continued drying these annual shoots for minimum 24 hours at 30 °C and stored them in a dark and dry place at room temperature, prior to preparation and analyses of total tannin concentration, individual phenolics concentration and carbon and nitrogen concentration.

**Chemical analyses**

Soil nitrogen

All soil samples were analyzed for total nitrogen (% dry matter; measuring method uncertainty ± 10 %; method reference EN 15104:2011/EN 15407:2011) (Eurofins Food & Agro Testing Sweden AB, Kristianstad/Linköping, April 2015). We did not use inorganic ammonium (NH_4_^+^) or nitrate (NO_3_^-^) concentrations (S2 Supporting information) in our analyses, as organic nitrogen is an important source of nitrogen to bilberry [108, 109].

Bilberry shoots

Before analyses of acetone-soluble tannins, methanol-soluble phenolics, and total carbon and nitrogen, the shoots were cut in fragments of maximum 0.5 cm and for each ramet we transferred these subsamples to a 2 ml or 7 ml vial with three stainless steel beads (2.8 mm) to pulverize the tissue; with large subsamples (approximately 200, 300 and 400 mg; all weight measurements in this study: scale Sartorius SE2, d=0.1 μg) we used four, five or six beads, respectively. The shoot fragments were pulverized by the beads using a Precellys 24 homogenizer (Bertin Technologies, France): 25 s at 5500 rpm, 15 times with two minutes in between. When handling the shoots, we used disposable latex gloves.

Shoot tannins

The shoots were analysed for acetone (70 %)-soluble tannins (e.g., hydrolyzable tannins and polymeric condensed tannins (proanthocyanidins)) [110, 111: S1, 112] with a spectrophotometer (Spectronic 20 Genesys; Spectronic Instruments, USA). To relate tannin concentration in our subsamples to measured absorbance (at 550 nm) we built a standard reference curve, using Sephadex LH-20 (GE Healthcare Bio-Sciences AB, Sweden) for tannin purification [111: S1, 113]. We slightly adjusted the acid butanol assay for proanthocyanidins [114] to measure tannins in our subsamples (S3 Supporting information).

Shoot phenolics

Methanol-soluble phenolics (e.g., flavonoids and phenolic acids) were extracted from the shoots and quantified using high performance liquid chromatography (HPLC) with injection volume 10 μl (Agilent series 1100) and identified using a UHPLC quadrupole time-of-flight liquid chromatograph – mass spectrometer (Agilent Technologies, 6540 UHD Accurate-Mass Q-TOF LC/MS, 1290 Infinity) as described by Nissinen and colleagues [115] and Hagerman [116] (S4 Supporting information). Individual compounds that could not be identified were not used in this study. We used D(-)-Salicin min. 99 % CHR (Aldrich-Chemie, West-Germany) in methanol (100 %) as an internal standard in two out of five subsamples to evaluate extraction efficiency (S4 Supporting information).

Shoot carbon and nitrogen

The shoots were analysed for carbon and nitrogen (total concentration (mg/g, dry weight) after destruction; micro CN-analyzer (thermo), Chemical Biological Soil Laboratory (quality system based on the ISO-17025 standard), Wageningen University, July 2016).

**Statistical analyses**

The total tannin absorbance measurements were averaged per subsample and with the standard reference curve and subsample weight these subsample means were converted to concentrations (mg tannins/g shoots, dry weight), which were used in further analyses. To investigate whether location and soil productivity site affected the response variable tannin concentration, we used a Kruskal-Wallis rank sum test and Dunn's non-parametric all-pairs rank comparison test with Holm correction to compare, for each treatment separately, the tannin concentration of these six location-soil groups (three locations, two soil productivity sites at each location; Fig 1). When location-soil groups were not significantly different (we used a significance level of 5% in all our analyses in this study) we merged the location data and site data to increase the sample size for each treatment and used the merged data in the analyses of both treatment – tannin concentration and treatment – individual phenolic concentration. Similarly, to investigate whether location and soil productivity site affected the response variable C/N ratio, we performed one-way ANOVA or Kruskal-Wallis tests with C/N ratio as response variable. As before, when location-soil groups showed no difference we merged, per treatment, these location-soil groups to increase sample size and used the merged data in the analyses of treatment – C/N ratio.

In our HPLC analyses we used the individual phenolic compound concentration (mg/g, dry weight) as the response variable in our modeling, calculated as: ( (rf x area) / weight ) / (inj / tot ) where rf is the HPLC response factor for the actual compound at the used wavelength, area is the peak area in the HPLC result table (mAU*s) at the used wavelength, weight is the initial shoot subsample weight (mg), inj is the HPLC injection volume (10 or 15 μl) and tot is the total volume (600 μl) in which the subsample was dissolved (300 μl methanol + 300 μl purified water, S4 Supporting information). The HPLC response factor is the ratio between the concentration of a specific compound (mg/g) and the response of the detector (area: mAU*s) to this compound at a specific wavelength; we used response factors previously determined using standards with known concentrations (S5 Supporting information). Before analyses, compound concentrations were converted to 100% to recover losses in the extraction procedure. First, compound concentrations of subsamples run with an internal standard were converted to 100 % using the mean value of the internal standard HPLC area without bilberry tissue. Second, all subsample internal standard HPLC peak areas were averaged and with the mean value of the internal standard HPLC area without bilberry tissue we obtained the mean internal standard recovery rate (%), which was used to convert all compound concentrations of subsamples run without an internal standard to 100 %. When no value in the HPLC result table was present for a compound, we used a concentration of 0 mg/g, although often a small peak on the HPLC chromatogram was visible.

Differences between treatments were investigated with a one-way ANOVA test or a Kruskal-Wallis rank sum test. In all ANOVA analyses in this study we used equal sample sizes across groups, to avoid inflation of error rates and to guarantee homogeneity of variance [117]. If necessary, samples were randomly removed to obtain balanced sample sizes. If sample size before or after balancing was less than the number of groups that were compared plus one we conducted a Kruskal-Wallis test (without balancing sample sizes) instead of an ANOVA test. We used the total tannin concentration (prediction III), the concentration of each phenolic compound (prediction I-III) and the concentration of all phenolic compounds together (prediction III) as response variable. We also used a one-way ANOVA test and a Kruskal-Wallis test to investigate differences between treatments on the response variable C/N ratio (predictions IV, V). When the ANOVA test or Kruskal-Wallis test indicated a significant difference, differences between groups were investigated with Tukey's HSD post-hoc test and Dunn's non-parametric all-pairs rank comparison test with Holm correction, respectively. For the Kruskal-Wallis and Dunn's tests we used the package 'PMCMRplus' in the software 'R' [118]. We used the package 'emmeans' to calculate some general statistics and to further investigate the relationship between several response variables and treatments [119]. Figures were created with the package 'svglite' [120] and the software 'Inkscape' (version 1.2.1). All model analyses were performed in R, version 4.1.2. and 4.2.2. [121].

**Results**

Soil nitrogen and sample size

In total we analysed 232 ramets. Soil total nitrogen concentration was not very different between locations and sites, except for location 'Vinjevegen', site 'low productivity' which showed a much lower total nitrogen concentration (Table 1). Kruskal-Wallis analyses of both the tannin concentration and the C/N ratio of the six location-soil groups (three locations, two soil productivity sites at each location; Fig 1) supported this difference, but only for three treatments (tannin concentration, control treatment: Kruskal-Wallis test coefficient χ^2^ = 12.2, d.f. = 5, *P* = 0.032; tannin concentration, treatment 'all annual shoots cut': χ^2^ = 13.0, d.f. = 4, *P* = 0.011; C/N ratio, treatment 'ramet cut': χ^2^ = 12.4, d.f. = 4, *P* = 0.014; all other Kruskal-Wallis analyses gave no significant result). In all three cases, location 'Vinjevegen', site 'low productivity' differed significantly from location 'Evenstadlia', site 'high productivity'. Therefore, we merged all locations and sites except location 'Vinjevegen', site 'low productivity' to increase sample size. All remaining analyses on differences between treatments in tannin concentration, individual phenolic concentration and C/N ratio were done with both the merged dataset (called 'data-merged', n = 192) and with the dataset from location 'Vinjevegen', site 'low productivity' only (called 'Vin-low', n = 40). Analyses not concerning treatments were done with the complete dataset (called 'all data', n = 232).

**Table 1. Soil nitrogen in the study area.** Total nitrogen concentration (% dry matter) per location (a mountain side in the Østerdalen valley) and site (relatively low and high soil productivity according to the H40 system) (n = 1).

| **Location** | **Soil productivity** | **Soil nitrogen** |
| --- | --- | --- |
| Evenstadlia | low | 1.08 |
| Evenstadlia | high | 1.17 |
| Imsdalen | low | 1.34 |
| Imsdalen | high | 1.24 |
| Vinjevegen | low | 0.19 |
| Vinjevegen | high | 1.01 |

Shoot tannins

Tannin concentration (mg/g, dry weight) varied between ramets and on average bilberry annual shoots consisted of circa 25-30 % tannins (dry weight) (all data: min = 115.2, mean = 270.5, max = 415.4, sd (standard deviation of the sample) = 44.1, se (standard error of the sample mean) = 2.9 (n = 232)). Almost all treatments resulted in lower mean tannin concentrations than the control, only the treatment 'annual shoots cut 10 %' resulted in a higher mean tannin concentration than the control in data-merged (Table 2). The most severe simulated herbivory treatment (90 % ramet removal) resulted in the lowest mean tannin concentrations in both data-merged and Vin-low (Table 2).

**Table 2. Tannin concentration in bilberry annual shoots after simulated herbivory.** Mean concentration (mg/g ± standard error, dry weight) for the merged dataset ('data-merged', n = 192) and for data from location 'Vinjevegen', site 'low productivity' ('Vin-low', n = 40).

| **Treatment** | **Data-merged (n)** | **Vin-low (n)** |
| --- | --- | --- |
| Control | 285.7 ± 7.4 (28) | 321.1 ± 16.9 (5) |
| Leaves cut 10 % | 271.4 ± 7.4 (28) | 316.1 ± 16.9 (5) |
| Leaves cut 50 % | 271.3 ± 7.5 (27) | 299.5 ± 16.9 (5) |
| Leaves cut 100 % | 269.8 ± 7.5 (27) | 299.3 ± 16.9 (5) |
| Annual shoots cut 10 % | 293.8 ± 8.8 (20) | 288.5 ± 16.9 (5) |
| Annual shoots cut 50 % | 253.2 ± 8.8 (20) | 272.9 ± 16.9 (5) |
| Annual shoots cut 100 % | 236.3 ± 8.5 (21) | 303.8 ± 16.9 (5) |
| Ramet cut | 229.7 ± 8.5 (21) | 260.6 ± 16.9 (5) |
| All treatments together | 265.3 ± 3.1 (192) | 295.2 ± 6.2 (40) |

We found a significant difference in tannin concentration between several treatments in data-merged (ANOVA: F_7,152_ = 7.13, *P* < 0.001) but differences between treatments were not significant in Vin-low (Kruskal-Wallis: χ^2^ = 11.5, d.f. = 7, *P* = 0.12) (Fig 2). In data-merged, all leaf removal treatments did not differ significantly from each other nor from the control. In this dataset, simulated annual shoot herbivory at a low level resulted in a higher tannin concentration than simulated annual shoot herbivory at intermediate or high levels. Simulated herbivory at the highest level ('ramet cut') resulted in a lower tannin concentration than the lowest herbivory level (the control) in data-merged (Fig 2).

**Fig 2. Tannin concentration in bilberry annual shoots after simulated herbivory**. Boxplot with total concentration (mg/g, dry weight) for the merged dataset ('data-merged', n = 160, every treatment n = 20, dark grey boxes) to the left and data from location 'Vinjevegen', site 'low productivity' ('Vin-low', n = 40, every treatment n = 5, light grey boxes) to the right. The bottom and top of each box indicate the first and third quartiles. Bold horizontal lines within each box indicate median values. The plot whiskers extend to the most extreme data point which is no more than 1.5 times the interquartile range away from the box; extreme data points more than 1.5 times the interquartile range away from the box are indicated with black points. Treatments with the same letter above the box are not different from each other (*P* > 0.05). ns = no significant difference between any of the treatments.

Shoot phenolics

We identified 22 phenolic compounds: 15 flavonoids, 1 hydroquinone and 6 phenolic acids (Fig 3). The recovery of the internal standard was around 95 %: min = 58.9 %, mean = 94.5 %, max = 107.5 %, sd = 6.1 % (n = 90). In one subsample the recovery of the internal standard was quite different from all others (58.9 %). Therefore, we excluded this subsample from the phenolic analyses. Without this subsample, the recovery of the internal standard changed to: min = 83.1 %, mean = 94.9 %, max = 107.5 %, sd = 4.7 % (n = 89).

**Fig 3. HPLC chromatogram of phenolics in bilberry annual shoots**. The chromatograms shown here (**A** wavelength 280 nm, **B** wavelength 320 nm; retention time (x-axis) in minutes (min), response (y-axis) in mAU (AU = absorption units)) are from the subsample which was used to identify the peaks with mass spectrometry. Compounds identified (for footnotes, see S5 Supporting information): 1 protocatechuic acid derivative, 2 arbutin derivative^7^, 3 gallocatechin derivative, 4 procyanidin 1, 5 procyanidin 2, 6 epicatechin (formerly called: (-)-epicatechin), 7 procyanidin 3, 8 procyanidin 4, 9 procyanidin 5, 10 procyanidin 6, 11 chlorogenic acid, 12 para-hydroxycinnamic acid derivative 1, 13 cinnamic acid derivative, 14 para-hydroxycinnamic acid derivative 2, 15 hyperin^1^, 16 quercetin 3-glucuronide^5^, 17 quercetin 3-arabinoside^4^, 18 kaempferol 3-glucoside^2^, 19 quercitrin^6^, 20 isorhamnetin 3-glucoside, 21 para-hydroxycinnamic acid derivative 3, 22 monocoumaroyl-isoquercitrin^3,8^.

The phenolic concentration of all these 22 phenolics together (analysed together), from here called total phenolic concentration (mg/g, dry weight), varied between ramets. On average, almost 10 % of bilberry annual shoots (dry weight) consisted of these 22 phenolics (all data: min = 24.6, mean = 97.5, max = 164.9, sd = 25.9, se = 1.7 (n = 231)). The least severe simulated herbivory treatment, 'leaves cut 10 %', had often quite similar mean total phenolic concentrations as the control (S6 Supporting information). Compared to these, all treatments resulted in lower mean total phenolic concentrations, except the treatment 'annual shoots cut 10 %' in data-merged and the treatment 'leaves cut 50 %' in Vin-low which had higher total phenolic concentrations (S6 Supporting information). The differences between the latter two treatments and the control and least severe simulated herbivory treatment were not significant (Fig 4), but we did find a significant difference in total phenolic concentration between one or more treatments in data-merged (ANOVA: F_7,152_ = 7.04, *P* < 0.001) and Vin-low (Kruskal-Wallis: χ^2^ = 17.1, d.f. = 7, *P* = 0.02). Within the treatment group 'leaves cut' the total phenolic concentration did not differ significantly (Fig 4). Within the treatment group 'annual shoots cut' the total phenolic concentration did not differ significantly in Vin-low but in data-merged the most severe simulated herbivory treatment, 'annual shoots cut 100 %', resulted in a significantly lower total phenolic concentration than the least severe simulated herbivory treatment in this treatment group, 'annual shoots cut 10 %', while the "in between" treatment, 'annual shoots cut 50 %', did not significantly differ from either one of them (Fig 4). The 90 % ramet removal treatment, resulted in the lowest mean (S6 Supporting information) and median (Fig 4) total phenolic concentration. In data-merged, differences between this treatment and both the control and all treatments in the treatment group 'leaves cut' were significant (Fig 4). In Vin-low, the only significant difference between treatments was between the treatments 'ramet cut' and 'leaves cut 50 %' (Fig 4).

**Fig 4. Total phenolic concentration in bilberry annual shoots after simulated herbivory**. Boxplot with total concentration (mg/g, dry weight) for the merged dataset ('data-merged', n = 160, every treatment n = 20, dark grey boxes) to the left and data from location 'Vinjevegen', site 'low productivity' ('Vin-low', n = 40, every treatment n = 5, light grey boxes) to the right. The bottom and top of each box indicate the first and third quartiles. Bold horizontal lines within each box indicate median values. The plot whiskers extend to the most extreme data point which is no more than 1.5 times the interquartile range away from the box; extreme data points more than 1.5 times the interquartile range away from the box are indicated with black points. Treatments with the same letter above the box are not different from each other (*P* > 0.05).

As the total phenolic concentration is the sum of all individual phenolic concentrations, many individual phenolics showed a similar pattern: the most severe simulated herbivory treatment, 90 % ramet removal, resulted in the lowest mean phenolic concentration in 12 phenolics (55 %) in data-merged and 14 phenolics (64 %) in Vin-low (S6 Supporting information). Considering the two most severe simulated herbivory treatments together, this number increased to 18 phenolics (82 %) in data-merged (S6 Supporting information). Investigating significant differences between treatments, one or more simulated herbivory treatments resulted in significantly different individual phenolic concentrations in ten phenolics in data-merged and seven phenolics in Vin-low; phenolic acids showed little response to the treatments (Table 3). In the leaf removal treatments, there were no significant differences in individual phenolic concentration between treatments, but in one phenolic (epicatechin) the phenolic concentration in the control group was significantly higher than after leaf removal at a low level in data-merged (Fig 5). In the annual shoot removal treatments, in one phenolic a significant difference in phenolic concentration between treatments was present: in data-merged, the concentration of procyanidin 2 was significantly lower after leaf removal at an intermediate level than after leaf removal at a low level. Nevertheless, procyanidin 2 concentration in the annual shoot removal treatment at a high level was not significantly different from either one of the other level treatments (Fig 5). Additionally, the epicatechin, procyanidin 2 and quercetin 3-glucuronide concentrations in the control group were significantly higher than several annual shoot removal treatments in data-merged (Fig 5). If one or more significant differences between treatments were present in individual phenolics, often the most severe simulated herbivory treatment (90 % ramet removal) was one of the treatments involved, most often (only monocoumaroyl-isoquercitrin in data-merged deviates from this pattern) with a lower phenolic concentration than other treatments (Fig 5).

**Table 3. Differences in individual phenolic concentration in bilberry annual shoots between simulated herbivory treatments.** The number of differences between treatments (*P* < 0.05) is given for 22 phenolics separately, and for all 22 phenolics together, per dataset ('data-merged', n = 160; 'Vin-low', n = 40).

| **Flavonoids** | **Data-merged** | **Vin-low** |
| --- | --- | --- |
| Epicatechin | 6 | 1 |
| Gallocatechin derivative | ns | ns |
| Hyperin | 4 | 2 |
| Isorhamnetin 3-glucoside | ns | ns |
| Kaempferol 3-glucoside | 3 | ns |
| Monocoumaroyl-isoquercitrin^1^ | 2 | ns |
| Procyanidin 1 | ns | ns |
| Procyanidin 2 | 8 | 0 |
| Procyanidin 3 | ns | ns |
| Procyanidin 4 | 4 | 0 |
| Procyanidin 5 | 3 | 0 |
| Procyanidin 6 | ns | ns |
| Quercetin 3-arabinoside | 4 | 2 |
| Quercetin 3-glucuronide | 8 | 1 |
| Quercitrin | 0 | ns |
| ***Sum flavonoids*** | *42* | *6* |
| **Hydroquinones** |  |  |
| Arbutin derivative | 4 | 1 |
| ***Sum hydroquinones*** | *4* | *1* |
| **Phenolic acids** |  |  |
| Chlorogenic acid | ns | ns |
| Cinnamic acid derivative | ns | ns |
| Para-hydroxycinnamic acid derivative 1 | ns | 1 |
| Para-hydroxycinnamic acid derivative 2 | ns | ns |
| Para-hydroxycinnamic acid derivative 3 | ns | ns |
| Protocatechuic acid derivative | 0 | 1 |
| ***Sum phenolic acids*** | *0* | *2* |
| ***Sum all individual phenolics*** | *46* | *9* |
| **All 22 phenolics together** | 8 | 1 |

^1^Monocoumaroyl-isoquercitrin: identification uncertain.

If the ANOVA (data-merged) or Kruskal-Wallis (Vin-low) test result *P* < 0.05 but the Tukey's HSD or Dunn's test gave only *P*_adj_ values > 0.05 a '0' is shown in the table. ANOVA or Kruskal-Wallis test results *P* > 0.05 show 'ns' in the table (irrespective of the Tukey's HSD or Dunn's test result).

**Fig 5. Individual phenolic concentrations in bilberry annual shoots after simulated herbivory**. Boxplots with individual phenolic concentrations (mg/g, dry weight). Only phenolics where at least one treatment was significantly different from another treatment, in at least one dataset, are shown. Phenolics ordered as in Table 3 (flavonoids, hydroquinones, phenolic acids). Dataset 'data-merged': n = 160, every treatment n = 20, dark grey boxes to the left. Data from location 'Vinjevegen', site 'low productivity': 'Vin-low', n = 40, every treatment n = 5, light grey boxes to the right. The bottom and top of each box indicate the first and third quartiles. Bold horizontal lines within each box indicate median values. The plot whiskers extend to the most extreme data point which is no more than 1.5 times the interquartile range away from the box; extreme data points more than 1.5 times the interquartile range away from the box are indicated with black points. Treatments with the same letter above the box are not different from each other (*P* > 0.05). ns = no significant difference between any of the treatments.

Shoot carbon and nitrogen

Both the carbon and nitrogen concentration (mg/g, dry weight) and C/N ratio varied between ramets. On average, half of the bilberry annual shoots (dry weight) consisted of carbon and slightly over 1 % consisted of nitrogen. Values for the carbon concentration (mg/g), all data: min = 462, mean = 499, max = 548, sd = 11.8, se = 0.8 (n = 232). Values for the nitrogen concentration (mg/g), all data: min = 8.28, mean = 12.50, max = 21.00, sd = 2.01, se = 0.14 (n = 232). Values for the C/N ratio, all data: min = 24.7, mean = 41.0, max = 63.8, sd = 6.9, se = 0.5 (n = 232). There was little variation in carbon and nitrogen concentration between treatments in both data-merged and Vin-low (Table 4). Little variation in C/N ratio was present between treatments in data-merged and only slightly more variation was present in Vin-low (Table 4).

**Table 4. Carbon and nitrogen concentration and C/N ratio in bilberry annual shoots after simulated herbivory.** Mean concentration (mg/g ± standard error, dry weight) and mean C/N ratio (± standard error) for the merged dataset ('data-merged', n = 192) and for data from location 'Vinjevegen', site 'low productivity' ('Vin-low', n = 40).

|  | **Carbon concentration** | | **Nitrogen concentration** | | **C/N ratio** | |
| --- | --- | --- | --- | --- | --- | --- |
| **Treatment** | **Data-merged (n)** | **Vin-low (n)** | **Data-merged (n)** | **Vin-low (n)** | **Data-merged (n)** | **Vin-low (n)** |
| Control | 498 ± 2.2 (28) | 500 ± 5.8 (5) | 12.55 ± 0.38 (28) | 10.73 ± 0.86 (5) | 40.7 ± 1.2 (28) | 48.3 ± 3.4 (5) |
| Leaves cut 10 % | 497 ± 2.2 (28) | 499 ± 5.8 (5) | 12.65 ± 0.38 (28) | 11.03 ± 0.86 (5) | 39.9 ± 1.2 (28) | 46.5 ± 3.4 (5) |
| Leaves cut 50 % | 498 ± 2.2 (27) | 495 ± 5.8 (5) | 12.83 ± 0.39 (27) | 11.62 ± 0.86 (5) | 39.6 ± 1.2 (27) | 43.0 ± 3.4 (5) |
| Leaves cut 100 % | 499 ± 2.2 (27) | 496 ± 5.8 (5) | 12.50 ± 0.39 (27) | 10.99 ± 0.86 (5) | 40.7 ± 1.2 (27) | 46.8 ± 3.4 (5) |
| Annual shoots cut 10 % | 498 ± 2.6 (20) | 508 ± 5.8 (5) | 12.23 ± 0.45 (20) | 11.90 ± 0.86 (5) | 41.8 ± 1.4 (20) | 43.0 ± 3.4 (5) |
| Annual shoots cut 50 % | 498 ± 2.6 (20) | 499 ± 5.8 (5) | 13.12 ± 0.45 (20) | 12.23 ± 0.86 (5) | 38.9 ± 1.4 (20) | 42.0 ± 3.4 (5) |
| Annual shoots cut 100 % | 506 ± 2.5 (21) | 497 ± 5.8 (5) | 13.01 ± 0.44 (21) | 11.73 ± 0.86 (5) | 40.0 ± 1.4 (21) | 43.7 ± 3.4 (5) |
| Ramet cut | 500 ± 2.5 (21) | 502 ± 5.8 (5) | 13.32 ± 0.44 (21) | 9.69 ± 0.86 (5) | 38.8 ± 1.4 (21) | 51.9 ± 3.4 (5) |
| All treatments together | 499 ± 0.8 (192) | 500 ± 2.0 (40) | 12.76 ± 0.15 (192) | 11.24 ± 0.30 (40) | 40.1 ± 0.5 (192) | 45.7 ± 1.2 (40) |

We found no significant difference in C/N ratio between treatments in data-merged (ANOVA: F_7,152_ = 0.55, *P* = 0.79) or Vin-low (Kruskal-Wallis: χ^2^ = 7.1, d.f. = 7, *P* = 0.42). Analyses of only carbon concentration or nitrogen concentration yielded similar results: no differences between treatments (carbon, data-merged: ANOVA: F_7,152_ = 1.48, *P* = 0.18; carbon, Vin-low: Kruskal-Wallis: χ^2^ = 5.7, d.f. = 7, *P* = 0.57; nitrogen, data-merged: ANOVA: F_7,152_ = 0.66, *P* = 0.70; nitrogen, Vin-low: Kruskal-Wallis: χ^2^ = 7.3, d.f. = 7, *P* = 0.39).

**Discussion**

We did not find (+)-catechin in our bilberry annual shoots, as has been found in other studies of bilberry [49, 50]. In the HPLC chromatogram (Fig 3), (+)-catechin,if present, comes shortly after chlorogenic acid. This means that when a large quantity of chlorogenic acid is present, as with our subsamples (Fig 3, S6 Supporting information), the peak for chlorogenic acid overlaps with the peak for (+)-catechin and it is not possible to separate the latter from the former, especially when only little (+)-catechin is present. Therefore, unidentified amounts of (+)-catechin may have been present in our subsamples in cases where (+)-catechin was present in much lower amounts than epicatechin (S6 Supporting information).

Our first prediction was: In bilberry annual shoots, the concentration of phenolics synthesized without direct competition with protein synthesis is, at low to intermediate (simulated) herbivory levels, positively correlated with simulated herbivory. Each of the 22 phenolic compounds found in our samples have phenylalanine as a precursor and are therefore synthesized in direct competition with protein synthesis. Gallic acid derivatives and e.g hydrolyzable tannins such as pentagalloylglucose (which can be extracted with methanol and traces of these compounds have been found in bilberry [122-124]) were detected in our subsamples, leaving no possibility to evaluate our first prediction.

Our second prediction was: In bilberry annual shoots, the concentration of phenolics synthesized in direct competition with protein synthesis is, at low to intermediate (simulated) herbivory levels, not correlated with simulated herbivory. As all identified phenolics are formed in direct competition with protein synthesis, we can use both individual and total phenolic concentrations to evaluate prediction II. Excluding simulated herbivory at high simulated herbivory levels, we can discriminate three herbivory levels in both treatment groups 'leaves cut' and 'annual shoots cut'. These are, ordered at increasing herbivory level: control, leaves cut 10 %, leaves cut 50 % and control, annual shoots cut 10 %, annual shoots cut 50 % in the treatment group 'leaves cut' and 'annual shoots cut', respectively. In the 'leaves cut' treatment, the total phenolic concentration did not differ significantly among herbivory levels, in eitherdataset (Fig 4), supporting prediction II. Similarly, for Vin-low, in the treatment group 'annual shoots cut' the total phenolic concentration of the three mentioned herbivory levels did not differ significantly, also supporting prediction II (Fig 4). When data were merged, however, the 'annual shoots cut 50 %' treatment showed a significantly lower total phenolic concentration than the control (Fig 4), challenging prediction II. That this same treatment represents the greatest hervivory damage supports prediction III.

Individual phenolic results from the merged dataset supported prediction II largely, apart from three flavonoids whose concentrations differed significantly between some of the three mentioned herbivory levels: epicatechin, procyanidin 2 and quercetin 3-glucuronide (Fig 5). Here, the treatment 'annual shoots cut 50 %' resulted in a significantly lower phenolic concentration than the control (Fig 5), supporting the suggestion that this treatment could be considered, in bilberry, as simulated herbivory at a high level instead of an intermediate level and thereby supporting prediction III (see further). In the dataset Vin-low, no significant differences between the three mentioned herbivory levels were present, completely supporting prediction II (Fig 5). Studies on leaf instead of shoot chemistry have found a positive correlation between non-severe defoliation and concentration of phenolics or condensed tannins in leaves of deciduous woody plant species [37, 125, 126]. In these studies, phenolics were identified on group level: condensed tannins and total polyphenols. Therefore, the (partly) presence of phenolics synthesized without competition with protein synthesis cannot be ruled out, which would (partly) explain this positive correlation. The species used in these studies were mountain birch (*Betula pubescens* ssp. *tortuosa* (Ledeb.) Nyman, today *B. pubescens* var. *pumila* (L.) Govaerts; worldfloraonline.org) and mopane (*Colophospermum mopane* (J.Kirk ex Benth.) J.Léonard). Indeed, mountain birch leaves contain hydrolyzable phenolics [127], which can be synthesized without competition with protein synthesis. Furthermore, species specific traits (like inherently slow-growing versus fast-growing species), which may be different in mountain birch, mopane and bilberry, may affect responses on leaf herbivory. Additionally, mopane saplings were used: in a similar experiment, the observed increase in foliar condensed tannin concentration after defoliation was probably the result of plant aging, not a response to defoliation [128]. Although these studies on foliar phenolics did not involve shoot phenolic concentrations, Persson and colleagues [55] found that bilberry leaves and bilberry leafless shoots were comparable in their response to simulated moose herbivory, at least for flavonoids and condensed tannins. Our results contrast with Persson and colleagues, who found an increase in flavonoid and condensed tannin concentration in bilberry shoots with increasing simulated moose herbivory. Possibly their results were influenced by a side-effect of the treatment: a more open canopy resulted in more solar radiation which could have induced production of secondary compounds, as has been found and discussed in other studies [50, 55, 64, 129-133].

As bilberry is a clonal plant, connected ramets may translocate compounds from nondefoliated to defoliated ramets, as has been documented in perennial graminoid species and herbs [134, 135, 136, and references herein]. Translocation of carbohydrates or even phenolics between connected ramets may be, next to the described competition with protein synthesis, another reason for the absence of a correlation between simulated leaf herbivory and subsequent phenolic concentrations in bilberry annual shoots. Also, high fine root mortality (leading to decreased nutrient uptake and consequent increased carbon-based secondary compounds in the foliage [91]) resulting from severe defoliation may not, or in a lesser extent, occur in clonal bilberry, possibly translocating carbohydrates between connected ramets to maintain its root activity, which may also contribute to the mentioned absence of a significant correlation.

Furthermore, our leaf simulated herbivory (mechanical wounding by hand) is not equivalent to leaf herbivory by insects, birds or small mammals [27, 137-145, reviewed by 146]. Although most of these studies indicate that simulated leaf and shoot herbivory performed by mechanical wounding induces a less pronounced plant response, such simulated herbivory generally does induce a plant response [see the aformentioned references and 97, 147, 148], particularly in nutrient-poor sites [149] (similar to our study system), as the general response of plants to wounding and herbivore damage is essentially the same [150]. Nevertheless, this indicates that bilberry responses to simulated leaf herbivory may differ from responses to natural herbivory, which can further contribute to our observed absence of a correlation between simulated leaf herbivory and subsequent phenolic concentrations in bilberry annual shoots.

Additionally, this observed absence can be due to other reasons. The time between our simulated leaf herbivory treatments and bilberry ramet harvesting was 48-68 days. Possibly, bilberry only responds with a short-term response that was no longer detectable after 48 days (in another woody species, the condensed tannin concentration returned to pre-herbivory values less than 66 hours after herbivory [151, see also 152]). An alternative possible reason is a very delayed response: responses remain undetectable until at least 68 days after the treatment. This last option seems very unlikely in terms of plant fitness, but cannot be ruled out with the data available. Also, a response can have been counteracted by transport of existing phenolics from shoots to leaves, as some plant species store phenolics in shoots which are transported to leaves following herbivory [147] – although such reallocation of phenolics may not be very important [56]. Another possible reason for our observed results is that a change in phenolics occurs in other plant parts, e.g., leaves, and is not detectable in annual shoots. As we do not have data about phenolics in plant parts other than annual shoots, we cannot rule out this possibility.

Finally, in spite of the aformentioned possible reasons to explain our observed absence of a correlation between simulated leaf herbivory and subsequent phenolic concentrations in bilberry annual shoots, it may be that bilberry does not respond to simulated herbivory at low to intermediate (simulated) herbivory levels at all. We conclude that, accordingly to our prediction II and the Protein Competition Model, bilberry annual shoot phenolics that are synthesized in direct competition with protein synthesis (e.g., (hydroxy)cinnamic acids and condensed tannins) do not follow the Carbon:Nutrient Balance hypothesis after simulated herbivory at low to intermediate levels.

Our third prediction was: In bilberry annual shoots, the concentration of phenolics is, at high simulated herbivory levels, lower than without simulated herbivory. In our study, we considered three treatments as simulated herbivory at high levels: complete leaf removal (within the group leaf removal treatments), complete annual shoot removal (within the group annual shoot removal treatments) and the treatment 'ramet cut' (considered as herbivory at the highest level in our study). The first one, complete leaf removal, did not significantly differ from the control or any other leaf removal treatment in tannin concentration (Fig 2), total phenolic concentration (Fig 4) nor in any individual phenolic concentration (Fig 5). Apparently, bilberry is still able to allocate carbon to phenolics after complete defoliation. The aforementioned possible translocation of carbohydrates, or even phenolics, between connected ramets of this clonal species contributes to this finding. Interestingly, although carbon allocation to phenolics apparently did not decrease, no increase of phenolic concentrations was present after complete defoliation, which is predicted by the Optimal Defense hypotheses and has been reported by other studies, although these investigated only foliar phenolic concentrations, no shoot phenolic data was available [87 (a tendency, not significant), 89, 153]. Different from our study, in these studies evergreen species were used, which respond differently than deciduous species ([19, 154]). As said, we did not find a decrease in phenolic concentrations after complete defoliation, which has been reported by Bryant and colleagues for foliage of slow-growing woody species [154]. In their study, fast-growing deciduous species responded differently (leaf total phenol and condensed tannin concentrations increased after manual defoliation) than slow-growing evergreen species (leaf total phenol and condensed tannin concentrations decreased after manual defoliation), in accordance with the Carbon:Nutrient Balance hypothesis [19, 154] (although many phenolics may not follow this pattern according to the Protein Competition Model). Our research object is, unlike the woody species in the study by Bryant and colleagues, a slow-growing deciduous plant in which severe defoliation impacts nutrient reserves more than carbon reserves (see Introduction), resulting in our predictions II and III. As we did not find significantly different phenolic concentrations after severe defoliation compared to little defoliation and the control, this can be interpreted as extra support for prediction II, when considering complete defoliation as simulated herbivory at an intermediate level instead of a high level. Additionally, the difference between simulated leaf herbivory and natural herbivory (see before) may have contributed to the absence of significantly different phenolic concentrations after severe defoliation compared to less defoliation and the control.

When simulated herbivory concerned the annual shoot removal treatments and the treatment 'ramet cut', we found support for prediction III in the largest dataset (data-merged): both the most severe annual shoot removal treatment and the treatment 'ramet cut', not significantly different from each other, resulted in significantly lower tannin concentrations and total phenolic concentrations than both the 10 % annual shoot removal treatment and the control (Fig 2, 4). The difference between the merged dataset and Vin-low may be due to the lower number of observations in Vin-low (only five observations per treatment). Although not significant, also in Vin-low, a tendency towards a lower tannin and total phenolic concentration after simulated herbivory at the highest level compared to natural herbivory at low to intermediate levels (ambient herbivory) was visible (Fig. 2, 4). This tendency was also visible in many individual phenolics (Fig. 5). These findings are in line with results from studies on effects of severe herbivory on phenolic concentrations in new plant tissue (shoots and leaves) in other woody plant species [80, although shoot browsing level in this study was 30% - but this may be considered severe browsing, see the suggestion before), 155, 156], but contrast with results from Persson and colleagues, who found, , an increase in flavonoid and condensed tannin concentration in bilberry shoots with increasing simulated moose herbivory, possibly caused by increased solar radiation (see under prediction II). Interestingly, Persson and colleagues reported that cinnamic acids in bilberry shoots behaved differently from flavonoids, as all cinnamic acid concentrations decreased with increasing moose density and light. They speculated that this was a result of an increase in flavonoid concentration, as some cinnamic acids are precursors for flavonoids [42, 55, 157]. In our study, we identified one cinnamic acid derivative and three para-hydroxycinnamic acid derivatives: opposed to more than half of all flavonoids and the only identified hydroquinone, for these cinnamic acids we found no significant differences between treatments in the merged dataset (Fig 5). This may be due to an increase in flavonoid concentration, as speculated by Persson and colleagues.

That these considerations show our results to clearly support prediction III, we conclude that neitherthe Carbon:Nutrient Balance hypothesis, nor the Optimal Defense hypothesis, cant be used to predict changes in phenolic compound concentrations in bilberry annual shoots after severe herbivory: in this situation, carbon is primarily used for purposes (e.g. growth) other than defense.

Our fourth prediction was: In bilberry annual shoots, the concentration of nitrogen is, at low to intermediate (simulated) herbivory levels, positively correlated with simulated herbivory, i.e., the C/N ratio is negatively correlated with simulated herbivory. Our fifth prediction was: In bilberry annual shoots, the concentration of nitrogen is, at high simulated herbivory levels, lower, i.e., the C/N ratio is higher, than without simulated herbivory, especially in bilberry growing on relatively nutrient-poor soil. The nitrogen and carbon concentrations and C/N ratio in our study are comparable with results from other studies [6, 50, 131, 158, 159] but differ from bilberry nitrogen concentrations found by Selås and colleagues [160]. Our results do not support prediction IV and V., in clonal bilberry connected ramets may translocate compounds. At low to intermediate (simulated) herbivory levels (prediction IV), carbohydrates may be translocated from source ramets to ramets under simulated herbivory pressure to compensate for a lack of carbon. And vice versa: if the intact root system of a ramet under herbivory pressure takes up nitrogen beyond the ramet's need, the surplus of nitrogen may be translocated to other connected ramets. At high herbivory levels (prediction V), carbohydrates may be translocated from source ramets to ramets under simulated herbivory pressure, and to their root system. This prevents an increase in fine root mortality and consequent a decrease in nutrient concentration. Thus, clonality can explain the lack of support for our predictions IV and V. Another possible reason is, as with phenolics (see before), that a change in C/N ratio occurs not in bilberry annual shoots but in other plant parts, e.g., leaves, as shown in other woody species [80, 82, 85, 105, 125] (although Laine and Henttonen [158] did not find a correlation between microtine density and nitrogen concentration in bilberry leaves). As we do not have data about carbon and nitrogen concentrations in plant parts other than annual shoots, we cannot rule out this possibility. Finally, Flower-Ellis reported much variation in nitrogen concentration between long, vegetative shoots and short, predominantly flowering shoots, as well as in ramets from different ages and position in the stand (causing variation in light and water conditions) [6]. Such variation may obscure possible effects from simulated herbivory.

**Conclusions**

We conclude that, according to our prediction II and the Protein Competition Model, bilberry annual shoot phenolics that are synthesized in direct competition with protein synthesis do not follow the predictions of the Carbon:Nutrient Balance hypothesis after simulated herbivory at low to intermediate levels. Furthermore, complete defoliation of bilberry ramets can be interpreted, at ramet level, as simulated herbivory at an intermediate rather than a high level. To the contrary, the treatment 'annual shoots cut 50 %' can be interpreted, at ramet level, as simulated herbivory at a high level, not an intermediate level, but only in relation tothetotal concentration of phenolics that were synthesized in direct competition with protein. Simulated herbivory at a high level, but not at lower levels, decreases tannin concentrations and total phenolic concentrations (of phenolics that were synthesized in direct competition with protein) compared to natural herbivory at low to intermediate levels (ambient herbivory), supporting our prediction III. Therefore, we conclude that neither the Optimal Defense nor the Carbon:Nutrient Balance hypothesis canbe used to predict changes in phenolic compound concentrations in bilberry annual shoots after severe simulated herbivory. In this situation, carbon is primarily used for purposes other than defense (e.g. growth).

**Acknowledgments**

We thank Anne Mehlhoop, Bernardo Toledo González and Marieke Gonlag-Schrijvers for lab work and analytical insights, Sinikka Sorsa, Katri Nissinen, Virpi Virjamo and Md. Nazmul Hasan for help in the lab, Anne Mehlhoop, Andreja Kovše and Umer Qureshi for soil sampling, Maria Greger for initial advice on soil sampling and phenolics, Gé van Steijn for comments on the analyses, and Jo Inge Breisjøberget and Kjell Anders Vikan for information about and digital maps based on the H40 system. We thank the Norwegian state-owned land and forest enterprise Statskog SF for permission to do the experiment on their property. MSG thanks the Stack Exchange Q&A web communities Stack Overflow and Cross Validated for invaluable statistical and analytical insights, and Ole Arne Hagen / Linnea AS for facilitating a productive working environment during a pandemic.

**References**

1.	Fremstad E. Vegetasjonstyper i Norge. 2 ed. Trondheim: Norsk institutt for naturforskning; 1997. 279 p.

2.	Mossberg B, Stenberg L. Nordens flora. Stockholm: Bonnier Fakta; 2018. 976 p.

3.	Boonstra R, Andreassen HP, Boutin S, Hušek J, Ims RA, Krebs CJ, et al. Why do the boreal forest ecosystems of northwestern Europe differ from those of western North America? Bioscience. 2016;66(9):722-34. doi: 10.1093/biosci/biw080.

4.	Nilsson MC, Wardle DA. Understory vegetation as a forest ecosystem driver: evidence from the northern Swedish boreal forest. Front Ecol Environ. 2005;3(8):421-8. doi: 10.2307/3868658. PubMed PMID: WOS:000232295800015.

5.	Ritchie JC. *Vaccinium myrtillus* L. J Ecol. 1956;44(1):291-9. doi: 10.2307/2257181.

6.	Flower-Ellis JGK. Age structure and dynamics in stands of bilberry (*Vaccinium myrtillus* L.) [PhD dissertation]. Stockholm: Royal college of forestry; 1971.

7.	Nestby R, Percival D, Martinussen I, Opstad N, Rohloff J. The European blueberry (*Vaccinium myrtillus* L.) and the potential for cultivation. A review. The European Journal of Plant Science and Biotechnology. 2011;5(1):5-16.

8.	Tolvanen A, Laine K. Effects of reproduction and artificial herbivory on vegetative growth and resource levels in deciduous and evergreen dwarf shrubs. Can J Bot. 1997;75(4):656-66. PubMed PMID: ISI:A1997XD78600016.

9.	Cederlund G, Ljunqvist H, Markgren G, Stålfelt F. Foods of moose and roe-deer at Grimsö in central Sweden. Results of rumen content analyses. Swedish Wildlife Research - Viltrevy. 1980;11(4):169-247.

10.	Spidsø TK. Food selection by Willow Grouse *Lagopus lagopus* chicks in northern Norway. Ornis Scand. 1980;11:99-105.

11.	Viro P, Sulkava S. Food of the bank vole in northern Finnish spruce forests. Acta Theriol (Warsz). 1985;30(9-20):259-66. PubMed PMID: ISI:A1985A678000007.

12.	Atlegrim O. Exclusion of birds from bilberry stands - impact on insect larval density and damage to the bilberry. Oecologia. 1989;79(1):136-9. doi: 10.1007/Bf00378251. PubMed PMID: ISI:A1989U184700021.

13.	Hjältén J, Danell K, Ericson L. Hare and vole browsing preferences during winter. Acta Theriol (Warsz). 2004;49(1):53-62. doi: 10.1007/Bf03192508. PubMed PMID: ISI:000220756600005.

14.	Wegge P, Kastdalen L. Habitat and diet of young grouse broods: resource partitioning between Capercaillie (*Tetrao urogallus*) and Black Grouse (*Tetrao tetrix*) in boreal forests. J Ornithol. 2008;149(2):237-44. doi: 10.1007/s10336-007-0265-7. PubMed PMID: ISI:000254237400012.

15.	Soininen EM, Ravolainen VT, Brathen KA, Yoccoz NG, Gielly L, Ims RA. Arctic small rodents have diverse diets and flexible food selection. PLoS ONE. 2013;8(6). doi: 10.1371/journal.pone.0068128. PubMed PMID: WOS:000321150000125.

16.	Frøstrup JC. Hare og harejakt. Oslo: Teknologisk forl.; 1996. 123 p.

17.	Karban R, Baldwin IT. Induced responses to herbivory. Chicago, IL, USA: University of Chicago Press; 1997. X+320 p.

18.	Skarpe C, Hester AJ. Plant traits, browsing and grazing herbivores, and vegetation dynamics. In: Gordon IJ, Prins HHT, editors. The ecology of browsing and grazing. Berlin: Springer; 2008. p. 217-47.

19.	Herms DA, Mattson WJ. The dilemma of plants - to grow or defend. Q Rev Biol. 1992;67(3):283-335. PubMed PMID: ISI:A1992JR52500002.

20.	McKey D. Adaptive patterns in alkaloid physiology. Am Nat. 1974;108(961):305-20.

21.	Rhoades DF. Evolution of plant chemical defense against herbivores. Herbivores: their interaction with secondary plant metabolites. New York: Academic Press; 1979. p. 3-54.

22.	Fagerström T, Larsson S, Tenow O. On optimal defence in plants. Funct Ecol. 1987;1(2):73-81.

23.	Karban R, Agrawal AA, Thaler JS, Adler LS. Induced plant responses and information content about risk of herbivory. Trends Ecol Evol. 1999;14(11):443-7. doi: 10.1016/S0169-5347(99)01678-X. PubMed PMID: WOS:000083141800010.

24.	Stamp N. Out of the quagmire of plant defense hypotheses. Q Rev Biol. 2003;78(1):23-55. PubMed PMID: ISI:000181483100002.

25.	Kessler A. The information landscape of plant constitutive and induced secondary metabolite production. Curr Opin Insect Sci. 2015;8:47-53. doi: 10.1016/j.cois.2015.02.002. PubMed PMID: WOS:000369017000010.

26.	Bryant JP, Julkunen-Tiitto R. Ontogenic development of chemical defense by seedling resin birch: Energy cost of defense production. J Chem Ecol. 1995;21(7):883-96. doi: 10.1007/BF02033796.

27.	Mithöfer A, Boland W, Maffei ME. Chemical ecology of plant–insect interactions. In: Parker J, editor. Annual Plant Reviews volume 34: Molecular aspects of plant disease resistance 2009. p. 261-91.

28.	Massad TJ, Trumbore SE, Ganbat G, Reichelt M, Unsicker S, Boeckler A, et al. An optimal defense strategy for phenolic glycoside production in *Populus trichocarpa*-isotope labeling demonstrates secondary metabolite production in growing leaves. New Phytol. 2014;203(2):607-19. doi: 10.1111/nph.12811. PubMed PMID: 24739022.

29.	Waring RH, Pitman GB. Modifying lodgepole pine stands to change susceptibility to mountain pine beetle attack. Ecology. 1985;66(3):889-97. doi: 10.2307/1940551.

30.	Tuomi J, Niemelä P, Stuart Chapin F, Bryant JP, Sirén S. Defensive responses of trees in relation to their carbon/nutrient balance. Mechanisms of woody plant defenses against insects. New York, NY: Springer-Verlag; 1988. p. 57-72.

31.	Gayler S, Grams TEE, Heller W, Treutter D, Priesack E. A dynamical model of environmental effects on allocation to carbon-based secondary compounds in juvenile trees. Ann Bot. 2008;101(8):1089-98. doi: 10.1093/aob/mcm169.

32.	Skarpe C, Van der Wal R. Effects of simulated browsing and length of growing season on leaf characteristics and flowering in a deciduous Arctic shrub, *Salix polaris*. Arct Antarct Alp Res. 2002;34(3):282-6. PubMed PMID: ISI:000178100600006.

33.	Li R, Zhang J, Li J, Zhou G, Wang Q, Bian W, et al. Prioritizing plant defence over growth through WRKY regulation facilitates infestation by non-target herbivores. eLife. 2015;4:e04805. doi: 10.7554/eLife.04805.

34.	Coley PD, Bryant JP, Chapin FS. Resource availability and plant antiherbivore defense. Science. 1985;230(4728):895-9. PubMed PMID: ISI:A1985AUA8000007.

35.	Barz W, Hoesel W. Metabolism and degradation of phenolic compounds in plants. In: Swain T, Harborne JB, Van Sumere CF, editors. Biochemistry of plant phenolics. New York: Plenum Press; 1979. p. 339-69.

36.	Gershenzon J. Changes in the levels of plant secondary metabolites under water and nutrient stress. In: Timmermann BN, Steelink C, Loewus FA, editors. Phytochemical Adaptations to Stress. Boston, MA: Springer US; 1984. p. 273-320.

37.	Kohi EM, De Boer WF, Slot M, Van Wieren SE, Ferwerda JG, Grant RC, et al. Effects of simulated browsing on growth and leaf chemical properties in *Colophospermum mopane* saplings. African Journal of Ecology. 2010;48(1):190-6. doi: 10.1111/j.1365-2028.2009.01099.x.

38.	Tuomi J, Fagerstrom T, Niemela P. Carbon allocation, phenotypic plasticity, and induced defenses. In: Tallamy DW, Raupp MJ, editors. Phytochemical induction by herbivores. New York/ Chichester/ Brisbane/ Totonto/ Singapore: Wiley; 1991. p. 85-104.

39.	Lähdesmäki P, Pakonen T, Saari E, Laine K, Havas P. Environmental factors affecting basic nitrogen metabolism and seasonal levels of various nitrogen fractions in tissues of bilberry, *Vaccinium myrtillus*. Holarctic Ecology. 1990;13(1):19-30.

40.	Bryant JP, Chapin FS, Klein DR. Carbon nutrient balance of boreal plants in relation to vertebrate herbivory. Oikos. 1983;40(3):357-68. PubMed PMID: ISI:A1983QS70400005.

41.	Engbersen JFJ, De Groot Æ. Bio-organische chemie. Fifth ed. Wageningen: Pudoc; 1992. 596 p.

42.	Vermerris W, Nicholson RL. Phenolic compound biochemistry: Springer; 2008. XII+276 p.

43.	Bennett RN, Wallsgrove RM. Secondary metabolites in plant defence mechanisms. New Phytol. 1994;127(4):617-33. doi: doi:10.1111/j.1469-8137.1994.tb02968.x.

44.	Dearing MD. Effects of *Acomastylis rossii* tannins on a mammalian herbivore, the North American pika, *Ochotona princeps*. Oecologia. 1996;109(1):122-31. doi: 10.1007/s004420050066.

45.	Felton GW, Donato KK, Broadway RM, Duffey SS. Impact of oxidized plant phenolics on the nutritional quality of dietar protein to a noctuid herbivore, *Spodoptera exigua*. J Insect Physiol. 1992;38(4):277-85. doi: 10.1016/0022-1910(92)90128-Z.

46.	Tahvanainen J, Niemela P, Henttonen H. Chemical aspects of herbivory in boreal forest - feeding by small rodents, hares, and cervids. In: Palo RT, Robbins CT, editors. Plant defenses against mammalian herbivory. Boca Raton: CRC Press; 1991. p. 115-31.

47.	Tahvanainen J, Julkunen-Tiitto R, Kettunen J. Phenolic glycosides govern the food selection pattern of willow feeding leaf beetles. Oecologia. 1985;67(1):52-6. doi: 10.1007/Bf00378451. PubMed PMID: WOS:A1985ANM6000009.

48.	Seigler DS. Plant secondary metabolism. Boston: Kluwer; 1998. IX+759 p.

49.	Bujor OC, Le Bourvellec C, Volf I, Popa VI, Dufour C. Seasonal variations of the phenolic constituents in bilberry (*Vaccinium myrtillus* L.) leaves, stems and fruits, and their antioxidant activity. Food Chem. 2016;213:58-68. doi: 10.1016/j.foodchem.2016.06.042. PubMed PMID: WOS:000380289900008.

50.	Nybakken L, Selås V, Ohlson M. Increased growth and phenolic compounds in bilberry (*Vaccinium myrtillus* L.) following forest clear-cutting. Scand J For Res. 2013;28(4):319-30. doi: 10.1080/02827581.2012.749941.

51.	Latti AK, Riihinen KR, Jaakola L. Phenolic compounds in berries and flowers of a natural hybrid between bilberry and lingonberry (*Vaccinium* x *intermedium* Ruthe). Phytochemistry. 2011;72(8):810-5. doi: 10.1016/j.phytochem.2011.02.015. PubMed PMID: ISI:000290505500018.

52.	Riihinen K, Jaakola L, Karenlampi S, Hohtola A. Organ-specific distribution of phenolic compounds in bilberry (*Vaccinium myrtillus*) and 'northblue' blueberry (*Vaccinium* *corymbosum* x *V. angustifolium*). Food Chem. 2008;110(1):156-60. doi: 10.1016/j.foodchem.2008.01.057. PubMed PMID: ISI:000255729700020.

53.	Martz F, Jaakola L, Julkunen-Tiitto R, Stark S. Phenolic composition and antioxidant capacity of bilberry (*Vaccinium myrtillus*) leaves in northern Europe following foliar development and along environmental gradients. J Chem Ecol. 2010;36(9):1017-28. doi: 10.1007/s10886-010-9836-9. PubMed PMID: WOS:000281971400012.

54.	Witzell J, Gref R, Nasholm T. Plant-part specific and temporal variation in phenolic compounds of boreal bilberry (*Vaccinium myrtillus*) plants. Biochem Syst Ecol. 2003;31(2):115-27. doi: 10.1016/S0305-1978(02)00141-2. PubMed PMID: WOS:000180726900001.

55.	Persson IL, Julkunen-Tiitto R, Bergström R, Wallgren M, Suominen O, Danell K. Simulated moose (*Alces alces* L.) browsing increases accumulation of secondary metabolites in bilberry (*Vaccinium myrtillus* L.) along gradients of habitat productivity and solar radiation. J Chem Ecol. 2012;38(10):1225-34. doi: 10.1007/s10886-012-0209-4. PubMed PMID: WOS:000311394500003.

56.	Jones CG, Hartley SE. A protein competition model of phenolic allocation. Oikos. 1999;86(1):27-44. doi: 10.2307/3546567.

57.	Haukioja E, Ossipov V, Koricheva J, Honkanen T, Larsson S, Lempa K. Biosynthetic origin of carbon-based secondary compounds: cause of variable responses of woody plants to fertilization? Chemoecology. 1998;8(3):133-9. doi: 10.1007/s000490050018. PubMed PMID: ISI:000208087000005.

58.	Koricheva J, Barton K. Temporal changes in plant secondary metabolite production: patterns, causes and consequences. In: Iason GR, Dicke M, Hartley SE, editors. The ecology of plant secondary metabolites. From genes to global processes. Ecological Reviews. Cambridge: Cambridge University Press; 2012. p. 34-55.

59.	Elser JJ, Bracken MES, Cleland EE, Gruner DS, Harpole WS, Hillebrand H, et al. Global analysis of nitrogen and phosphorus limitation of primary producers in freshwater, marine and terrestrial ecosystems. Ecol Lett. 2007;10(12):1135-42. doi: 10.1111/j.1461-0248.2007.01113.x.

60.	Menge DNL, Hedin LO, Pacala SW. Nitrogen and phosphorus limitation over long-term ecosystem development in terrestrial ecosystems. PLoS ONE. 2012;7(8):e42045-e. doi: 10.1371/journal.pone.0042045. PubMed PMID: 22870281.

61.	Vitousek PM, Howarth RW. Nitrogen limitation on land and in the sea - how can it occur. Biogeochemistry. 1991;13(2):87-115. PubMed PMID: WOS:A1991GH74100001.

62.	Tamm CO. Nitrogen in terrestrial ecosystems: questions of productivity, vegetational changes, and ecosystem stability. Billings WD, Golley F, Lange OL, Olson JS, Remmert H, editors. Berlin: Springer-Verlag; 1991. XII+116 p.

63.	Ossipov V, Salminen J-P, Ossipova S, Haukioja E, Pihlaja K. Gallic acid and hydrolysable tannins are formed in birch leaves from an intermediate compound of the shikimate pathway. Biochem Syst Ecol. 2003;31(1):3-16. doi: 10.1016/S0305-1978(02)00081-9.

64.	Gutzeit HO, Ludwig-Müller J. Plant natural products: synthesis, biological functions and practical applications. Weinheim: John Wiley & Sons; 2014. 422 p.

65.	Agati G, Azzarello E, Pollastri S, Tattini M. Flavonoids as antioxidants in plants: Location and functional significance. Plant Sci. 2012;196:67-76. doi: 10.1016/j.plantsci.2012.07.014.

66.	Asplund J, van Zuijlen K, Roos RE, Birkemoe T, Klanderud K, Lang SI, et al. Contrasting responses of plant and lichen carbon-based secondary compounds across an elevational gradient. Funct Ecol. 2021;35(2):330-41. doi: 10.1111/1365-2435.13712.

67.	Gauslaa Y. Lichen palatability depends on investments in herbivore defence. Oecologia. 2005;143(1):94-105. doi: 10.1007/s00442-004-1768-z.

68.	Lokvam J, Kursar TA. Divergence in structure and activity of phenolic defenses in young leaves of two co-occurring *Inga* species. J Chem Ecol. 2005;31(11):2563-80. doi: 10.1007/s10886-005-7614-x.

69.	Bernays EA. Plant tannins and insect herbivores: an appraisal. Ecol Entomol. 1981;6(4):353-60. doi: 10.1111/j.1365-2311.1981.tb00625.x.

70.	Solhaug KA, Gauslaa Y. Secondary lichen compounds as protection against excess solar radiation and herbivores. In: Lüttge U, Beyschlag W, Büdel B, Francis D, editors. Progress in Botany 73. Berlin, Heidelberg: Springer; 2012. p. 283-304.

71.	Molnár K, Farkas E. Current results on biological activities of lichen secondary metabolites: a review. Zeitschrift für Naturforschung C. 2010;65(3-4):157-73. doi: 10.1515/znc-2010-3-401.

72.	Virjamo V, Julkunen-Tiitto R, Henttonen H, Hiltunen E, Karjalainen R, Korhonen J, et al. Differences in vole preference, secondary chemistry and nutrient levels between naturally regenerated and planted Norway spruce seedlings. J Chem Ecol. 2013;39(10):1322-34. doi: 10.1007/s10886-013-0352-6. PubMed PMID: WOS:000326456100009.

73.	Mattson WJ. Herbivory in relation to plant nitrogen content. Annu Rev Ecol Syst. 1980;11(1):119-61.

74.	Lundberg P, Astrom M. Low nutritive quality as a defense against optimally foraging herbivores. Am Nat. 1990;135(4):547-62. doi: 10.1086/285061.

75.	Schädler M, Jung G, Auge H, Brandl R. Palatability, decomposition and insect herbivory: patterns in a successional old-field plant community. Oikos. 2003;103(1):121-32. doi: 10.1034/j.1600-0706.2003.12659.x.

76.	Baraza E, Villalba JJ, Provenza FD. Nutritional context influences preferences of lambs for foods with plant secondary metabolites. Appl Anim Behav Sci. 2005;92(4):293-305. doi: 10.1016/j.applanim.2004.11.010.

77.	Chen Y, Han W, Tang L, Tang Z, Fang J. Leaf nitrogen and phosphorus concentrations of woody plants differ in responses to climate, soil and plant growth form. Ecography. 2013;36(2):178-84. doi: 10.1111/j.1600-0587.2011.06833.x.

78.	Palmroth S, Bach LH, Nordin A, Palmqvist K. Nitrogen-addition effects on leaf traits and photosynthetic carbon gain of boreal forest understory shrubs. Oecologia. 2014;175(2):457-70. doi: 10.1007/s00442-014-2923-9.

79.	Oksanen L, Oksanen T, Lukkari A, Sirén S. The role of phenol-based inducible defense in the interaction between tundra populations of the vole *Clethrionomys rufocanus* and the dwarf shrub *Vaccinium myrtillus*. Oikos. 1987;50(3):371-80. doi: 10.2307/3565498.

80.	Du Toit JT, Bryant JP, Frisby K. Regrowth and palatability of *Acacia* shoots following pruning by African savanna browsers. Ecology. 1990;71(1):149-54. PubMed PMID: ISI:A1990CN23200015.

81.	Peinetti HR, Menezes RSC, Coughenour MB. Changes induced by elk browsing in the aboveground biomass production and distribution of willow (*Salix monticola* Bebb): their relationships with plant water, carbon, and nitrogen dynamics. Oecologia. 2001;127(3):334-42. doi: 10.1007/s004420000593. PubMed PMID: WOS:000168801400006.

82.	Olofsson J, Dahlgren J, Witzell J. Grey-sided voles increase the susceptibility of Northern willow, *Salix glauca*, to invertebrate herbivory. Ecoscience. 2007;14(1):48-54. doi: 10.2980/1195-6860(2007)14[48:Gvitso]2.0.Co;2. PubMed PMID: ISI:000245364700007.

83.	Scogings PF, Hjältén J, Skarpe C, Hattas D, Zobolo A, Dziba L, et al. Nutrient and secondary metabolite concentrations in a savanna are independently affected by large herbivores and shoot growth rate. Plant Ecol. 2014;215(1):73-82. doi: 10.1007/s11258-013-0279-6.

84.	Moorby J, Wareing P. Ageing in woody plants. Ann Bot. 1963;27(2):291-308. doi: 10.1093/oxfordjournals.aob.a083846.

85.	Danell K, Huss-Danell K. Feeding by insects and hares on birches earlier affected by moose browsing. Oikos. 1985;44(1):75-81. doi: 10.2307/3544046. PubMed PMID: WOS:A1985AEJ4300013.

86.	Danell K, Haukioja E, Huss-Danell K. Morphological and chemical responses of mountain birch leaves and shoots to winter browsing along a gradient of plant productivity. Ecoscience. 1997;4(3):296-303. PubMed PMID: ISI:A1997XY28700005.

87.	Scogings P, Macanda M. *Acacia karroo* responses to early dormant season defoliation and debarking by goats in a semi-arid subtropical savanna. Plant Ecol. 2005;179(2):193-206. doi: 10.1007/s11258-004-7809-1.

88.	Löyttyniemi K. On repeated browsing of Scots pine saplings by moose (*Alces alces*). Silva Fenn. 1985;19(4):387-91.

89.	Roitto M, Markkola A, Julkunen-Tiitto R, Sarjala T, Rautio P, Kuikka K, et al. Defoliation-induced responses in peroxidases, phenolics, and polyamines in Scots pine (*Pinus sylvestris* L.) needles. J Chem Ecol. 2003;29(8):1905-18. doi: 10.1023/A:1024858413437. PubMed PMID: ISI:000184279500012.

90.	Kozlowski TT. Growth and development of trees. Volume II: Cambial growth, root growth, and reproductive growth. Ney York & London: Academic Press; 1971. XIV+514 p.

91.	Tuomi J, Niemela P, Siren S. The Panglossian paradigm and delayed inducible accumulation of foliar phenolics in mountain birch. Oikos. 1990;59(3):399-410. doi: 10.2307/3545152. PubMed PMID: ISI:A1990EQ56000015.

92.	Tuomi J, Niemelä P, Haukioja E, Sirén S, Neuvonen SJO. Nutrient stress: an explanation for plant anti-herbivore responses to defoliation. Oecologia. 1984;61(2):208-10.

93.	Myers JH, Williams KS. Lack of short or long term inducible defenses in the red alder: western tent caterpillar system. Oikos. 1987;48(1):73-8. doi: 10.2307/3565690. PubMed PMID: WOS:A1987F699000012.

94.	Hokkanen J, Mattila S, Jaakola L, Pirttila AM, Tolonen A. Identification of phenolic compounds from lingonberry (*Vaccinium vitis-idaea* L.), bilberry (*Vaccinium myrtillus* L.) and hybrid bilberry (*Vaccinium* x *intermedium* Ruthe L.) leaves. J Agric Food Chem. 2009;57(20):9437-47. doi: 10.1021/jf9022542. PubMed PMID: WOS:000270858200013.

95.	Liu P, Lindstedt A, Markkinen N, Sinkkonen J, Suomela J-P, Yang B. Characterization of metabolite profiles of leaves of bilberry (*Vaccinium myrtillus* L.) and lingonberry (*Vaccinium vitis-idaea* L.). J Agric Food Chem. 2014;62(49):12015-26. doi: 10.1021/jf503521m.

96.	Ștefănescu BE, Szabo K, Mocan A, Crişan G. Phenolic compounds from five Ericaceae species leaves and their related bioavailability and health benefits. Molecules. 2019;24(11):2046. doi: 10.3390/molecules24112046. PubMed PMID: 31146359.

97.	Seldal T, Hegland SJ, Rydgren K, Rodriguez-Saona C, Töpper JP. How to induce defense responses in wild plant populations? Using bilberry (*Vaccinium myrtillus*) as example. Ecol Evol. 2017:1-8. doi: 10.1002/ece3.2687.

98.	Moe SR, Gjørvad IR, Eldegard K, Hegland SJ. Ungulate browsing affects subsequent insect feeding on a shared food plant, bilberry (*Vaccinium myrtillus*). Basic Appl Ecol. 2018;31:44-51. doi: 10.1016/j.baae.2018.05.015.

99.	Hegland SJ, Seldal T, Lilleeng MS, Rydgren K. Can browsing by deer in winter induce defence responses in bilberry (*Vaccinium myrtillus*)? Ecol Res. 2016;31(3):441-8. doi: 10.1007/s11284-016-1351-1.

100.	Hegland SJ, Rydgren K, Seldal T. The response of *Vaccinium myrtillus* to variations in grazing intensity in a Scandinavian pine forest on the island of Svanøy. Canadian Journal of Botany. 2005;83(12):1638-44. doi: 10.1139/b05-132. PubMed PMID: 20454205.

101.	Lilleeng MS, Hegland SJ, Rydgren K, Moe SR. Ungulate herbivory reduces abundance and fluctuations of herbivorous insects in a boreal old-growth forest. Basic Appl Ecol. 2021;56:11-21. doi: 10.1016/j.baae.2021.06.006.

102.	Benevenuto RF, Seldal T, Polashock J, Moe SR, Rodriguez-Saona C, Gillespie MAK, et al. Molecular and ecological plant defense responses along an elevational gradient in a boreal ecosystem. Ecol Evol. 2020;10(5):2478-91. doi: 10.1002/ece3.6074.

103.	McKey D. The distribution of secondary compounds within plants. In: Rosenthal GA, Janzen DH, editors. Herbivores: their interaction with secondary plant metabolites. New York: Academic Press; 1979. p. 55-133.

104.	Agrawal AA, Fishbein M. Plant defense syndromes. Ecology. 2006;87(sp7):S132-S49. doi: 10.1890/0012-9658(2006)87[132:Pds]2.0.Co;2.

105.	Nordkvist M, Klapwijk MJ, Edenius L, Gershenzon J, Schmidt A, Bjorkman C. Trait-mediated indirect interactions: Moose browsing increases sawfly fecundity through plant-induced responses. Ecol Evol. 2019;9(18):10615-29. doi: 10.1002/ece3.5581. PubMed PMID: WOS:000483275700001.

106.	Wheather and climate data Norway [Internet]. Norwegian Meteorological Institute. 2021 [cited 11 January 2021]. Available from: https://seklima.met.no/observations.

107.	Tveite B, Braastad H. Bonitering for gran, furu og bjørk. Norsk Skogbruk. 1981;27(4):17-22.

108.	Nordin A, Strengbom J, Ericson L. Responses to ammonium and nitrate additions by boreal plants and their natural enemies. Environmental Pollution. 2006;141(1):167-74. doi: 10.1016/j.envpol.2005.08.017.

109.	Bajwa R, Read DJ. The biology of mycorrhiza in the Ericaceae. IX. Peptides as nitrogen sources for the ericoid endophyte and for nycorrhizal and non-mycorrhizal plants. New Phytol. 1985;101(3):459-67.

110.	Julkunen-Tiitto R, Sorsa S. Testing the effects of drying methods on willow flavonoids, tannins, ans salicylates. J Chem Ecol. 2001;27(4):779-89. doi: 10.1023/A:1010358120482.

111.	Salminen JP, Karonen M. Chemical ecology of tannins and other phenolics: we need a change in approach. Funct Ecol. 2011;25(2):325-38. doi: 10.1111/j.1365-2435.2010.01826.x.

112.	Salih EYA, Julkunen-Tiitto R, Lampi A-M, Kanninen M, Luukkanen O, Sipi M, et al. *Terminalia laxiflora* and *Terminalia brownii* contain a broad spectrum of antimycobacterial compounds including ellagitannins, ellagic acid derivatives, triterpenes, fatty acids and fatty alcohols. J Ethnopharmacol. 2018;227:82-96. doi: 10.1016/j.jep.2018.04.030.

113.	Hagerman AE. Sephadex LH 20. In: Hagerman AE, editor. The tannin handbook. Miami University, Oxford. 2002. Accessed in 2021, URL: http://www.users.miamioh.edu/hagermae/2002.

114.	Hagerman AE. Acid butanol assay for proanthocyanidins. In: Hagerman AE, editor. The tannin handbook. Miami University, Oxford. 2002. Accessed in 2021, URL: http://www.users.miamioh.edu/hagermae/2002.

115.	Nissinen K, Virjamo V, Randriamanana T, Sobuj N, Sivadasan U, Mehtätalo L, et al. Responses of growth and leaf phenolics in European aspen (*Populus tremula*) to climate change during juvenile phase change. Can J For Res. 2017;47(10):1350-63. doi: 10.1139/cjfr-2017-0188.

116.	Hagerman AE. Determination of degree of polymerization. Miami University, Oxford. 2002. Accessed in 2021, URL: http://www.users.miamioh.edu/hagermae/2002.

117.	Kao LS, Green CE. Analysis of variance: is there a difference in means and what does it mean? J Surg Res. 2008;144(1):158-70. doi: 10.1016/j.jss.2007.02.053.

118.	Pohlert T. Package ‘PMCMRplus’, calculate pairwise multiple comparisons of mean rank sums extended. R package version 1.9.6. 2022. Available from: https://CRAN.R-project.org/package=PMCMRplus.

119.	Lenth R. Package 'emmeans': Estimated Marginal Means, aka Least-Squares Means. R package version 1.3.2. 2019. Available from: https://cran.r-project.org/package=emmeans.

120.	Wickham H, Henry L, Pedersen TL, Luciani TJ, Decorde M, Lise V. Package 'svglite': An 'SVG' graphics device. R package version 2.1.0. 2022. Available from: https://CRAN.R-project.org/package=svglite.

121.	R Core Team. R: A language and environment for statistical computing. R Foundation for Statistical Computing Vienna, Austria. 2021. Available from: https://www.R-project.org/.

122.	Suvanto J, Nohynek L, Seppänen-Laakso T, Rischer H, Salminen J-P, Puupponen-Pimiä R. Variability in the production of tannins and other polyphenols in cell cultures of 12 Nordic plant species. Planta. 2017;246(2):227-41. doi: 10.1007/s00425-017-2686-8.

123.	Naima R, Oumam M, Hannache H, Sesbou A, Charrier B, Pizzi A, et al. Comparison of the impact of different extraction methods on polyphenols yields and tannins extracted from Moroccan *Acacia mollissima* barks. Industrial Crops and Products. 2015;70:245-52. doi: 10.1016/j.indcrop.2015.03.016.

124.	Hagerman AE. Extraction of tannin from fresh and preserved leaves. J Chem Ecol. 1988;14(2):453-61. doi: 10.1007/BF01013897.

125.	Ferwerda JG. Charting the quality of forage. Mapping and measuring the variation of chemical components in foliage with hyperspectral remote sensing. PhD-thesis: Wageningen Universiteit; 2005.

126.	Haukioja E, Niemelä P, Sirén S. Foliage phenols and nitrogen in relation to growth, insect damage, and ability to recover after defoliation, in the mountain birch *Betula pubescens* ssp *tortuosa*. Oecologia. 1985;65(2):214-22. doi: 10.1007/bf00379220.

127.	Stark S, Julkunen-Tiitto R, Kumpula J. Ecological role of reindeer summer browsing in the mountain birch (*Betula pubescens* spp. *czerepanovii*) forests: effects on plant defense, litter decomposition, and soil nutrient cycling. Oecologia. 2007;151(3):486-98. doi: 10.1007/s00442-006-0593-y.

128.	Ferwerda JG, van Wieren SE, Skidmore AK, Prins HHT. Inducing condensed tannin production in *Colophospermum mopane*: Absence of response to soil N and P fertility and physical damage. Plant and Soil. 2005;273(1-2):203-9. doi: 10.1007/s11104-004-7538-1. PubMed PMID: ISI:000230397500017.

129.	Schrijvers-Gonlag M, Skarpe C, Andreassen HP. Influence of light availability and soil productivity on insect herbivory on bilberry (*Vaccinium myrtillus* L.) leaves following mammalian herbivory. PLoS ONE. 2020;15(3):e0230509. doi: 10.1371/journal.pone.0230509.

130.	Jaakola L, Määttä-Riihinen K, Kärenlampi S, Hohtola A. Activation of flavonoid biosynthesis by solar radiation in bilberry (*Vaccinium myrtillus* L.) leaves. Planta. 2004;218(5):721-8. doi: 10.1007/s00425-003-1161-x.

131.	Atlegrim O, Sjöberg K. Response of bilberry (*Vaccinium myrtillus*) to clear-cutting and single-tree selection harvests in uneven-aged boreal Picea abies forests. For Ecol Manage. 1996;86(1–3):39-50. doi: 10.1016/S0378-1127(96)03794-2.

132.	Close DC, McArthur C. Rethinking the role of many plant phenolics - protection from photodamage not herbivores? Oikos. 2002;99(1):166-72. doi: 10.1034/j.1600-0706.2002.990117.x. PubMed PMID: WOS:000178883100017.

133.	Mithöfer A, Riemann M, Faehn CA, Mrazova A, Jaakola L. Plant defense under Arctic light conditions: can plants withstand invading pests? Front Plant Sci. 2022;13. doi: 10.3389/fpls.2022.1051107.

134.	Marshall C. Source-sink relations of interconnected ramets. In: Van Groenendaal J, De Kroon H, editors. Clonal growth in plants: regulation and function. The Hague: Academic Publishing; 1990. p. 23-41.

135.	Jonsdottir IS, Callaghan TV. Intraclonal translocation of ammonium and nitrate nitrogen in *Carex bigelowii* Torr. ex Schwein. using 15 N and nitrate reductase assays. New Phytol. 1990;114(3):419-28. doi: 10.1111/j.1469-8137.1990.tb00409.x. PubMed PMID: WOS:A1990CX88800008.

136.	Welker JM, Briske DD, Weaver RW. Nitrogen-15 partitioning within a three generation tiller sequence of the bunchgrass *Schizachyrium scoparium*: response to selective defoliation. Oecologia. 1987;74(3):330-4. doi: 10.1007/BF00378925.

137.	Walling LL. The myriad plant responses to herbivores. Journal of Plant Growth Regulation. 2000;19(2):195-216. doi: 10.1007/s003440000026.

138.	Mithöfer A, Wanner G, Boland W. Effects of feeding *Spodoptera littoralis* on lima bean leaves. II. Continuous mechanical wounding resembling insect feeding is sufficient to elicit herbivory-related volatile emission. Plant Physiol. 2005;137(3):1160-8.

139.	Hartley SE, Firn RD. Phenolic biosynthesis, leaf damage, and insect herbivory in birch (*Betula pendula*). J Chem Ecol. 1989;15(1):275-83. doi: 10.1007/BF02027789.

140.	Miles PW. Aphid saliva. Biol Rev. 1999;74(1):41-85. Epub 1999/02/01. doi: 10.1017/S0006323198005271.

141.	Will T, Van Bel AJE. Induction as well as suppression: How aphid saliva may exert opposite effects on plant defense. Plant Signaling & Behavior. 2008;3(6):427-30. doi: 10.4161/psb.3.6.5473.

142.	Rooke T. Growth responses of a woody species to clipping and goat saliva. African Journal of Ecology. 2003;41(4):324-8. PubMed PMID: ISI:000188226700006.

143.	Ohse B, Hammerbacher A, Seele C, Meldau S, Reichelt M, Ortmann S, et al. Salivary cues: simulated roe deer browsing induces systemic changes in phytohormones and defence chemistry in wild-grown maple and beech saplings. Funct Ecol. 2017;31:340–9. doi: 10.1111/1365-2435.12717.

144.	Liu JS, Wang L, Wang DL, Bonser SP, Sun F, Zhou YF, et al. Plants can benefit from herbivory: stimulatory effects of sheep saliva on growth of *Leymus chinensis*. PLoS ONE. 2012;7(1). doi: 10.1371/journal.pone.0029259. PubMed PMID: ISI:000301123400038.

145.	Howe GA, Jander G. Plant immunity to insect herbivores. Annu Rev Plant Biol. 2008;59(1):41-66. doi: 10.1146/annurev.arplant.59.032607.092825. PubMed PMID: 18031220.

146.	Waterman JM, Cazzonelli CI, Hartley SE, Johnson SN. Simulated herbivory: the key to disentangling plant defence responses. Trends Ecol Evol. 2019;34(5):447-58. doi: 10.1016/j.tree.2019.01.008.

147.	Clausen TP, Reichardt PB, Bryant JP, Werner RA, Post K, Frisby K. Chemical model for short-term induction in quaking aspen (*Populus tremuloides*) foliage against herbivores. J Chem Ecol. 1989;15(9):2335-46. doi: 10.1007/BF01012085.

148.	Strauss SY, Agrawal AA. The ecology and evolution of plant tolerance to herbivory. Trends Ecol Evol. 1999;14(5):179-85. PubMed PMID: ISI:000081381200007.

149.	Haukioja E, Neuvonen S. Induced long-term resistance of birch foliage against defoliators: defensive or incidental? Ecology. 1985;66(4):1303-8. doi: 10.2307/1939183.

150.	Bernards MA, Båstrup-Spohr L. Phenylpropanoid metabolism induced by wounding and insect herbivory. In: Schaller A, editor. Induced plant resistance to herbivory. Dordrecht: Springer Netherlands; 2008. p. 189-211.

151.	Furstenburg D, van Hoven W. Condensed tannin as anti-defoliate agent against browsing by giraffe (*Giraffa camelopardalis*) in the Kruger National Park. Comparative Biochemistry and Physiology Part A: Physiology. 1994;107(2):425-31. doi: https://doi.org/10.1016/0300-9629(94)90402-2.

152.	Metlen KL, Aschehoug ET, Callaway RM. Plant behavioural ecology: dynamic plasticity in secondary metabolites. Plant Cell Environ. 2009;32(6):641-53. doi: 10.1111/j.1365-3040.2008.01910.x.

153.	Roitto M, Rautio P, Markkola A, Julkunen-Tiitto R, Varama M, Saravesi K, et al. Induced accumulation of phenolics and sawfly performance in Scots pine in response to previous defoliation. Tree Physiology. 2009;29(2):207-16. doi: 10.1093/treephys/tpn017. PubMed PMID: ISI:000265849100006.

154.	Bryant JP, Heitkonig I, Kuropat P, Owen-Smith N. Effects of severe defoliation on the long-term resistance to insect attack and on leaf chemistry in six woody species of the southern African savanna. Am Nat. 1991;137(1):50-63.

155.	Scogings PF, Hjältén J, Skarpe C. Secondary metabolites and nutrients of woody plants in relation to browsing intensity in African savannas. Oecologia. 2011;167(4):1063-73. doi: 10.1007/s00442-011-2042-9. PubMed PMID: ISI:000297133800018.

156.	Cates RG, Zou J, Zeigenfuss LC, Schaalje BG, Singer FJ. Response of tannins and phenolics of willows to simulated herbivory, water dynamics, and ambient elk herbivory on the elk winter range of Rocky Mountain National Park. In: Singer FJ, Zeigenfuss LC, editors. Ecological evaluation of the abundance and effects of elk herbivory in Rocky Mountain National Park, Colorado, 1994-1999. USGS Numbered Series 2002-208. doi: 10.3133/ofr022082002. p. 157-73.

157.	Vogt T. Phenylpropanoid biosynthesis. Mol Plant. 2010;3(1):2-20. doi: 10.1093/mp/ssp106.

158.	Laine KM, Henttonen H. Phenolics/nitrogen ratios in the blueberry *Vaccinium myrtillus* in relation to temperature and microtine density in Finnish Lapland. Oikos. 1987;50(3):389-95. doi: 10.2307/3565500. PubMed PMID: WOS:A1987K854000014.

159.	Parzych A, Sobisz Z, Trojanowski J. Variability of nitrogen and phosphorus concentration and the net primary production of *Vaccinium vitis-idaea* L. and *Vaccinium* *myrtillus* L. in chosen woodland ecosystems of the Słowiński National Park. Archives of Environmental Protection. 2010;36(2):91-104.

160.	Selås V, Holand O, Ohlson M. Digestibility and N-concentration of bilberry shoots in relation to berry production and N-fertilization. Basic Appl Ecol. 2011;12(3):227-34. doi: 10.1016/j.baae.2011.01.004. PubMed PMID: ISI:000291903600005.

**Supporting information**

**S1 Annual shoots and Random selection.**

**S2 Table - Soil chemical properties.**

**S3 Standard reference curve and Tannin color test.**

**S4 Quantifying phenolics using HPLC.**

**S5 Table - Response factors.**

**S6 Table - Phenolic concentrations after simulated herbivory.**
