# Supplementary material for: Phenolic concentrations and carbon/nitrogen ratio in annual shoots of bilberry (Vaccinium myrtillus) after simulated herbivory
Source: PLoS One. 2024 Mar 4;19(3):e0298229. doi: 10.1371/journal.pone.0298229 (PMC10911626; doi:10.1371/journal.pone.0298229)
Supplement: Attachment — Submitted filename: Response to Reviewers - MSG20230804.docx [file pone.0298229.s008.docx]

PONE-D-23-06554

Phenolic concentrations and carbon/nitrogen ratio in annual shoots of bilberry (*Vaccinium myrtillus*) after simulated herbivory

PLOS ONE

**Response to Reviewers**

Dear Academic Editor,

In this rebuttal letter, I respond to points raised in the review process. First, I repeat the issue/point raised. My response follows after 'Response MSG:', font colour blue, see below.

With regards,

Marcel Schrijvers-Gonlag

Campus Evenstad, Faculty of Applied Ecology, Agricultural Sciences and Biotechnology

Inland Norway University of Applied Sciences, Koppang, Norway

marcel.schrijversgonlag@inn.no

**Journal Requirements:**

1. When submitting your revision, we need you to address these additional requirements.

Please ensure that your manuscript meets PLOS ONE's style requirements, including those for file naming. The PLOS ONE style templates can be found at
<http://www.journals.plos.org/plosone/s/file?id=wjVg/PLOSOne_formatting_sample_main_body.pdf> and <http://www.journals.plos.org/plosone/s/file?id=ba62/PLOSOne_formatting_sample_title_authors_affiliations.pdf>

Response MSG: I have studied the style templates in detail and have used these style requirements in the manuscript.

2. We note that the grant information you provided in the ‘Funding Information’ and ‘Financial Disclosure’ sections do not match. When you resubmit, please ensure that you provide the correct grant numbers for the awards you received for your study in the ‘Funding Information’ section.

Response MSG: You ask for correct grant numbers. We used funding from two grants, as stated in the Financial Disclosure. The grant number for the first one is given, but the second grant (from the Extensus Foundation) does not have a grant number. Therefore, no number is provided.

3. We note that you have stated that you will provide repository information for your data at acceptance. Should your manuscript be accepted for publication, we will hold it until you provide the relevant accession numbers or DOIs necessary to access your data. If you wish to make changes to your Data Availability statement, please describe these changes in your cover letter and we will update your Data Availability statement to reflect the information you provide.

Response MSG: no comment.

4. Please include your full ethics statement in the ‘Methods’ section of your manuscript file. In your statement, please include the full name of the IRB or ethics committee who approved or waived your study, as well as whether or not you obtained informed written or verbal consent. If consent was waived for your study, please include this information in your statement as well.

Response MSG: I have included the ethics statement in the 'Methods' section.

5. We note that Figure 1 in your submission contain [map/satellite] images which may be copyrighted. All PLOS content is published under the Creative Commons Attribution License (CC BY 4.0), which means that the manuscript, images, and Supporting Information files will be freely available online, and any third party is permitted to access, download, copy, distribute, and use these materials in any way, even commercially, with proper attribution. For these reasons, we cannot publish previously copyrighted maps or satellite images created using proprietary data, such as Google software (Google Maps, Street View, and Earth). For more information, see our copyright guidelines: http://journals.plos.org/plosone/s/licenses-and-copyright.

We require you to either (1) present written permission from the copyright holder to publish these figures specifically under the CC BY 4.0 license, or (2) remove the figures from your submission:

a. You may seek permission from the original copyright holder of Figure 1 to publish the content specifically under the CC BY 4.0 license.

We recommend that you contact the original copyright holder with the Content Permission Form (http://journals.plos.org/plosone/s/file?id=7c09/content-permission-form.pdf) and the following text:

“I request permission for the open-access journal PLOS ONE to publish XXX under the Creative Commons Attribution License (CCAL) CC BY 4.0 (http://creativecommons.org/licenses/by/4.0/). Please be aware that this license allows unrestricted use and distribution, even commercially, by third parties. Please reply and provide explicit written permission to publish XXX under a CC BY license and complete the attached form.”

Please upload the completed Content Permission Form or other proof of granted permissions as an "Other" file with your submission.

In the figure caption of the copyrighted figure, please include the following text: “Reprinted from [ref] under a CC BY license, with permission from [name of publisher], original copyright [original copyright year].”

b. If you are unable to obtain permission from the original copyright holder to publish these figures under the CC BY 4.0 license or if the copyright holder’s requirements are incompatible with the CC BY 4.0 license, please either i) remove the figure or ii) supply a replacement figure that complies with the CC BY 4.0 license. Please check copyright information on all replacement figures and update the figure caption with source information. If applicable, please specify in the figure caption text when a figure is similar but not identical to the original image and is therefore for illustrative purposes only.

Response MSG: I have removed Figure 1 completely.

**Comments to the Author**

1. Is the manuscript technically sound, and do the data support the conclusions?

The manuscript must describe a technically sound piece of scientific research with data that supports the conclusions. Experiments must have been conducted rigorously, with appropriate controls, replication, and sample sizes. The conclusions must be drawn appropriately based on the data presented.

Reviewer #1: Yes

Reviewer #2: Partly

Reviewer #3: Partly

Response MSG: The manuscript has been changed according to many reviewer recommendations (under '5. Review Comments to the Author').

2. Has the statistical analysis been performed appropriately and rigorously?

Reviewer #1: Yes

Reviewer #2: Yes

Reviewer #3: No

Response MSG: The manuscript has been changed according to many reviewer recommendations (under '5. Review Comments to the Author').

3. Have the authors made all data underlying the findings in their manuscript fully available?

The PLOS Data policy requires authors to make all data underlying the findings described in their manuscript fully available without restriction, with rare exception (please refer to the Data Availability Statement in the manuscript PDF file). The data should be provided as part of the manuscript or its supporting information, or deposited to a public repository. For example, in addition to summary statistics, the data points behind means, medians and variance measures should be available. If there are restrictions on publicly sharing data—e.g. participant privacy or use of data from a third party—those must be specified.

Reviewer #1: Yes

Reviewer #2: Yes

Reviewer #3: Yes

Response MSG: no comment.

4. Is the manuscript presented in an intelligible fashion and written in standard English?

PLOS ONE does not copyedit accepted manuscripts, so the language in submitted articles must be clear, correct, and unambiguous. Any typographical or grammatical errors should be corrected at revision, so please note any specific errors here.

Reviewer #1: Yes

Reviewer #2: Yes

Reviewer #3: No

Response MSG: The manuscript has been changed according to many reviewer recommendations (under '5. Review Comments to the Author').

**5. Review Comments to the Author**

**Reviewer #1**: The manuscript entitled Phenolic concentrations and carbon/nitrogen ratio in annual shoots of bilberry (Vaccinium myrtillus) after simulated herbivory cover an interesting topic about the allocation of phenolic compounds during differents scenarios of herbivory. It is in general well written.

However there are some minor aspects that need to be taken to be ready to publish

Why no to use another statistic test such as GLM to test interactions between location and soil productivity

Response MSG: The variable Soil productivity has been removed from the manuscript (as suggested by reviewer #3).

Don you think table 1 should be better in supplementary material?

Response MSG: Table 1 is about soil nitrogen. The variable Soil productivity has been removed from the manuscript (as directly suggested by reviewer #3 and indirectly by reviewer #1). This means that also Table 1 has been removed from the manuscript.

I think is more informative to add the letters according to a post hoc test in table two. And why are you adding the column of all treatments together? It is useful??

Response MSG: There is some overlap between Table 2 and Figure 2. I have removed Table 2 and kept the data for all threatments together Table 1 (this is useful information as it indicates the range of all data). The post hoc test letters are in Figure 2 (which is now Figure 1 in the revised manuscript).

Is it on the guidelines or why the figure legends are in the text?

Response MSG: Correct, it says in the PLoS ONE guidelines that the figure legends should be in the text (https://journals.plos.org/plosone/s/figures#loc-how-to-submit-figures-and-captions).

I don’t think that is necessary to add SE and SD from you data every time you describe the results

Response MSG: When presenting mean values, it can be very useful, although not necessary, to give information about either the spread of the data (by presenting the SD) or information about how far the mean is likely to be from the population mean (the 'real value') (by presenting the SE). For each mean value presented I have chosen to include the most appropriate parameter: the SD, the SE, or both.

I think first paragraph of discussion should be a summary of the results, why start with one particular compound?

Response MSG: I have changed the discussion, now it starts with a summary of the results.

When you make your comparisons with other systems, why do not you use related species instead of mountain birch and mopane? there are some interesting articles of other vaccinium species that can be more comparable to your study system

Response MSG: Despite an extensive search I was not able to locate papers with other *Vaccinium* species that addresses the particular topic that is discussed in this paragraph, therefore I used papers with Mountain birch and Mopane who do address the particular topic instead. To satisfy some reviewers' requests, I have removed this particular text.

*The supplementary file provided by Reviewer #1 contained many textual suggestions and some questions. I have answered the questions in the text and changed the manuscript accordingly, when appropriate. Some changes were not necessary and more a matter of taste, in these cases I often did not change my own words. As the text concerning the variable Soil productivity has been removed in the new version of the manuscript, many comments about this part can be neglected.*

**Reviewer #2**: I reviewed the paper entitled ‘Phenolic concentrations and carbon/nitrogen ratio in annual shoots of bilberry (Vaccinium myrtillus) after simulated herbivory’. Authors identified several hypotheses related to phenolics in response to simulated herbivory. Principally, related to whether these defenses follow the C:nutrient balance hypothesis or Optimal Defense Theory. The actual experimental data was quite straightforward. Various phenolic compounds and C:N ratios were measured in bilberry shoots. That being said, in my view there is excessive text and lots of speculation that goes far beyond what was tested in this manuscript. The protein competition model is quite interesting, however not properly tested. I am not sure how you can test the protein competition model without measuring any protein…There are many reasons why phenolics would be reduced or unchanged. Did you see differences in protein quantity/quality under simulated herbivory?

Response MSG: I have removed the Protein Competition Model and all text concerning proteins from the Introduction chapter and mentioned the PCM briefly in the Discussion chapter.

Further, the conclusion that this may be due to clonality is likewise interesting, but was not tested. Did you look at C:N ratios in clonal ramets connected to experimental ramets, for example, to see whether there may be a flux or allocation of resources to damaged plants.

Response MSG: Clonality is only mentioned in the Discussion section, and is not mentioned in the Conclusion section. We did not look at C:N ratios in clonal ramets as this was not the goal of our study.

Additionally, why was the foliar content not measured? Especially considering you had a defoliation treatment, one might expect differences to be most apparent in leaves. Authors should justify why this was.

Response MSG: We did not measure foliar content as our study focused on the influence of simulated herbivory (on leaves, but also more severe herbivory on shoots) on phenolic content and C:N ratio in annual shoots. One of the reasons that we focused on annual shoots instead of leaves was that many other studies have focused on leaves but studies on annual shoots were scarce.

What was actually found, as I understand, is that, other than essentially complete removal of aboveground tissue, your treatments do not reduce specific constitutive phenolics, whereas under severe damage, shoots had lower phenolics, presumably because carbon is being used for regrowth of tissue growth and is limiting. Under ambient herbivory, carbon may not be limiting due to the fact that only minor additional growth is necessary to reach static conditions, and enough is C is thus available for phenolics biosynthesis. Did you have data for changes in biomass?

Response MSG: We did measure several morphological properties of all bilberry ramets, including biomass of annual shoots, but these data will be used in another manuscript. In the reviewed manuscript we focus on chemical responses following herbivory, in a future manuscript we will focus on morphological responses following herbivory.

Although the takeaway message is rather simple, I found the MS slightly challenging to read. And was distracted by a lot of possibilities and details that were not tested in the experiments. Perhaps if the paper was reframed and simplified to highlight what was tested, as opposed to what was not (all of these trade-off theories) it would be easier to follow. For example, your first prediction is not even possible to test, because you don’t detect any gallic acid-derived phenolics at all. As such, it doesn’t make much sense to include it in the MS. Your first prediction might instead be something like ‘determine whether bilberry has these compounds at all, and compare them with Phe-derived phenolics’. Then you might be able to contextualize this in the protein competition model. However, because they are not present, this is of course not possible.

Response MSG: I have removed the first prediction and the Protein Competition Model from the Introduction chapter and mentioned the PCM briefly in the Discussion chapter. Furthermore, I have adjusted the text at many places in order to make it easier to read.

I did not leave specific, detailed comments, as lines were not numbered. In subsequent drafts I would recommend adding line numbers.

Response MSG: Line numbers were present in the manuscript but unfortunately disappeared just for submission (possibly due to a Word update). I found out about this just after submission and contacted the PLoS ONE editor instandly. I got the following reply: '... I believe the reviewers will find their way to address targeted comments. If they encounter problems regarding line numbering, I will get back to you.'
In the revised manuscript I have added line numbers.

**Reviewer #3:** After reading this study several times I can only conclude that to have a chance to be publishable, the manuscript needs to be completely rewritten to address two major problems: 1) a weak initial study design that prevents conclusions to be made with confidence followed by a modification that does little to improve the scientific integrity, and 2) a writing style that is difficult to follow due to a lack of flow and clarity arising from awkwardly constructed sentences and arguments, excessive repetition and inclusion of unnecessary text and information.
With respect strengthening the design of the study, I recommend removing the issue of high- and low productivity sites. The definition of productivity appears to be defined and supported data-wise with one variable, total nitrogen. This information comes from one composite sample per site (n=1). To merge your data and compare this to the one location which “behaved differently” also weakens your study in my opinion. I suggest you remove the idea of site productivity as a factor and rewrite the manuscript based on your “herbivory treatments”; something for which you have much better resolution and data from which to draw more confident conclusions.

Response MSG: The variable Soil productivity has been removed from the manuscript. Also, the two groups that were analysed separately in the reviewed manuscript, have been merged into one dataset, and all analyses have been adjusted accordingly, as suggested by reviewer #3. Furthermore, I have adjusted the text at many places in order to make it easier to read.

Present the plant chemistry data (phenols, tannins, N, C, and C/N) together in one Table or Figure, so that the reader can better visualize possible relationships among these variables (for example, see Nosko et al 2020, <https://doi.org/10.1016/j.foreco.2019.117839>).

Response MSG: I have taken over this suggestion and many chemistry data (tannins, all phenolics, C, N, C/N) are now in one table (Table 1).

In your discussion, you can then focus on browsing treatments as they affect the relationships among plant chemistry variables. In the absence of such relationships, some inspiration might be obtained from Warbrick et al 2020 <https://doi.org/10.1007/s11258-020-01027-y> and Nosko and Embury 2018 <https://doi.org/10.1007/s11258-018-0821-7>.

Response MSG: I have changed the discussion according to several recommendations from the reviewers.

The manuscript is very difficult to read and needs to be completely rewritten and shortened to improve readability, flow and clarity. Every element, including captions, needs to be edited and reorganized.

Response MSG: I have adjusted the text at many places in order to make it easier to read. For this, the many adjustments made by Reviewer #1 and Reviewer #3 have been very useful.

Reading through comparisons among sites/treatments is quite cumbersome (see examples on the manuscript). Flow and clarity could be greatly improved by adopting a code or acronym for your site and treatment names (see suggestions in edited manuscript).

Response MSG: The suggested codes/acronyms in the edited manuscript have been used in the revised manuscript.

Flow and length will also be improved by removing site productivity as a factor and presenting plant chemistry data together.

Response MSG: The variable Soil productivity has been removed from the manuscript and most chemistry data are now in one table (Table 1).

I did not provide editorial suggestions for the entire manuscript (see attached), but did so in several sections. Hopefully this will give you an idea of my concerns.

Response MSG: All suggestions for improvement have been considered. As with Reviewer #1, I have changed the manuscript accordingly, when appropriate.

6. PLOS authors have the option to publish the peer review history of their article ([what does this mean?](https://eur04.safelinks.protection.outlook.com/?url=https%3A%2F%2Fjournals.plos.org%2Fplosone%2Fs%2Feditorial-and-peer-review-process%23loc-peer-review-history&data=05%7C01%7Cmarcel.schrijversgonlag%40inn.no%7Cc3764d11905245fb111208db472c80a6%7Cd45cfa45904a497297413335dc0372b4%7C0%7C0%7C638182029365692210%7CUnknown%7CTWFpbGZsb3d8eyJWIjoiMC4wLjAwMDAiLCJQIjoiV2luMzIiLCJBTiI6Ik1haWwiLCJXVCI6Mn0%3D%7C3000%7C%7C%7C&sdata=M6qAgarCVG%2FwmgFyVKj%2BngnjR2U%2BKycEs%2BagtI2yVt8%3D&reserved=0)). If published, this will include your full peer review and any attached files.

If you choose “no”, your identity will remain anonymous but your review may still be made public.


**Do you want your identity to be public for this peer review?** For information about this choice, including consent withdrawal, please see our [Privacy Policy](https://eur04.safelinks.protection.outlook.com/?url=https%3A%2F%2Fwww.plos.org%2Fprivacy-policy&data=05%7C01%7Cmarcel.schrijversgonlag%40inn.no%7Cc3764d11905245fb111208db472c80a6%7Cd45cfa45904a497297413335dc0372b4%7C0%7C0%7C638182029365692210%7CUnknown%7CTWFpbGZsb3d8eyJWIjoiMC4wLjAwMDAiLCJQIjoiV2luMzIiLCJBTiI6Ik1haWwiLCJXVCI6Mn0%3D%7C3000%7C%7C%7C&sdata=lLwofVNefXLgY3PvAnpK5JZh49uQaMnTEz1sx5f1ig0%3D&reserved=0).

Reviewer #1: **Yes:** JHC

Reviewer #2: No

Reviewer #3: No
