# Supplementary material for: Phenolic concentrations and carbon/nitrogen ratio in annual shoots of bilberry (Vaccinium myrtillus) after simulated herbivory
Source: PLoS One. 2024 Mar 4;19(3):e0298229. doi: 10.1371/journal.pone.0298229 (PMC10911626; doi:10.1371/journal.pone.0298229)
Supplement: Attachment — Submitted filename: Response to Reviewers - MSG20240119.docx [file pone.0298229.s009.docx]

PONE-D-23-06554R1

Phenolic concentrations and carbon/nitrogen ratio in annual shoots of bilberry (*Vaccinium myrtillus*) after simulated herbivory

PLOS ONE

**Response to Reviewers**

Dear Academic Editor,

In this rebuttal letter, I respond to points raised in the review process. First, I repeat the issue/point raised. My response follows after 'Response MSG:', font colour blue, see below.

With regards,

Marcel Schrijvers-Gonlag

Campus Evenstad, Faculty of Applied Ecology, Agricultural Sciences and Biotechnology

Inland Norway University of Applied Sciences, Koppang, Norway

marcel.schrijversgonlag@inn.no

6. Review Comments to the Author

Reviewer #2: This is my second time reviewing this paper, and while I appreciate that the authors have made substantial revisions to their MS, which have certainly increased the overall quality and clarity of the paper, I still have some concerns regarding the data presentation and the claims made. Namely, there is substantial mention of growth, for example, lines 60, 369 and 459. While I understand authors may want to use biomass data (which they have admittedly collected) for a second MS, I find that it is an important piece of data that would make their claims and discussions more robust and compelling. Especially if the aim is to, at least in part, understand the tradeoffs between defense and other metabolic processes across variable herbivory regimes. For example, you can not say for certain that plants are prioritizing growth more or less between different defoliation treatments because you have no metric of growth (total biomass, compensatory growth). To me this is extremely relevant. One scenario I could think of is that the loss of biomass from mild herbivory treatments is effectively inconsequential for plant fitness and thus responses are not obvious. When biomass loss becomes too great, plants must begin to divert resources from other pools to compensate for biomass losses. It would be interesting to see the differences in growth after herbivory treatments, which would determine if severely damaged plants actually put on more biomass then more moderately damaged plants, or if simply the amount of resources are so low that these severely damaged plants not only produce less phenolics, but also less growth and thus metabolize less, generally. Also, while the C and N concentrations may remain stable across treatments, the total pool of each that plants have access to is not consistent; in this case it is likely a function of biomass. So the amount of carbon severely damaged plants have access to should be less then mildly-damaged plants if there is a biomass reduction.

Response MSG: we have been in contact with PLoS ONE by email (12 January 2024), as we cannot meet this point. The reply I got from PLoS ONE is below (received on 15 January 2024):

"I agree that the suggested morphometric evaluation would be an
exhausting job and further delay the manuscript publication. Although I
strongly support the Reviewer #2's demand for additional experimental
assessment, which would remarkably contribute the study
comprehensiveness, I find the manuscript in this form contains a fair
dose of novel scientific information that candidate it for acceptance
for publication if other reviewer's requirements are to be met. However,
I urge the authors to properly discuss in the text the limitations of
the present findings in light of Reviewer #2's comments."

Best regards,

Branislav

Response MSG: Therefore, I have added a paragraph in the Discussion section, called ‘Defense and other metabolic processes’, to discuss this issue.

Additionally, I find the entire conclusion section quite difficult to follow. What exactly is the takeaway message?

Response MSG: The conclusion section has been changed, to clarify our intention.

Finally, perhaps a diagram of the plant treatments would be helpful. It is difficult to determine exactly how your treatments were conducted. As such, I can't help but wonder if some of your results could be an artifact of some concentration/dilution effect by sampling different numbers of stems/ramets between treatments, that is more severe with increasing herbivory. A clear outline (visual) could help to assuage these concerns.

Response MSG: I have added a new Figure 1, which illustrates the study design.
